# Toward Intelligent Fatigue Management of Welded Metal Sheet Structures by Integrating Simulation, Sensing, and Machine Learning

**DOI:** 10.3390/ma19194156

**Published:** 2026-09-29

**Authors:** Yingjin Cheng, Zongwu Niu, Yujia Yang, Yangwei Wang

**Affiliations:** 1 National Key Laboratory of Marine Corrosion and Protection, Luoyang Ship Material Research Institute, Luoyang 471023, China; 2 Marine Science and Technology Domain, Beijing Institute of Technology, Zhuhai, Zhuhai 519088, China; 3 National Key Laboratory of Science and Technology on Materials Under Shock and Impact, Beijing Institute of Technology, Beijing 100081, China

**Keywords:** welded metal sheet structures, fatigue failure, fatigue life prediction, fatigue crack propagation, multi-scale modeling, structural health monitoring

## Abstract

Welded metal sheets are used across automotive, aerospace, marine, and civil infrastructure applications for their high strength and light weight, yet fatigue failure remains a persistent concern in relation to their long-term structural stability. Fatigue crack growth is strongly influenced by the interaction of weld geometry and manufacturing-related defects. This review presents a comprehensive overview of fatigue-life prediction approaches, outlining the welding processes, fatigue mechanisms, computational tools, and progress made in predictive approaches central to this field. Fatigue performance is evaluated across several welding approaches, including laser beam, resistance spot, tungsten inert gas, and hybrid laser–arc welding. Advanced computational approaches for predicting fatigue life, including finite element modeling, cohesive zone modeling, crystal plasticity modeling, and multi-physics/multi-scale simulation, are also examined. Recent innovations in experimental characterization and structural health monitoring are addressed in detail. The review further highlights how machine learning-enabled digital twins and sensor fusion frameworks improve fatigue damage detection and maintenance prediction. Unlike prior reviews that treat conventional and data-driven methods separately, this work integrates welding process selection, fatigue life prediction, experimental validation, and structural health monitoring within a single closed-loop simulation–sensing–machine learning framework specific to thin welded sheets. Despite such advancements, the accurate prediction of environmental degradation and microstructural evolution remains challenging, and future research is required to integrate physics-based and data-driven models for critical welded structures.

## 1. Introduction

Welding is a foundational joining method in modern industrial manufacturing because it produces strong, reliable, and durable bonds between metallic components. The growing demand for lightweight and cost-effective engineering components has increased the importance of welded metal sheets relative to other structural forms [1]. Thin-sheet welded assemblies are used extensively in automotive body structures, aircraft wings, railway carriages, shipbuilding, bridges, pressure vessels, household products, and renewable energy systems. Their high load-bearing capacity, design flexibility, and suitability for mechanized mass production are the primary reasons for their widespread adoption [2].

Despite these advantages, welded joints are often regarded as the most vulnerable regions of metallic structures because the welding process significantly alters the material’s properties and structural strength. The localized heating and rapid cooling associated with welding produce complex thermal cycles that change the mechanical properties, residual stress distribution, and microstructure of both the weld metal and the adjacent heat-affected zone (HAZ). These kinds of changes can make welded joints more vulnerable to fatigue degradation than the parent material, as they introduce geometric discontinuities, localized stress concentrations, and metallurgical variations. As these structures are constantly exposed to cyclic loading in service, fatigue failure has remained the principal cause of unexpected structural failure in welded components [3]. This review presents an in-depth analysis of fatigue life prediction in welded metal sheet structures covering the conventional methods, combined with up-to-date theoretical approaches and recently established intelligent prediction systems. It also highlights the existing research gaps, assesses the strengths and limitations of prediction approaches, and discusses potential pathways of an integrated fatigue assessment framework that combines computational modeling with materials science.

**Figure 1:**
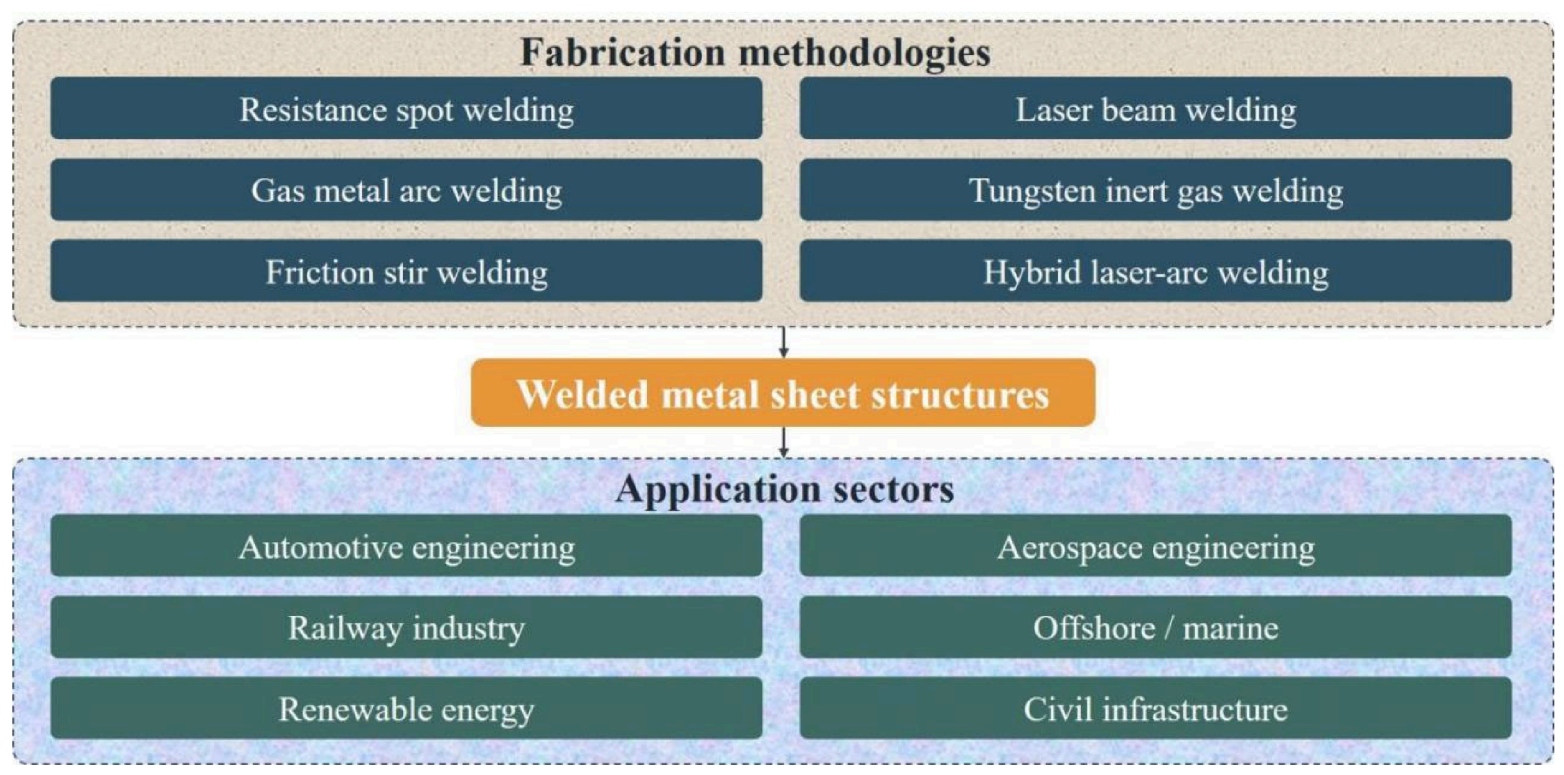
Fabrication techniques, i.e., laser beam, resistance spot, TIG, GMAW, friction stir, hybrid laser–arc welding and the utilization of welded metal sheet structures in industry sectors, automotive, aerospace, railway, offshore/marine, renewable energy and civil infrastructure [4].

Figure 1 presents a flowchart of the fabrication techniques available for welded metal sheet structures, along with their use in different applications. It underpins the synthesis of metal sheet welding across lightweight structure industries, combining fusion methods like gas metal arc welding, resistance spot welding, laser beam welding, tungsten inert gas welding and hybrid laser–arc welding with friction stir welding. Solid state methods are utilized for lowering energy usage, reducing porosity, and removing the necessity for consumable electrodes, whereas the friction stir spot welding method can offer a variety of advantages compared with other processes.

Such welded structures are utilized in aerospace, automotive, offshore and marine, railway, renewable energy, and civil infrastructure sectors. Because aluminum (Al) offers a favorable strength-to-weight ratio, the spot welding of aluminum is widely implemented, providing the opportunity to achieve mass reduction and structural performance goals for such applications [4].

A note on scope before proceeding: this review centers on welded metal sheet structures, and the prediction methods, experimental techniques, and case studies discussed throughout are selected and framed with thin-to-medium-gauge sheet applications as the primary reference point. Where the discussion draws on literature involving comparatively thick plates, bridges, pressure vessels, or additive-manufactured components, this is a deliberate choice rather than a scope drift; the underlying fatigue prediction methods (S-N, fracture mechanics, digital twin, and machine learning approaches alike) are largely shared across thickness regimes and joint types, and the thick-section literature is used specifically where it demonstrates a technique, a validation dataset, or an industrial deployment that is directly transferable to sheet structure applications, or where it establishes a methodological point (e.g., digital twin technology-readiness, sensor fusion validation) for which sheet-specific studies remain scarce. Sections that draw on this broader literature note the connection back to sheet structures explicitly where it is not self-evident.

### 1.1. Background and Importance of Structures Made of Welded Metal Sheets

Nowadays, metal sheet structures are considered important parts of modern engineering, as they combine low mass, high mechanical strength and fast manufacturing ability. The rising significance of CO_2_ emission reduction, clean energy efficiency and sustainable production has compelled a considerable upsurge in demand for thin metal sheets in various industries. In contrast with thick structures, thin metal sheets allow engineers to construct lightweight structures without losing structural toughness, provided that the welding methods provide dependable and long-lasting connections [5]. The automotive sector is the largest user of welded sheet structures; laser beam welding alone creates thousands of joints in typical passenger vehicles, connecting chassis components, body pillars, crash structures, and reinforcements to deliver overall vehicle rigidity and protection [6]. Manufacturers are increasingly using Al-based alloys, high-strength steels, and composite structural frameworks for reducing weight. Understanding fatigue behavior in such materials matters directly for this shift toward lightweighting, since lightweight materials demonstrate different welding responses and fatigue properties compared to conventional carbon steels [7].

### 1.2. Importance of Fatigue Life Prediction in Engineering Applications

Fatigue is considered the most important cause of failure in structural engineering components subjected to continuous loading. It differs from static failure, which takes place when the applied load surpasses the material’s ultimate strength; fatigue failure grows gradually under cyclic stresses that are typically well below the material’s yield strength [8]. Because fatigue damage accumulates gradually and is often not detected until late in the crack-propagation process, it poses a significant risk to structural safety and reliability. Fatigue is particularly important in welded metal sheet structures because the stress distribution and geometric discontinuities introduced during welding can significantly accelerate crack initiation and propagation. Accurate fatigue life prediction has therefore become an essential part of risk management, structural integrity assessment, and engineering design [9].

Fatigue life prediction is beginning to evolve from a static design calculation toward a dynamic, real-time monitoring capability with the emergence of digital twin technology (virtual model of a physical structure that is continuously updated using sensor data), combining real-time operational data, machine learning methods, and finite element models. In principle, such a model can track the progress of fatigue damage throughout the service life of a welded structure, and this capability is widely regarded as a key enabler of smart manufacturing, supporting predictive maintenance and early failure warning [10]. As discussed in Section 8, however, fully integrated fatigue digital twins of this kind remain at an early, largely research-stage level of technological readiness for welded metal sheet structures specifically; demonstrated implementations to date address individual components of the framework on specific structures rather than a complete, general-purpose closed-loop system. The stakes involved diverge markedly from one sector to another, and Table 1 summarizes how the dominant fatigue loading, the practical importance of prediction, and the consequences of failure vary across six major industries, from vehicle crashworthiness in automotive engineering to public safety in civil infrastructure.

This comparison shows that although the dominant fatigue-loading mechanism differs from one sector to another, welded structures in every industry listed share the same underlying vulnerability to cyclic damage, which is why the prediction, monitoring, and modeling approaches reviewed in the remainder of this paper are presented as broadly applicable methods rather than as solutions tailored to a single application area.

**Figure 2:**
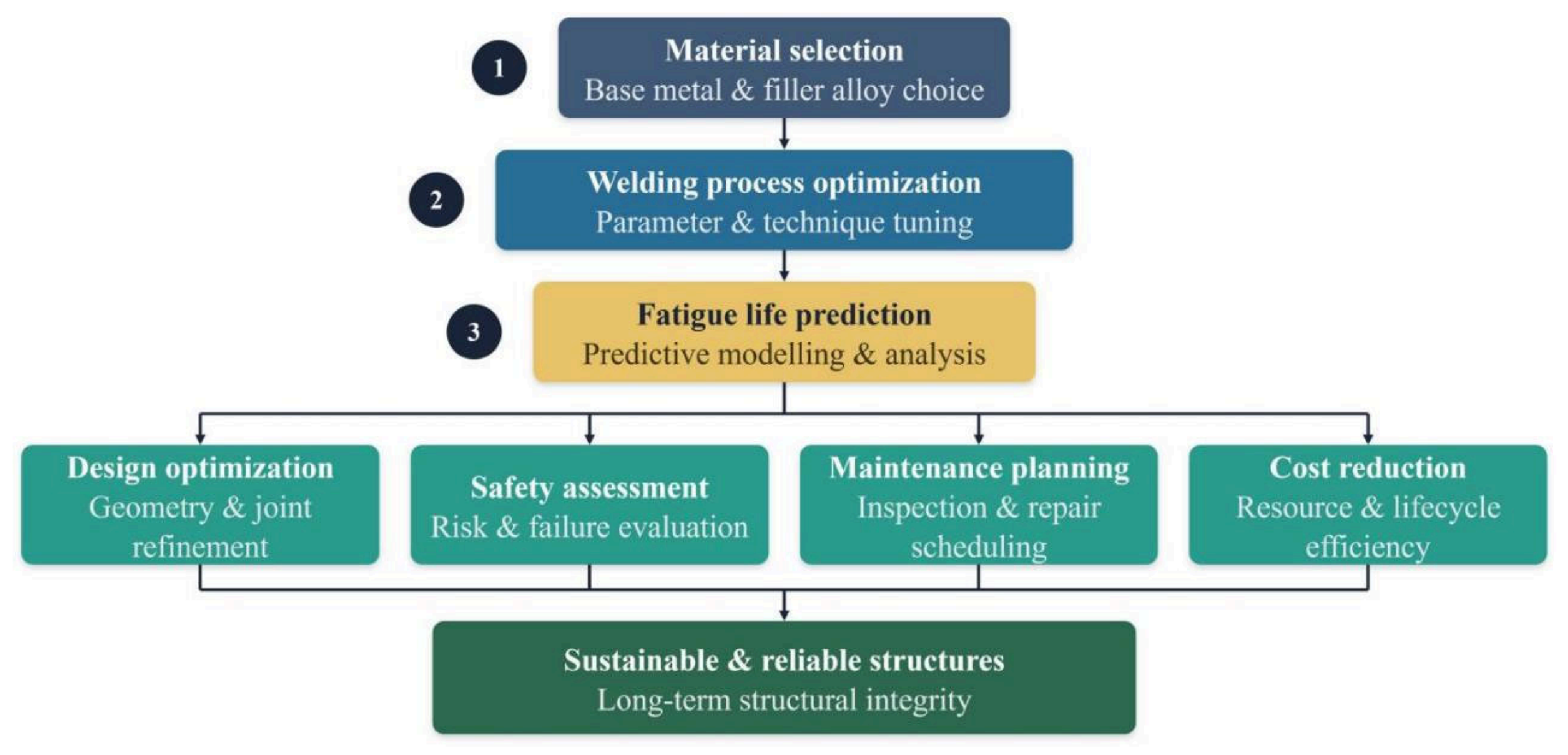
Importance of fatigue life prediction in material engineering decision-making.

Figure 2 shows that fatigue life prediction should not be treated as an isolated characterization exercise, but rather as the central stage of a decision pipeline; it begins with material selection and welding process optimization, is followed by predictive modeling and analysis, and finally drives design optimization, safety assessment, maintenance planning, and cost reduction.

### 1.3. Mechanisms of Fatigue Failure in Welded Sheet Structures

Fatigue failure in welded sheet structures arises from the interaction of mechanical loading, geometric discontinuities, material properties, and environmental factors. Fatigue damage accumulates gradually and often goes unnoticed until a critical crack size is reached, making it one of the most severe failure modes in structural engineering [19]. These mechanisms rarely occur in isolation, and Table 2 compares the six most common failure mechanisms in welded sheet structures, highlighting where they originate, how they grow, and the mitigation strategy most effective against each.

Table 2 shows that the weld toe, weld root, and heat-affected zone are the major origin points of fatigue failure across all mechanisms, and that geometrical, residual stress, and manufacturing defect factors are the primary levers for improving the fatigue performance of welded sheet structures (discussed further in Section 4).

### 1.4. Current Challenges in Fatigue Assessment

The fatigue assessment of welded metal sheet structures has gained considerable attention from researchers over the past few decades, driven by increasing demand for strong, lightweight, and reliable engineering components. Fatigue assessment research in this area spans experimental characterization, numerical simulation, and artificial intelligence-based prediction, all of which have advanced significantly over the past few decades [26].

**Figure 3:**
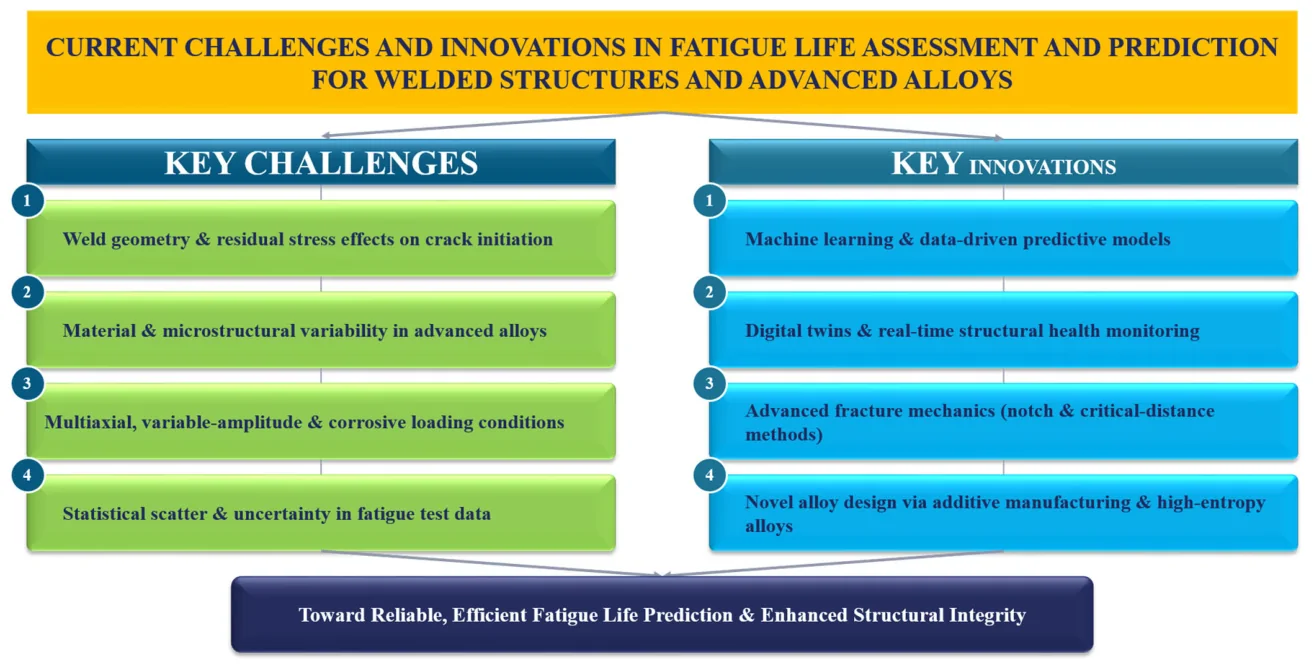
Current challenges and innovations in fatigue life assessment and prediction.

Estimating the fatigue life of welded joints without error remains a significant engineering and scientific challenge because of the simultaneous interaction of several physical phenomena, including microstructural irregularities and residual stresses that operate across multiple physical and temporal scales. This is why no single fatigue prediction method exists that can fully account for all relevant influencing factors under service conditions. One of the promising future directions is the development of AI-based fatigue assessment frameworks that combine finite element modeling, structural rigidity monitoring, digital twins, and real-time sensor data to continuously assess structural condition throughout the service life of welded metal components. Such frameworks are expected to support predictive maintenance and autonomous decision-making, allowing the better prediction of crack initiation, propagation, and practical service life. Realizing this vision will require standardized databases, robust procedures, better computational efficiency, and closer collaboration between materials scientists, welding engineers, computational analysts, and artificial intelligence researchers [27]. Figure 3 draws these links together, by mapping the four convergence zones, multiaxial and proportional loading, variable-amplitude loading and interpretation, severe environments and corrosion, and advanced materials and manufacturing, onto the computational and interpretability bottlenecks that currently limit fatigue assessment, and onto the physics-based, high-fidelity pillars that a unified future framework will need to combine.

### 1.5. Design Codes and Standards for Fatigue Assessment

The fatigue design of welded metal structures is governed by a well-established set of international codes that translate underlying physical mechanisms into design rules usable by practicing engineers. The most widely recommended document is the International Institute of Welding (IIW) recommendations for the fatigue design of welded joints, which provide detail-specific S-N curves (FAT classes) covering nominal and effective notch stress approaches for a very wide range of joint geometries [28]. These recommendations have evolved considerably and have been continuously refined since the 1990s [29]; more recent work has extended them to the reference radius-based assessment of complex joint details [30]. Corresponding design codes are also developed and used in specific industry sectors; DNV-RP-C203 governs the fatigue design of offshore steel structures such as jackets, floating platforms, and wind turbine substructures [31]; Eurocode 3 provides fatigue design rules for steel building and bridge structures within the European regulatory framework [32]; and AWS D1.1 is widely used for structural steel welding fatigue provisions in North America [33].

Weld quality itself is governed by acceptance level standards; one of the most common is ISO 5817, which classifies imperfections (undercut, porosity, lack of fusion, misalignment) into quality levels B, C, and D depending on the defect sizes expected in fracture mechanics-based fatigue assessments [34]. Pressure-retaining welded equipment is additionally governed by the design-by-analysis fatigue rules of the ASME Boiler and Pressure Vessel Code, Section VIII, Division 2 [35].

Because these codes use different levels of conservatism, safety factors, and assumed defect populations, the consistent use of a single code throughout the entire project is essential, and mixing S-N data from one code with detail categories from another is a common and preventable source of error in industrial fatigue assessment.

### 1.6. Related Review Articles and Positioning of This Work

Two recent review articles occupy adjacent ground to the present work. Kang and Luo systematically reviewed uniaxial and multiaxial fatigue life prediction models for welded joints, with particular emphasis on low-cycle fatigue and ratchetting behavior, but their review predates the recent wave of machine learning, digital twin, and structural health-monitoring methods that form a substantial part of the present review [36]. Feng et al. reviewed the machine learning applications in welded-joint fatigue prediction with a focus on model architectures and dataset characteristics [37]. The present review differs from both in scope; rather than treating conventional and data-driven methods separately, it follows welded metal sheet structures across the full pipeline from welding-process selection and influencing factors, through conventional and machine learning prediction methods, to experimental characterization, structural health monitoring, and emerging sustainability-driven and intelligent fatigue-management practices, within a single sector-spanning framework.

To make the incremental contribution of the present review explicit, Table 3 benchmarks it against the two reviews discussed above and three additional closely related works; a review of fatigue assessment methods for welded steel structures [38], a comprehensive review of neural network-based fatigue modeling [39], and a review of very-high-cycle fatigue (VHCF) of welds [40]. The comparison is kept deliberately conservative; a dimension is marked as covered for a competing work whenever that work treats it as a principal focus, even where its treatment is more detailed than the corresponding treatment in the present review. To make this classification reproducible rather than a purely qualitative judgment, each work was assigned “principal focus” (√) for a given dimension when at least one full section or sub-section (typically ≥1 page) was devoted specifically to that dimension; “background only” (~) when the dimension was mentioned only in passing, in an introductory paragraph, or as contextual support for a different focus (typically <0.5 page, with no dedicated sub-section); and “not covered” (–) when the dimension did not appear in the work at all. This page/section-based rule was applied consistently across all six works, including the present review, to keep the comparison in Table 3 auditable and reproducible by other readers.

This work is a narrative, rather than a systematic, review, reflecting its intentionally broad scope across welding processes, conventional and machine learning fatigue prediction methods, structural health monitoring, and sustainability-driven fatigue management; a formal PRISMA-style review was therefore not adopted. Representative search strings used across the three databases include (“welded joint*” OR “welded steel” OR “welded sheet*” OR “welded metal sheet*”) AND (fatigue OR “fatigue life” OR “fatigue crack” OR “crack growth” OR “crack propagation”) AND (welding OR “spot weld*” OR “laser weld*” OR “friction stir weld*” OR “arc weld*”), supplemented by parallel string sets substituting the fatigue-related terms with “machine learning,” “digital twin,” “structural health monitoring,” or “residual stress” to capture the corresponding sub-topics. Records were included if they were peer-reviewed journal articles, conference papers, or recognized design/fatigue standards addressing welded metallic structures and reporting fatigue-relevant experimental, computational, or predictive results in English; records were excluded if they addressed unwelded or non-metallic structures, fatigue-unrelated aspects of welding (e.g., purely metallurgical or corrosion studies with no fatigue component), or were not accessible in full text. Applying these strings across Scopus, Web of Science, and Google Scholar for the 2000–2026 window returned approximately 650 unique records after de-duplication; title/abstract screening against the inclusion criteria above narrowed this to approximately 260 records, and full-text screening for direct relevance to welded metal sheet fatigue yielded the 175 references cited in this review.

## 2. Fundamentals of Fatigue in Welded Metal Sheets

**Figure 4:**
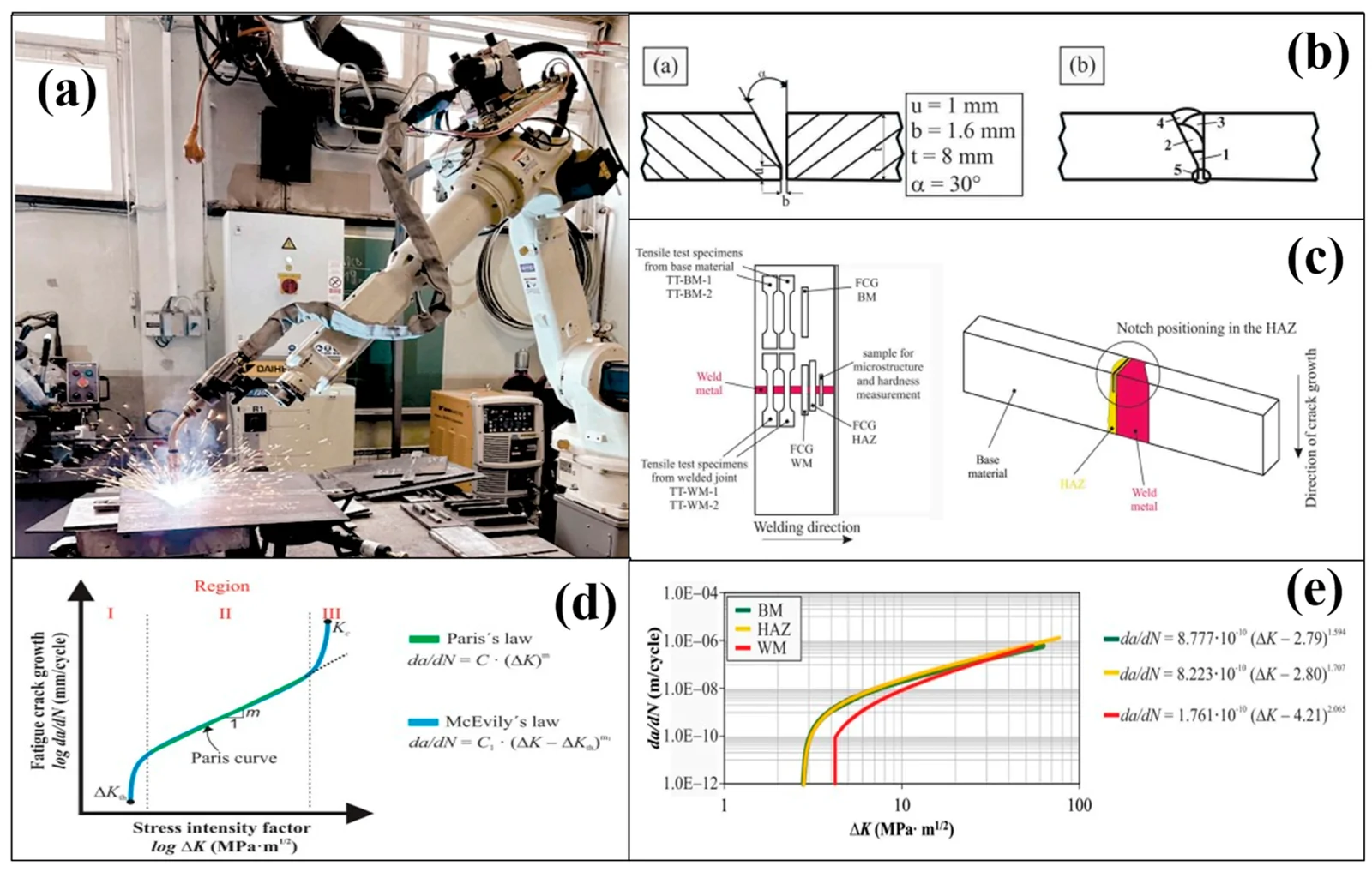
Experimental and theoretical framework for fatigue crack growth (FCG) in welded steel joints (adapted from [41] under CC-BY), (**a**) robotic GMAW welding cell used to produce the joint, (**b**) weld groove geometry (u = 1 mm, b = 1.6 mm, t = 8 mm, α = 30°), (**c**) specimen-extraction scheme for BM, HAZ, and WM, with the notch positioned in the HAZ, (**d**) schematic da/dN−ΔK curve showing Regions I–III, with Paris’s and McEvily’s laws, (**e**) measured da/dN−ΔK data and fitted growth law parameters for BM, HAZ, and WM (adapted from [41] under CC-BY).

The interaction between joint geometry, microstructural heterogeneity across base metal (BM), heat-affected zone (HAZ), weld metal (WM), and crack growth mechanics that follow from these regions defines fatigue behavior in welded metal sheets. Figure 4 provides a graphical overview of the experimental and theoretical framework, drawn from a recent study on armor steel welded joints produced using an automated robotic GMAW welding cell (Figure 4a) [41]. The joint geometry, combined with the specimen extraction strategy and crack growth behavior, is illustrated in this figure and referenced throughout the following subsections to ground the discussion of fatigue fundamentals in representative experimental data.

Figure 4a shows the robotic gas metal arc welding (GMAW) cell used to weld the armor steel joint, providing automated control over travel speed, torch angle, and heat input along the weld [41]. This process consistency minimizes weld-to-weld variability, isolating joint geometry and metallurgical zone (base metal (BM), heat-affected zone (HAZ), weld metal (WM)) as the primary factors governing fatigue behavior.

### 2.1. Influence of Stress Concentration on Welds

Stress concentration is one of the key factors governing fatigue performance in welded metal sheets. Geometric discontinuities introduced by welding, such as weld roots, weld toes, reinforcement, and abrupt changes in cross-section, increase the local stress well above the nominal applied stress. Compared with unwelded material, these stress concentrations significantly reduce fatigue strength and promote early crack initiation. Stress concentration levels are further increased by factors such as poor weld quality, residual stresses, and welding defects. Techniques such as weld toe grinding, hammer peening, TIG dressing, and improved weld geometries are used to reduce stress concentration and increase fatigue resistance. The precise assessment of stress concentration factors (SCFs) is therefore vital to fatigue design and life prediction [42]. Figure 4b shows a representative single-V groove geometry (root gap u = 1 mm, root face b = 1.6 mm, plate thickness t = 8 mm, bevel angle α = 30°), with a configuration chosen specifically to preserve the base metal’s mechanical properties as closely as possible while minimizing geometric discontinuities at the weld toe and root [41].

### 2.2. Fatigue Crack Initiation Mechanisms

Fatigue crack initiation, the first phase of fatigue failure, is characterized by the formation of microscopic cracks under repeated cyclic stress; it consistently occurs in regions of welded metal sheets where stress is concentrated, such as the weld root or toe, porosity, inclusions, or other welding defects. Persistent slip bands form within the material due to localized plastic deformation caused by repeated stress cycling [20] and then develop into microcracks that gradually grow under continued loading. In plain (unwelded) specimens, the initiation phase can account for a large proportion of total fatigue life; however, in welded joints containing defects, crack initiation occurs much more quickly because these flaws act as ready-made crack starters. Figure 4c illustrates a representative specimen-extraction scheme in which compact tension specimens are taken separately from each zone, with the notch deliberately positioned within the HAZ to isolate its initiation response from the adjacent regions. This approach reflects the broader consensus that the HAZ, owing to grain coarsening and localized hardness gradients introduced during welding, is frequently the preferred site of fatigue crack initiation in welded steel structures [41].

### 2.3. Fatigue Crack Propagation

Once a fatigue crack forms, it enters the propagation stage, in which it steadily extends with each loading cycle until complete fracture occurs. Many factors, including the applied stress range, the stress concentration factor, and the surrounding environment, influence the rate of crack growth. The tensile residual stress generated in welded metal sheets also tends to accelerate crack propagation by reducing the beneficial effect of crack closure. Fatigue crack growth generally proceeds through three main stages: a slow initial growth region, a stable growth region described by Paris’ law, and a rapid, unstable growth phase that ends in fracture. Understanding crack propagation behavior is therefore crucial for predicting the remaining service life of welded structures and for planning maintenance before failure occurs [21]. These three stages are illustrated in Figure 4d, which shows the classical three-region da/dN–ΔK curve, comprising the near-threshold (Region I), stable Paris law (Region II), and unstable fracture (Region III) portions, with McEvily’s law additionally shown to capture near-threshold behavior more accurately than Paris’s law alone [21,41]. Experimental crack growth data for the three welded joint zones (Figure 4e) further support this framework, showing that the HAZ exhibits the lowest threshold stress intensity and the earliest onset of stable growth, while the WM shows the steepest growth rate exponent at higher ΔK, accounting for its greater susceptibility to accelerated failure under high-amplitude cyclic loading [41]. Furthermore, Table 4 breaks this discussion into four discrete phases—crack initiation, stable crack growth, accelerated crack growth, and final fracture—summarizing the metallurgical characteristics and structural integrity consequences that differentiate each phase, and linking the crack growth rate curves of Figure 4d,e with the practical detection and inspection cues associated with every stage.

By pairing each propagation phase with a practical detection cue, Table 4 establishes the link between the crack growth mechanics described above and the structural health-monitoring techniques discussed in Section 7, since whether a crack is caught in Phase B rather than Phase C or D determines whether a timely repair is possible or whether the component must be replaced.

Phase B (“stable crack growth”) is treated as a single, mechanistically uniform stage governed by Paris’ law, whereas it is conventional in the fatigue literature to further distinguish short-crack propagation from long-crack propagation, since the two are governed by different physical mechanisms and are not equally well served by the same predictive method. This distinction and its consequences for selecting among the S-N, strain life, fracture mechanics, cohesive zone, XFEM, and monitoring based approaches introduced later in this review are developed explicitly in Section 6.10.

### 2.4. Influence of Cyclic Loading Conditions

The fatigue life of welded metal sheets is also influenced by the characteristics of cyclic loading, stress amplitude, mean stress, loading rate, stress ratio, and load sequence. Higher stress amplitudes increase crack initiation and propagation, and therefore reduce fatigue life, while compressive mean stresses can control crack initiation and tensile mean stresses shorten fatigue life by increasing the effective stress intensity. Real engineering assemblies typically experience variable-amplitude loading, which introduces overloads and underloads that alter crack growth behavior and make fatigue life more difficult to predict. Many combined actions of cyclic stress and environmental factors such as corrosion and elevated temperature can also accelerate fatigue damage, hence the accurate classification of the loading spectrum is essential for reliable fatigue assessment [17].

Converting a real, irregular load time history into a form the prediction methods of Section 6 can use requires cycle counting, almost universally via the rainflow counting algorithm, which pairs stress reversals into closed hysteresis loops so that each counted cycle can be assigned a range and mean stress and combined with Miner’s rule (Section 2.5) or an equivalent damage accumulation model. This counting step is not merely bookkeeping; rainflow counting discards the sequence in which cycles occurred, whereas the actual order of high- and low-amplitude cycles measurably affects fatigue life through load-interaction effects that sequence-blind damage summation cannot capture. A single tensile overload, for example, generates a local plastic zone at the crack tip that leaves behind compressive residual stress once the overload is removed, retarding subsequent crack growth for some distance until the crack grows through the affected zone—an effect captured empirically by retardation models such as the Wheeler and Willenborg approaches, which modify the baseline Paris law growth rate by a retardation factor that decays as the crack advances past the overload-affected zone. Underloads and compressive excursions can partially or fully negate this retardation, and realistic service spectra mix both, so the net effect of a given spectrum on fatigue life is generally not predictable from its range and mean stress statistics alone. Because of this, spectrum-loading fatigue tests—typically run against standardized load sequences such as FALSTAFF (fighter aircraft loading) or WISPER/WISPERX (wind turbine blade loading) rather than a single laboratory-convenient spectrum—remain the primary evidence base for variable-amplitude behavior in welded joints, and this evidence base is markedly smaller and more application-specific than the constant-amplitude S-N data underlying Section 6.1 and Section 6.2, since a spectrum validated for one loading environment and joint type does not necessarily transfer to another. Variable-amplitude behavior therefore remains one of the less standardized and more evidence-limited areas of fatigue assessment covered in this review, notwithstanding its practical importance for the real engineering assemblies noted above.

### 2.5. Theories of Fatigue Damage Accumulation

The damage accumulation models of fatigue describe how a material progressively weakens under repeated loading until failure occurs. The most widely used approach is Miner’s linear damage rule, which states that fatigue failure occurs once the cumulative damage reaches unity and that total fatigue damage is the sum of the damage contributed by individual stress cycles [45]. It is a simple and widely used approach, but it has certain limitations, such as not accounting for interaction among different stress levels or load-sequence effects. To provide more accurate predictions of fatigue under variable-amplitude loading, alternative approaches, including nonlinear damage models, continuum damage mechanics (CDM), and energy-based approaches, have been developed. These approaches are particularly beneficial for welded metal sheets, where complex loading histories and welding defects strongly influence damage accumulation [46]. The competing crack growth formulations shown in Figure 4d, i.e., Paris’s law given as da/dN=C(ΔK)m and McEvily’s law given as $da/dN=C_{1}(\Delta K-{\Delta K}_{th})m_{1}$, represent two established theoretical frameworks to describe how fatigue damage accumulates with cycling in welded joints [41]. Paris’s law is widely used because its process of predicting the stable growth regime is simple, while McEvily’s law is more complex and it provides a more accurate description near the threshold, where the finite value of ΔK_th_ becomes significant for predicting the onset of measurable crack growth [47].

### 2.6. Physical Metallurgy of the Weld and Heat-Affected Zone

The fatigue behavior of a welded joint cannot be fully separated from the physical metallurgy of the weld itself. During fusion welding, the weld metal solidifies from a molten pool through a sequence of nucleation and dendritic growth stages that are strongly influenced by cooling rate, alloy composition, and shielding conditions, producing a cast microstructure that differs markedly from the wrought, rolled microstructure of the parent sheet [41]. Adjacent to the fusion boundary, the heat-affected zone undergoes solid state transformations without melting; in low-carbon and low-alloy steels, this typically produces a sequence of coarse-grained, fine-grained, and inter-critical sub-zones, each with different hardness, toughness, and susceptibility to hydrogen-assisted cracking, while in age-hardenable aluminum alloys the HAZ instead experiences localized over-aging and the dissolution of strengthening precipitates, producing a pronounced local softening that is often the true fatigue-critical location rather than the weld toe itself [41]. Superimposed on these microstructural changes are the residual stresses and distortions generated by the highly non-uniform temperature field during welding, which Masubuchi’s classical analysis showed to depend on the interaction between the moving heat source, the restraint provided by surrounding material, and the sequence in which welds are deposited [48]. A complete understanding of the metallurgical and thermo-mechanical background is a prerequisite for interpreting why nominally identical weld geometries can show very different fatigue lives when produced with different processes, consumables, or heat inputs. While Masubuchi’s original analysis remains the conceptual foundation for understanding welding-induced residual stress, the quantitative prediction of these fields is now handled by the coupled thermo-mechanical simulations described in Section 5.2 and Section 5.3, and their consequences for fatigue life, including the dependence on heat input of the HAZ microstructure and strength, are examined further in Section 4.5.

## 3. Effects of Welding Processes on Fatigue Performance

### 3.1. Resistance Spot Welding (RSW)

**Figure 5:**
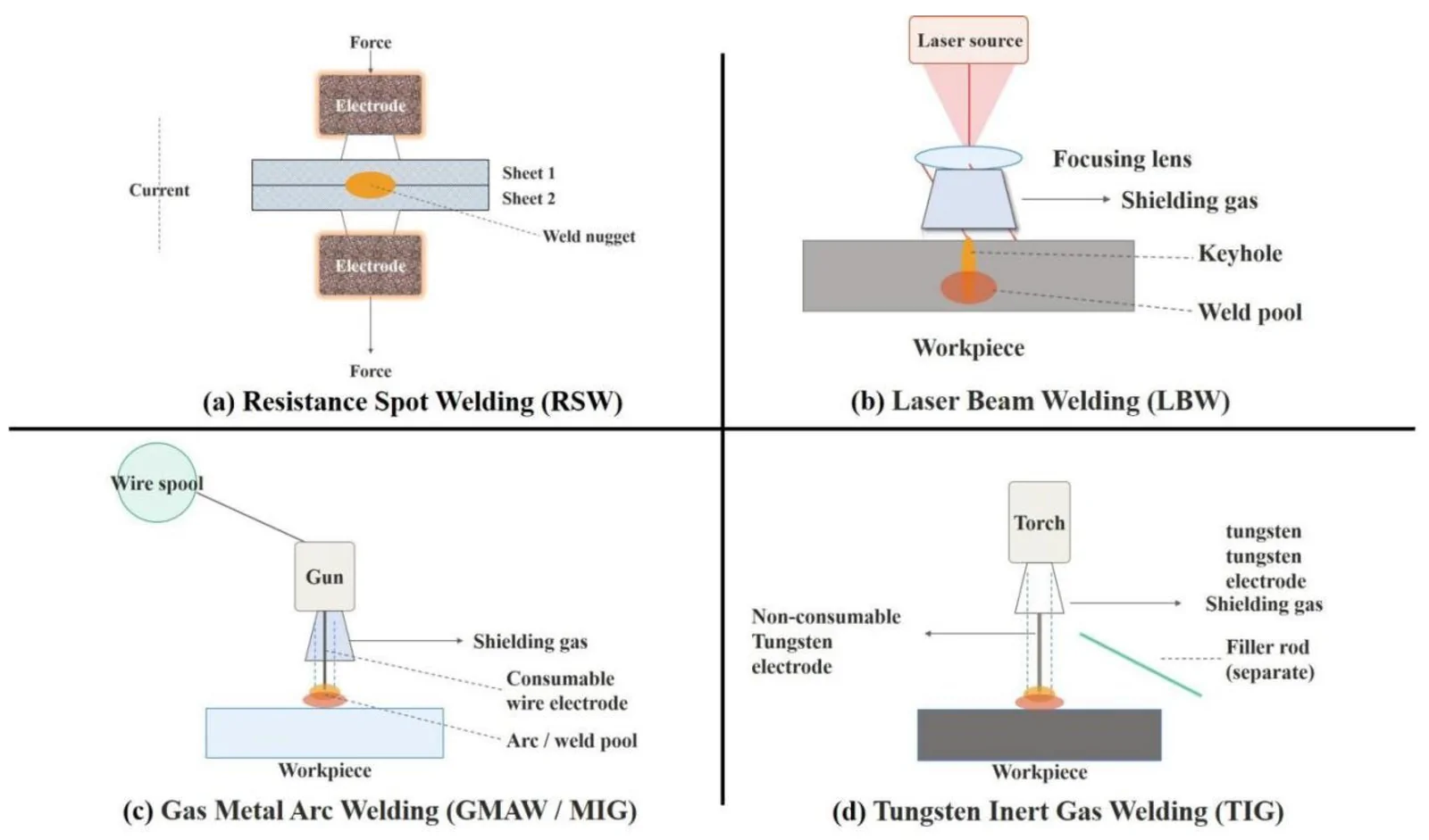
Schematics of the four welding processes reviewed in this section. (**a**) RSW: electrodes clamp two sheets under force and pass current through the interface, forming a weld nugget by resistive heating. (**b**) LBW: a focused laser beam drills a keyhole through the workpiece under shielding gas, forming a weld pool beneath it. (**c**) GMAW/MIG: a continuously fed consumable wire electrode forms the arc and supplies filler metal simultaneously, shielded by gas from the gun nozzle. (**d**) TIG/GTAW: a non-consumable tungsten electrode sustains the arc under inert gas shielding, with filler metal added separately via a hand-fed rod.

Resistance spot welding (RSW) is one of the most widely used processes for joining thin metal sheets, particularly in the automotive and aerospace industries, in which a weld nugget is formed by compressing two or more metal sheets between copper electrodes while a high electric current passes through the contact area, generating heat through electrical resistance [49]. The performance of resistance-spot-welded joints depends on certain factors (nugget diameter, welding current, sheet thickness, electrode force, and weld spacing), because each spot weld acts as a localized stress concentrator. Despite the high production efficiency of RSW, combined with its low manufacturing cost, its fatigue strength is typically lower than that of continuous welded joints [50,51]. As shown in Figure 5a, two electrodes clamp the overlapped sheets under force and pass a high current through the interface, with resistive heating forming the weld nugget between them.

### 3.2. Laser Beam Welding (LBW)

LBW uses a focused, high-energy-density beam that melts and joins the welding sheets together with high precision; compared with conventional welding methods, LBW reduces thermal damage and residual stresses by producing a narrow fusion zone and a relatively small heat-affected zone [52]. This technique has shown superior performance due to its high quality and smooth weld profile, especially in high-cycle fatigue regions. However, certain defects, including incomplete penetration, misalignment, or porosity, can significantly affect fatigue life. This process is commonly used in precision industrial applications, automotive body assemblies, and aerospace components, where high mechanical strength and clean joint geometry are essential [6].

As illustrated in Figure 5b, a focused laser beam drills a narrow keyhole through the workpiece under shielding gas, producing a deep, low-distortion weld pool beneath the surface. Electron beam welding (EBW) is a related high-energy-density process used mainly for thick aerospace and titanium alloy sections; because it operates in vacuum, EBW produces an even narrower fusion zone and lower distortion than LBW, but weld pool porosity becomes the dominant fatigue crack initiation site in the very-high-cycle regime, distinguishing EBW fatigue behavior from the surface-initiated cracking typical of LBW and arc processes [53]. A further departure from conventional fusion welding is wire arc additive manufacturing (WAAM), in which a welding arc deposits material layer-by-layer to build up a structural component rather than joining pre-formed sheets; because each layer re-heats the ones beneath it, WAAM parts develop a highly anisotropic, columnar-grained microstructure with process-dependent porosity, giving fatigue strength that is markedly lower and more scattered than that of a conventionally welded or wrought material of the same alloy [54].

### 3.3. Gas Metal Arc Welding (GMAW/MIG)

Gas metal arc welding (GMAW) uses a continuously fed welding wire that is supplied into the weld during the welding process, alongside a shielding gas that protects the weld pool [55]. Worldwide, it is among the most versatile and widely used welding processes, owing to its ease of automation and applicability to a wide range of metals. It also has certain limitations; MIG-welded joints commonly exhibit weld toe defects and a relatively wide heat-affected zone, resulting in reduced fatigue strength. Fatigue cracks typically initiate at the weld toe because of geometric discontinuities [11], but the modification of joint profile, by applying post-weld treatment, and monitoring the welding environment can potentially extend the fatigue life of GMAW joints [56]. As depicted in Figure 5c, a continuously fed consumable wire electrode simultaneously sustains the arc and supplies filler metal, shielded by gas flowing through the gun nozzle.

### 3.4. Tungsten Inert Gas Welding (TIG)

Tungsten inert gas (TIG) welding, also known as gas tungsten arc welding, uses a non-consumable tungsten electrode together with an inert shielding gas, typically argon or helium, to produce high-quality welds. The precise control over heat input in TIG welding results in cleaner welds, reduced spatter, and superior surface quality, which improves fatigue performance compared with many conventional arc welding techniques [57]. However, the relatively low welding speed increases manufacturing cost, and weld quality, penetration depth and residual stress all have a strong influence on fatigue life. Applications where weld quality is critical, such as aerospace, nuclear, and precision manufacturing, commonly use TIG welding [58]. As shown in Figure 5d, a non-consumable tungsten electrode sustains the arc under inert gas shielding, while a filler metal is added separately via a hand-fed rod.

### 3.5. Friction Stir Welding (FSW)

Friction stir welding (FSW) is a solid-state joining process that uses the frictional heat generated by a rotating, non-consumable tool to join and plastically deform metal sheets without melting them. Because the material remains below its melting point, FSW significantly reduces defects such as porosity, solidification cracking, and residual stresses that are common in fusion-welding processes [59]. This results in higher fatigue strength and longer life, although fatigue cracks in FSW joints typically initiate in the thermo-mechanically affected zone (TMAZ).

**Figure 6:**
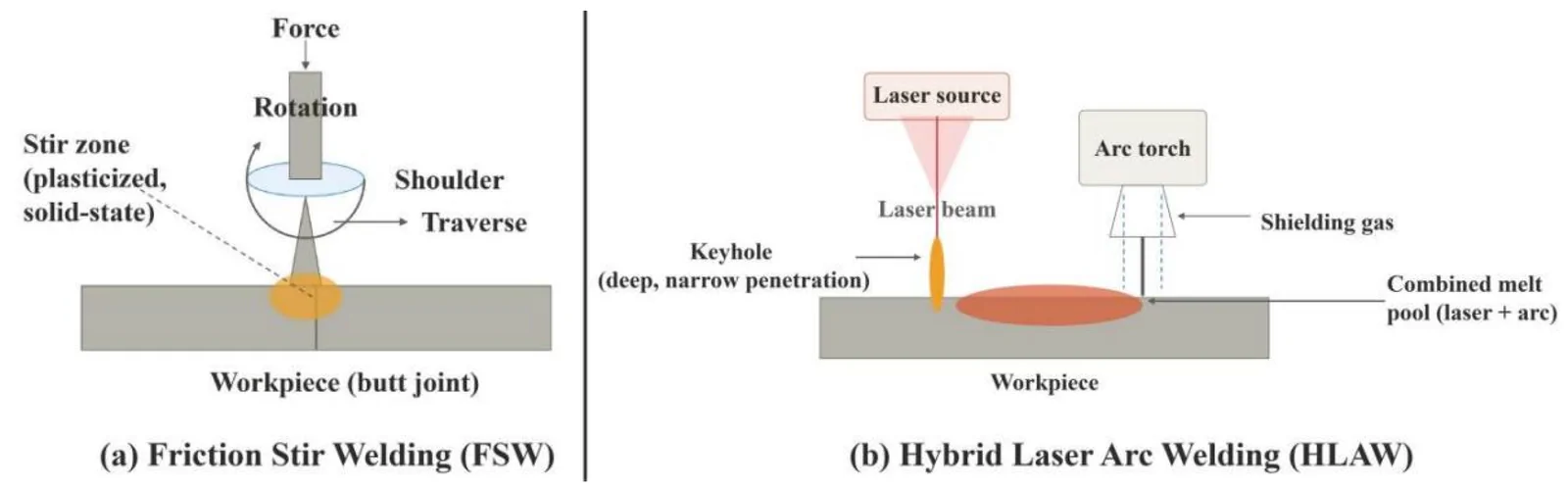
Schematics of the two advanced joining processes reviewed in this section. (**a**) FSW: a rotating, non-consumable tool with a shoulder and pin plunges into the butt joint and traverses along it under downward force; frictional heat plasticizes the surrounding material into a solid-state stir zone without melting it. (**b**) HLAW: a leading laser beam penetrates the workpiece to form a deep, narrow keyhole, while a trailing arc torch under shielding gas melts additional material into the same region, producing a combined melt pool from both heat sources.

FSW is a leading joining process for aluminum alloys in the automotive, railway, marine, and aerospace industries [60]. As shown in Figure 6a, a rotating tool with a shoulder and pin is embedded into the butt joint under axial force and traversed along the seam. Frictional heating alters the structural and mechanical behavior of the material on both sides without reaching its melting point, resulting in a defect-free, solid-state stir zone.

### 3.6. Hybrid Laser Arc Welding (HLAW)

Hybrid laser–arc welding (HLAW) combines the deep-penetration capability of laser beam welding with the gap-bridging ability of arc welding; compared with either process used alone, the HLAW technique offers higher welding speed, greater penetration depth, improved joint quality, and greater tolerance to joint fit-up variations [61].

Welds produced by HLAW typically show improved fatigue resistance, a smaller heat-affected zone, and reduced distortion. However, because poor coordination between the laser and arc processes can introduce defects that adversely affect fatigue performance, careful process optimization is essential. The use of hybrid laser–arc welding is growing in heavy structural fabrication, pipe manufacturing, and shipbuilding [62,63]. As shown in Figure 6b, a leading laser beam creates a deep, narrow keyhole for penetration, while a trailing arc torch, shielded by gas, supplies additional heat and filler material into the same region. The two sources merge into a single combined melt pool, giving higher travel speeds and greater gap tolerance than either process alone.

### 3.7. Comparative Fatigue Performance of Different Welding Techniques

Heat input, welding process, residual stress distribution, defect formation, and weld geometry all significantly affect the fatigue performance of welded metal sheets. Because friction stir welding produces fewer welding defects, it can offer among the highest fatigue strengths of the processes compared here, though this should be read as a general tendency rather than a universal ranking; the actual comparison depends strongly on the alloy and sheet thickness being joined, the specific joint configuration and process parameters used, the loading mode and stress ratio applied, and which failure mechanism governs a given case, so a poorly optimized FSW joint can readily be outperformed by a well-optimized joint made with another process. Laser beam welding also shows excellent fatigue performance because of its narrow weld profile and small heat-affected zone [52]. Hybrid laser–arc welding combines the advantages of both laser and arc welding to achieve higher productivity together with good fatigue resistance. RSW frequently shows the lowest fatigue life among the processes compared here, since stress is concentrated around discrete weld spots, again a tendency rather than an absolute rule, since RSW performance depends heavily on sheet thickness, spot spacing and diameter, and especially on loading mode; RSW joints loaded predominantly in shear behave quite differently from those loaded in peel or cross-tension, and the ranking can shift accordingly, while TIG welding often provides better fatigue performance than MIG welding owing to lower defect levels, again subject to the same process- and application-specific caveats. The choice of welding process must therefore balance fatigue performance, productivity, material type, and application requirements [64]. These trends are consolidated in Table 5, which ranks the six welding processes reviewed in Section 3.1, Section 3.2, Section 3.3, Section 3.4, Section 3.5 and Section 3.6 by relative fatigue strength, dominant defect or failure mode, typical crack initiation site, and typical applicable sheet thickness range, with the process-specific literature underpinning each ranking listed in its References column; the ranking should be read as indicative of typical, reasonably well-controlled process conditions rather than as a fixed hierarchy, since alloy, thickness, joint configuration, process parameter optimization, loading mode, stress ratio, and the governing failure mechanism can all shift a given process up or down this ordering for a specific application.

### 3.8. Dissimilar Material and Hybrid Joining Technologies

Lightweighting strategies increasingly require the joining of dissimilar metal sheets, such as steel-to-aluminum or aluminum-to-magnesium combinations, which cannot be reliably fusion-welded because of intermetallic compound formation and incompatible melting points. Friction stir welding and friction stir spot welding have therefore become key enabling processes for dissimilar-material joints, since the solid-state mixing action limits the thickness of brittle intermetallic layers and allows a mechanically interlocked, metallurgically bonded joint to form even between metals with very different thermophysical properties [66]. The fatigue performance of these joints is highly sensitive to process parameters; tool rotational and travel speed control heat input, the degree of material mixing, and the resulting grain structure in the stir zone and thermo-mechanically affected zone, with excessive heat input coarsening the fatigue-limiting stir-zone grain structure and insufficient heat input leaving unbounded interfacial regions that act as pre-existing cracks [67]. Comparative studies between friction stir welding and conventional MIG/TIG arc welding have shown that, for equivalent aluminum alloys, the solid-state process consistently produces higher fatigue strength because it avoids the porosity, solidification cracking, and coarse columnar grain structure associated with arc-welded fusion zones [68].

Beyond friction-based processes, adhesive–weld hybrid joints and weld-bonding techniques, which combine a structural adhesive layer with spot or laser welds, are gaining industrial attention because the adhesive bridges the gap between discrete weld points and provides a secondary load path if a weld nugget fails, hence giving a further margin of fatigue safety in multi-material automotive structures [15]. Wei et al. reviewed the design, manufacturing, and modeling of adhesively bonded joints, and showed that their strength and durability depend strongly on adhesive selection, surface preparation, and defect detection, considerations that carry over directly to the adhesive layer used in weld-bonded hybrid joints [69].

As a non-fusion mechanical fastening alternative to both fusion welding and adhesive bonding, self-piercing riveting (SPR) is increasingly used for dissimilar-material and multi-material sheet assemblies where fusion welding is impractical. Because load transfer occurs mechanically through the interlocked rivet rather than through a fused or adhesive interface, SPR joints avoid the heat-affected zone-related fatigue degradation intrinsic to welding altogether; their fatigue performance is instead governed by rivet hole stress concentration and interlocking geometry, rather than by weld toe or nugget geometry, giving SPR a fundamentally different fatigue-critical failure mode from the welded and weld-bonded joints discussed above. This positions solid-state welding, weld bonding, and mechanical fastening as complementary rather than competing joining strategies, to be selected according to which fatigue-critical failure mode is most tolerable for a given material combination and application; a comparison that is revisited from a sustainability perspective in Section 8.2.4, where these joining methods are benchmarked directly against one another.

## 4. Factors Affecting Fatigue Life of Welded Metal Sheets

**Figure 7:**
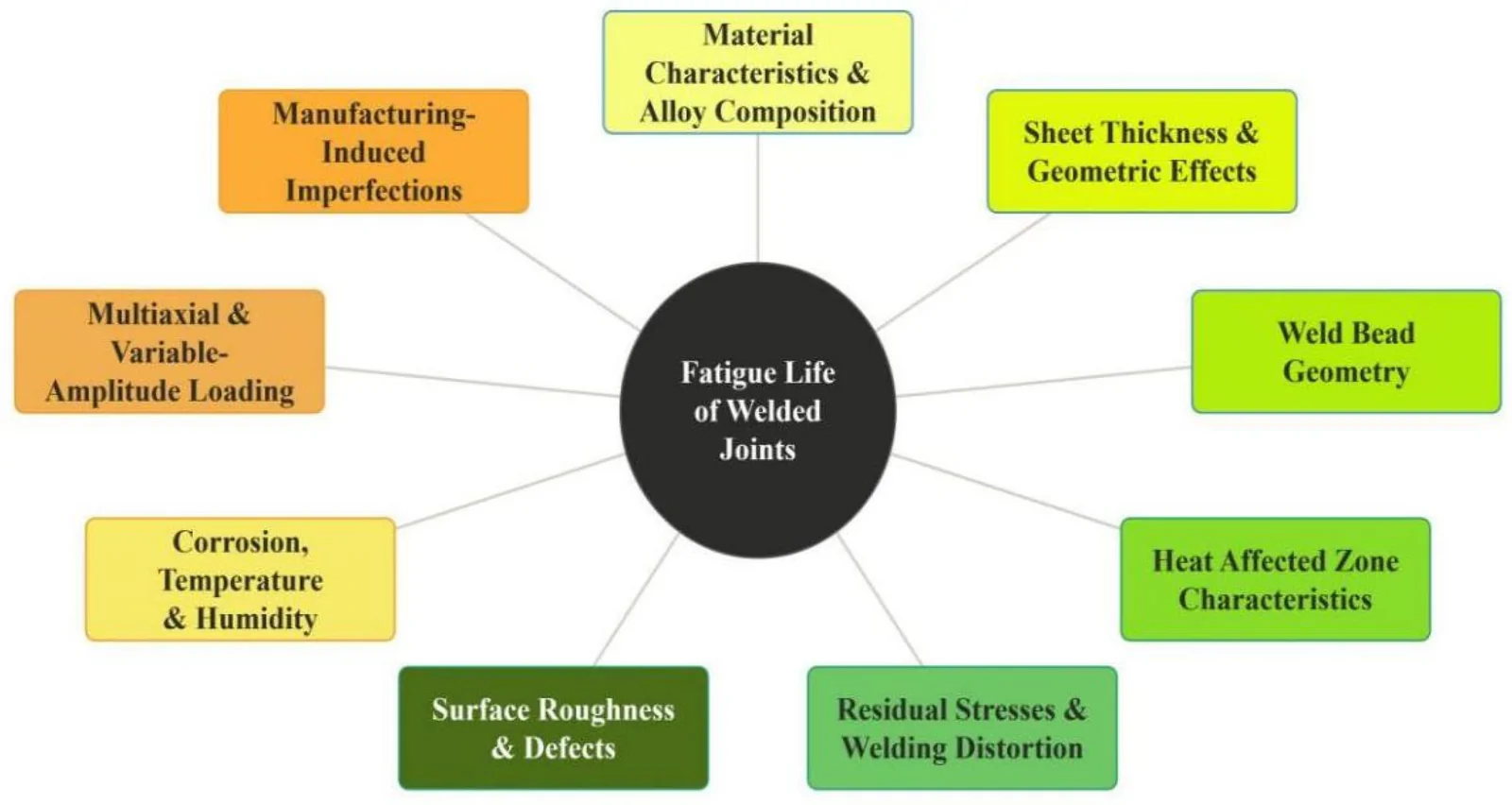
Key factors affecting fatigue life of welded metal sheets under service conditions.

The fatigue performance of a welded metal sheet is never governed by a single variable; rather, it emerges from the combined effect of material, geometric, process, and environmental factors that interact throughout the component’s service lifespan [15]. Whereas Section 3.7 compared fatigue performance across specific welding processes, this section examines the underlying material, geometric, and environmental factors in isolation from any particular process, providing the mechanistic basis for the process-level comparisons drawn in Section 3.7. Figure 7 organizes these influences into the ten themes examined in this section, from base material composition and sheet thickness through weld bead geometry, the heat-affected zone, residual stress, surface condition, and environmental exposure, to loading complexity, manufacturing defects, and weld sequence control, providing a roadmap for the discussion that follows.

### 4.1. Material Characteristics and Alloy Composition

The mechanical properties and chemical composition of the base material have a substantial effect on the fatigue life of welded metal sheets. Resistance to fatigue crack initiation and propagation is governed by properties such as tensile strength, yield strength, stiffness, ductility, fracture toughness, and grain size. Alloying elements such as carbon, manganese, molybdenum, and nickel increase strength and corrosion resistance, but can also influence fatigue behavior and weldability [70]. When appropriate welding procedures are used, aluminum alloys and high-strength steels can achieve good fatigue performance; however, excessive hardness or brittle microstructures formed during welding may reduce fatigue life. Selecting a material with stable mechanical properties suited to the welding process is therefore essential for improving the durability of welded assemblies [71].

### 4.2. Sheet Thickness and Geometric Effects

The fatigue strength of welded joints is strongly influenced by sheet thickness, as thinner sheets generally experience lower residual stresses and better load redistribution, but they can also be more vulnerable to local stress concentration [72]. Geometric features that increase local stresses and promote fatigue crack initiation are holes, sharp corners, abrupt thickness changes, and poor joint design, and inappropriate geometry can substantially reduce fatigue life in welded structures [73]. Optimizing sheet thickness and geometry improves fatigue resistance by distributing stress more evenly. Design standards have traditionally penalized fatigue strength for plates thicker than a reference thickness while largely disregarding the converse “thinness effect,” by which thinner-than-reference welded sheets can exhibit increased fatigue strength; a recent combined experimental and probabilistic study of butt-welded and non-load-carrying cruciform joints under constant-amplitude loading used the effective notch stress method together with a probabilistic framework to quantify this thinness effect and to propose refined, statistically grounded size-effect corrections for thin welded sheet sections [74].

### 4.3. Weld Bead Geometry

The fatigue performance of welded metal sheets is directly affected by weld bead geometry, and the magnitude of stress concentration near the weld is governed by many factors including the weld profile, penetration depth, bead size, toe angle, and weld reinforcement [75]. Localized stress concentrations caused by sharp weld toes and excessive reinforcement accelerate fatigue crack initiation, whereas smooth bead profiles and gradual weld transitions reduce stress concentration [76]. Post-weld treatment methods such as toe grinding, TIG dressing, and weld toe remelting are commonly used to improve weld profile or to increase fatigue resistance [77].

### 4.4. Heat-Affected Zone (HAZ) Characteristics

The heat-affected zone (HAZ) is the region close to the weld that undergoes thermal cycling without melting. This process can change the material’s microstructure, hardness, grain size, and mechanical properties [78]. Depending on the welding process and the associated heat input, the HAZ can become either softened or hardened, ultimately affecting its fatigue behavior. Fatigue crack initiation is more likely in HAZ regions with reduced strength, and controlling heat input and cooling rate during welding can minimize unwanted microstructural changes and thereby improve fatigue performance [79].

### 4.5. Residual Stresses and Welding Distortion

The non-uniform heating and cooling of welded components generate residual stresses during the welding process. Tensile residual stresses are particularly disadvantageous because they accelerate fatigue crack propagation by increasing the effective stress intensity [23]. Thermal expansion and load-induced welding distortion can also alter the load path and add further strain to the structure. A range of stress-relief methods, including cold working, heat treatment, and vibratory stress relief, are used to reduce residual stresses and extend fatigue life [80]. Welding sequence can also be carefully controlled to reduce distortion and improve structural stability [81].

The magnitude of the heat input used during welding compounds these effects by governing the heat-affected zone microstructure that residual stresses act upon. Comparing multi-pass submerged-arc welds made with roughly 15 and 50 kJ/cm heat input in a heavy-gauge FH690 marine steel, Bai et al. showed that the coarse-grained heat-affected zone, rather than the weld metal or base material, controlled fatigue life, and that the mixed granular- and lath-bainite microstructure produced by the higher heat input gave a lower fatigue strength, so heat-input control during welding procedure qualification is itself a fatigue-relevant design variable [78].

### 4.6. Surface Roughness and Defects

Because fatigue cracks typically initiate from surface irregularities, surface quality has a significant influence on fatigue crack initiation. These surface irregularities that accelerate fatigue failure include rough surfaces, weld spatter, undercuts, porosity, slag inclusions, lack of fusion, and micro cracks [82]. High-quality welding and improved surface finishing reduce stress concentration and delay crack initiation. Some non-destructive testing methods, including ultrasonic testing, radiographic inspection, magnetic particle testing, and dye-penetrant testing, are routinely used to detect surface and internal defects before a component enters service [83].

### 4.7. Effect of Corrosion, Temperature, and Humidity

Environmental conditions are among the leading causes affecting the fatigue strength of welded metal sheets, as extreme heat and harsh environments can reduce material strength and increase creep deformation, which accelerates crack initiation and growth [53]. When such structures are exposed to marine environments, salt and moisture cause corrosion and localized deterioration; when corrosion and cyclic loading act together, fatigue life is typically much shorter than under mechanical stress alone. Protective coatings made of corrosion-resistant materials, together with regular maintenance, are among the most widely used methods to improve fatigue resistance [84]. In offshore wind and marine applications specifically, Adedipe et al. showed that seawater exposure alters both short- and long-crack growth rates in the heat-affected zone of S355 steel monopile weldments, and that welding-induced microstructural changes in the heat-affected zone can introduce crack branching that is not observed in the parent material [25].

### 4.8. Multiaxial and Variable-Amplitude Loading

In real-world production applications, welded structures rarely experience simple uniaxial loading; instead, the combined effects of variable-amplitude loading and multiaxial stresses resulting from fluctuating operating conditions are far more common [85]. These complex loading conditions change the stress distribution, accelerate the accumulation of fatigue damage, and make prediction much more difficult. Load sequence effects, such as overloads and underloads, also affect crack growth rates, and are not always well predicted by simple linear fatigue methods; advanced multiaxial fatigue criteria and cumulative damage models are therefore used to improve fatigue life assessment under realistic service conditions [86].

### 4.9. Manufacturing-Induced Imperfections

The fatigue performance of welded metal sheets is also affected by defects introduced during manufacturing. Some of the most common defects include incomplete penetration, undercut, porosity, excessive reinforcement, misaligned welds, inclusions, and dimensional errors [24]. These defects compromise structural stability by creating localized stress concentrations that promote early fatigue crack initiation. Non-destructive testing, process monitoring, optimized welding procedures, and strict quality control are used to reduce this risk and improve fatigue reliability. Automated manufacturing and modern robotic welding equipment have further reduced the occurrence of such defects, resulting in more reliable fatigue performance [87]. Having discussed each factor individually across Section 4.1, Section 4.2, Section 4.3, Section 4.4, Section 4.5, Section 4.6, Section 4.7, Section 4.8 and Section 4.9, Table 6 now ranks them by relative impact, confirming that weld bead geometry, tensile residual stress, surface or weld defects, and loading spectrum exert the strongest influence on fatigue life, ahead of the heat-affected zone, base material properties, and geometric discontinuities, with environmental exposure and sheet thickness playing a comparatively more moderate role.

The ranking in Table 6 confirms, in a quantitative form, the qualitative discussion presented across Section 2.4 and Section 4.1, Section 4.2, Section 4.3, Section 4.4, Section 4.5, Section 4.6, Section 4.7, Section 4.8 and Section 4.9, showing that weld bead geometry, residual stress, surface or weld defects, and loading spectrum exert the strongest influence on fatigue life, and should therefore be prioritized in both design and quality control efforts, ahead of base material selection and sheet thickness alone.

### 4.10. Weld Sequence, Fixturing, and Residual Stress Control

The order in which individual weld passes and seams are deposited, together with the rigidity of the fixtures used to hold sheet components during welding, has a first-order influence on the final residual stress distribution and distortion pattern in a welded sheet assembly [89].

Because residual stress fields are a combination of stress contributions from each successive pass, changing the weld sequence redistributes, rather than eliminates, the residual stress. Poorly chosen sequences can concentrate tensile residual stress precisely at the locations of highest applied cyclic stress, compounding the resulting fatigue penalty [90]. Some practical strategies include back-step and skip welding sequences that balance thermal contraction, and symmetric fixturing that restrains out-of-plane distortion without over-constraining the joint; however, post-weld stress relief is generally the most effective treatment. Quantifying the resulting residual stress field remains an active research problem, since the magnitude and sign of residual stress directly govern the effective stress ratio experienced by a fatigue crack and can shift the joint into either the crack-accelerating or crack-retarding regime described in Section 5.3 [74,91].

## 5. Numerical Simulation and Multi-Physics Modeling

**Figure 8:**
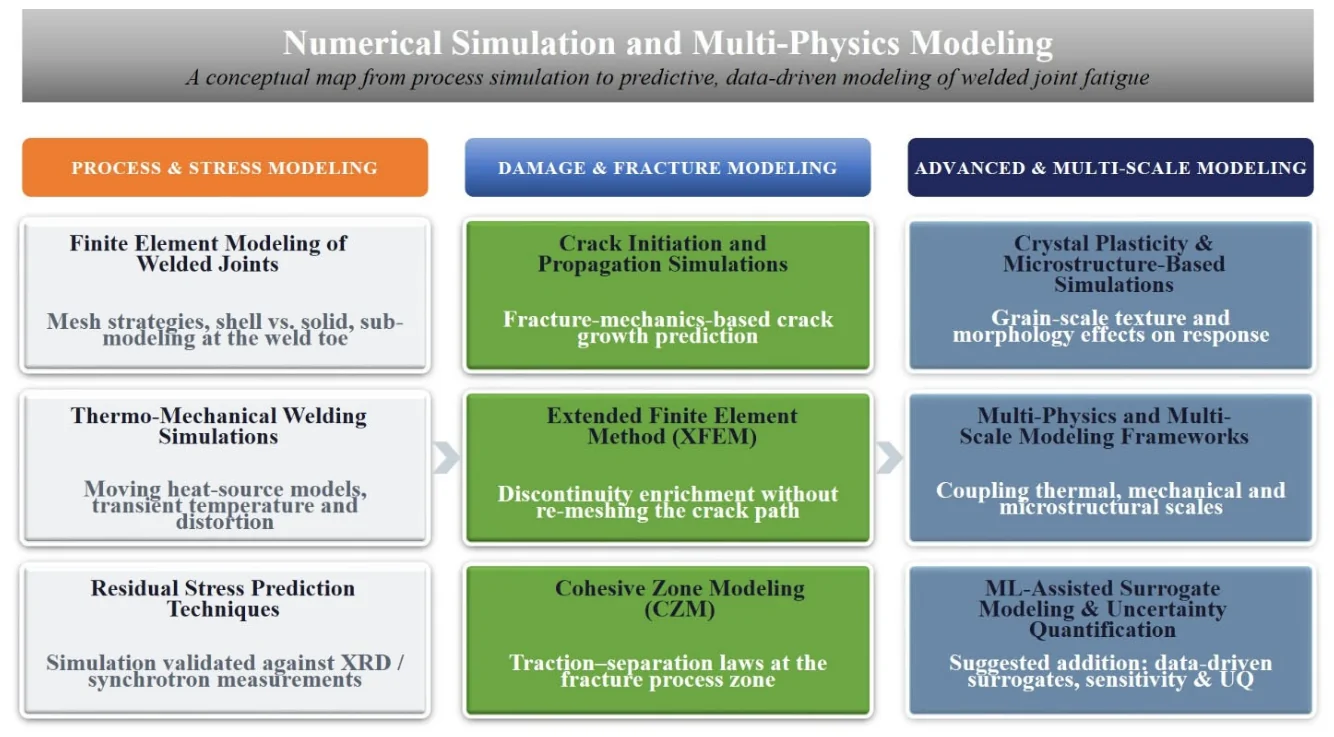
Conceptual map of numerical simulation and multi-physics modeling approaches for welded joint fatigue, from process and stress modeling (finite element modeling of welded joints, thermo-mechanical welding simulation, residual stress prediction) through damage and fracture modeling (crack initiation and propagation simulation, the extended finite element method, cohesive zone modeling) to advanced and multi-scale modeling (crystal plasticity and microstructure-based simulation, multi-physics/multi-scale frameworks, and machine learning-assisted surrogate modeling and uncertainty quantification) [92].

Beyond the empirical and standards-based descriptions introduced so far, computational simulation offers a route to predicting fatigue behavior directly from first principles. Figure 8 maps the landscape of numerical and multi-physics techniques covered in this section, organized into three progressively more advanced tiers, namely, process and stress modeling, damage and fracture modeling, and advanced multi-scale modeling, culminating in the machine learning-assisted surrogate approaches.

### 5.1. Finite Element Modeling of Welded Joints

The finite element method (FEM) is one of the most widely used numerical approaches for analyzing the fatigue performance and mechanical behavior of welded joints. By discretizing a welded structure into small finite elements, FEM can accurately predict stress distribution, deformation, and stress concentration under various loading conditions. It is particularly useful for complex weld geometries where analytical solutions are difficult to obtain [93]. FEM is routinely used in fatigue analysis to identify critical locations for crack initiation, estimate stress concentration factors, and predict fatigue life using stress life or strain life methods. Mesh quality, material models, boundary conditions, and the representation of weld geometry all affect the accuracy of finite element predictions [92,94].

### 5.2. Thermo-Mechanical Welding Simulation

Thermo-mechanical welding simulations model the coupled thermal and mechanical processes that occur during welding. These models predict temperature distribution, thermal cycles, cooling rates, phase transformations, residual stresses, and welding distortion [95]. The transient temperature field is first determined through heat transfer analysis, which is then coupled with structural analysis to calculate thermal expansion and contraction. Such simulations support the optimization of welding parameters, minimize distortion, and improve fatigue performance. ANSYS and ABAQUS are among the finite element software packages commonly used for the thermo-mechanical analysis of welded structures [96,97].

### 5.3. Residual Stress Prediction Techniques

Since tensile residual stresses accelerate fatigue crack growth, the residual stresses generated during welding have a major effect on fatigue life. In this context, numerical and simplified analytical models, such as thermo-elastic–plastic finite element analysis and inherent strain methods, are commonly used to estimate residual stresses [98]. Using simulations of heating and cooling sequences that occur during welding, these approaches predict the resulting stress field, which is experimentally verified by X-ray diffraction (XRD), neutron diffraction, hole-drilling, and contour methods. Hence, accurate residual stress predictions allow engineers to optimize welding procedures and improve structural durability [99].

These experimental techniques are not interchangeable, and choosing among them involves a genuine trade-off between measurement depth, spatial resolution, cost, and how destructive the technique is to the component being measured, so it is worth comparing them explicitly rather than treating them as a single undifferentiated verification step. X-ray diffraction (XRD) is non-destructive and widely accessible using laboratory-scale equipment, but it is fundamentally a surface-sensitive technique, penetrating only tens of micrometers into most structural metals, so it characterizes near-surface residual stress well but cannot resolve how that stress varies through the plate or weld thickness. Neutron diffraction extends the same diffraction principle to bulk through-thickness measurement, penetrating centimeters into steel and aluminum alike, but this capability requires access to a reactor or spallation-source neutron facility, making it far less accessible for routine industrial use, and typically limiting it to a small number of validation specimens rather than production-scale quality control. High-energy (synchrotron) X-ray diffraction occupies an intermediate position; it achieves bulk penetration depths comparable to neutron diffraction with substantially faster data acquisition and finer spatial resolution, but likewise depends on synchrotron beamtime access, which is scarce and typically allocated competitively. Hole-drilling is semi-destructive—a small hole (typically 1–2 mm deep) is drilled at the measurement point while surrounding strain gauges record the relieved strain—and is comparatively inexpensive and field-deployable under ASTM E837, but it is limited to near-surface stress and leaves a small permanent hole in the component. The contour method, by contrast, is fully destructive; the component is cut in two using precision wire electrical discharge machining, and the resulting out-of-plane deformation of the cut surface is measured and used to back-calculate the original stress field, yielding a full two-dimensional cross-sectional stress map rather than a single-point value, at the cost of destroying the specimen entirely. Numerical prediction and experimental measurement are consequently best understood as complementary rather than as a one-way validation step; an accurate numerical prediction lets a full-field residual stress estimate be obtained on every component of interest, including for the more complex, multi-pass weld geometries where residual stress accumulation from each successive pass is difficult to predict analytically, and where dedicated equivalent-pass modeling strategies have been developed to keep simulation cost tractable without sacrificing accuracy [100], while a comparatively sparse set of point or cross-sectional experimental measurements, drawn from the techniques compared above, is used to check that prediction against reality, as demonstrated for dissimilar steel pipe joints where finite-element-predicted residual stresses were verified directly against physical measurements taken at multiple points across the weld [101].

**Figure 9:**
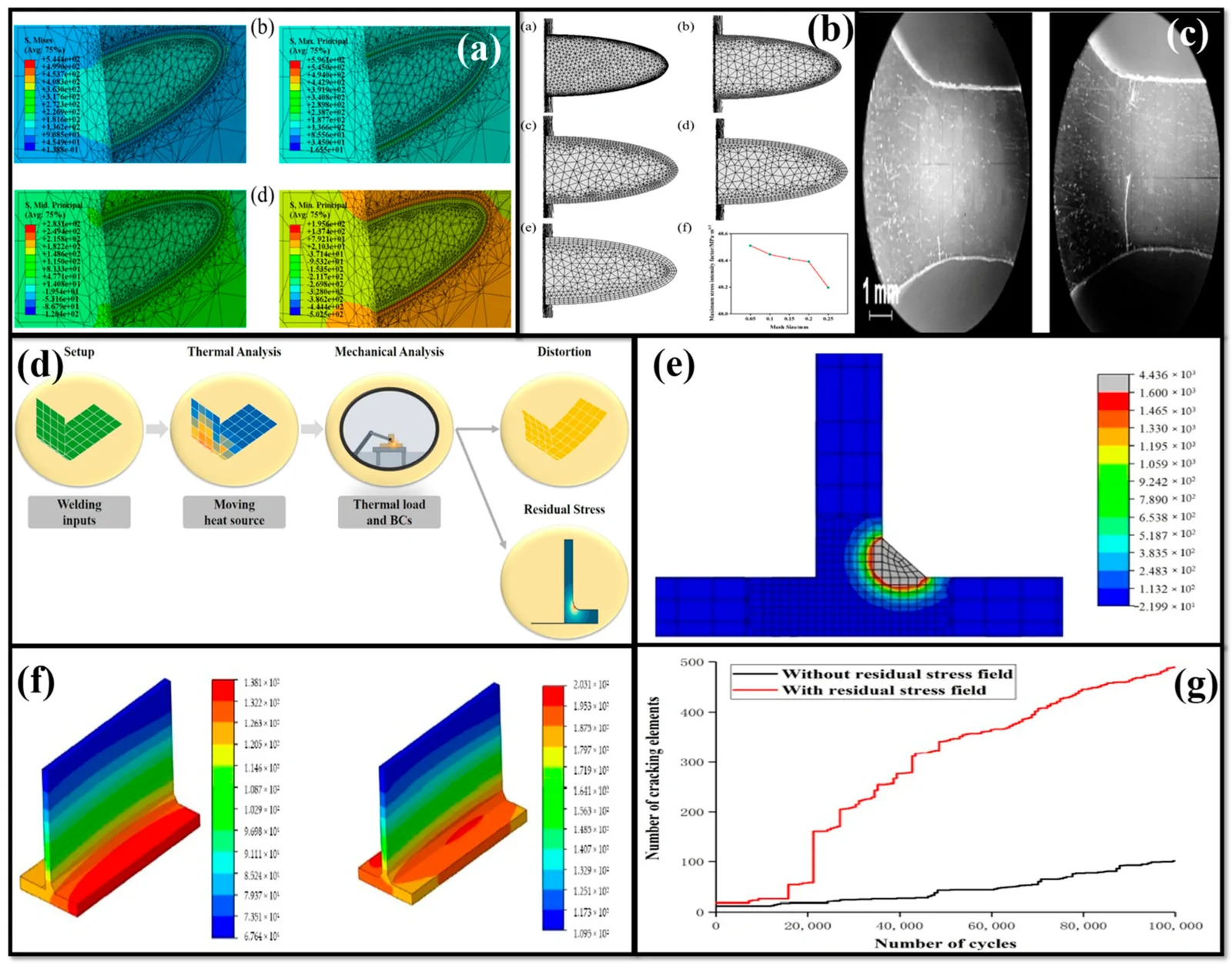
Finite element analysis of stress concentration, welding simulation, and residual stress effects in welded joints, (**a**) mises, maximum, middle, and minimum principal stress contours at the buried weld defect crack tip on the refined FE mesh (adapted from [105] under CC-BY), (**b**) crack tip mesh convergence study (adapted from [105] under CC-BY), (**c**) stages of crack development in a specimen (adapted from [106] under CC-BY), (**d**) schematic of the sequentially coupled thermo-mechanical welding simulation workflow, (**e**) finite element heat transition/stress contour result at a fillet weld (adapted from [107] under CC-BY), (**f**) comparison of predicted stress distributions for unstiffened and stiffened weld details (adapted from [107] under CC-BY), (**g**) predicted number of cracking elements versus loading cycles with and without an incorporated residual stress field, showing markedly accelerated damage accumulation when residual stress is included (adapted from [107] under CC-BY).

Predicting the as-welded residual stress field is, however, only a starting point, and three further aspects of residual stress behavior are directly relevant to fatigue life prediction but are addressed less consistently in the numerical studies discussed above than the initial prediction step itself. First, residual stresses are not permanent under service loading: where the applied stress superimposed on the local residual stress exceeds the material’s cyclic yield strength, local plastic shakedown progressively relaxes the initial residual stress magnitude over the first cycles of loading, so that an as-welded residual stress field, however accurately predicted by thermo-elastic-plastic or inherent strain simulation, can substantially overestimate the residual stress actually acting for most of the component’s service life. Second, as a fatigue crack advances through a welded joint, the residual stress field ahead of and around the crack tip redistributes as material carrying part of the original field is separated by the advancing crack and the remaining ligament re-equilibrates; this means the residual stress-driven acceleration in crack growth illustrated in Figure 9g cannot be treated as a fixed offset applied uniformly across the full crack growth history, but should in principle be recomputed, or at least bounded, as the crack advances. Third, residual stress interacts with the applied mean stress in a way that is functionally equivalent to a local shift in stress ratio R: a tensile residual stress effectively raises the local mean stress and can promote crack-tip opening even under a nominally low-R or compressive applied cycle, whereas a compressive residual stress can suppress crack growth by keeping the crack closed over part of the applied cycle. Capturing this interaction correctly generally requires superposing the (evolving) residual stress intensity factor onto the applied-load stress intensity factor within a crack closure or effective stress intensity range framework, rather than treating residual stress and applied mean stress as independent, simply additive inputs. Collectively, cyclic relaxation, crack-driven redistribution, and mean stress interaction mean that a single as-welded residual stress prediction is best regarded as an initial condition for fatigue life assessment, rather than a fixed input carried unchanged through the component’s service life; explicitly modeling this evolution, rather than assuming a static residual stress field, remains one of the more demanding requirements identified among the research gaps in Section 9.3.

### 5.4. Crack Initiation and Propagation Simulation

Damage evolution in welded structures under cyclic loading can be well understood through the numerical simulation of fatigue crack initiation and propagation. Crack propagation is typically modeled using fracture mechanics concepts based on the stress intensity factor, while crack initiation is more often predicted using local stress or strain criteria [102]. Although more sophisticated models can account for residual stress, variable-amplitude loading, and crack closure effects, Paris’ law remains widely used for estimating stable crack growth rates. In safety-critical applications, these models support maintenance planning by estimating the remaining useful life of welded components [103,104]. Figure 9a,b present the finite element stress analysis stage, where Figure 9a shows four abaqus contour plots (Mises, and maximum/mid/minimum principal stresses) mapped onto a refined crack tip mesh at a buried weld defect, and Figure 9b demonstrates a mesh convergence study across six element densities together with a stress intensity factor versus mesh size plot used to justify the selected mesh resolution [105]. Figure 9c shows experimental validation, with two transmitted-light photographs of an elliptical specimen cross-section (1 mm scale) capturing the physical fatigue crack front, conceptually paralleling the crack growth [106]. The thermo-mechanical welding simulation workflow used to predict distortion and residual stress is presented in Figure 9d. The direct consequence of residual stress for crack growth prediction (Figure 9e–g) is shown in panel (g), which plots the number of cracking elements against load cycles from 0 to 100,000 (x-axis) against 0 to roughly 500 cracking elements (y-axis); the residual stress-included curve (red) reaches approximately 490 cracking elements by 100,000 cycles, compared with approximately 100 cracking elements for the residual stress-free curve (black) at the same cycle count, a difference of roughly fivefold [107]. The source paper additionally reports that this acceleration factor is load-dependent, ranging from approximately twofold at a lower applied displacement to several-fold or more at higher displacements, so the specific ratio shown in panel (g) should be read as one point on that load-dependent trend rather than as a single universal multiplier.

### 5.5. Extended Finite Element Method (XFEM)

The extended finite element method (XFEM) is a refined numerical technique that allows cracks to initiate and propagate independently of the underlying finite element mesh. Unlike conventional FEM, XFEM does not require remeshing as the crack grows, which significantly reduces computational cost while maintaining simulation accuracy [108]. XFEM introduces enrichment functions into the standard finite element formulation to represent discontinuities; because of this, it can accurately model complex crack paths and crack propagation even in heterogeneous materials, and is now increasingly used in the fatigue analysis of welded joints [109].

### 5.6. Cohesive Zone Modeling (CZM)

A common fracture mechanics approach is cohesive zone modeling (CZM), which simulates crack initiation and propagation along potential crack paths. This approach uses traction separation laws that describe the progressive separation of material, rather than treating cracks as pre-existing discontinuities in structures [5]. The outcomes of this approach can directly capture damage evolution, resistance to crack propagation, and interfacial failure between joined materials. Cohesive zone models are useful for predicting fatigue damage in welded joints and in composite materials where significant interfacial failure is present [110].

### 5.7. Crystal Plasticity and Microstructure-Based Simulation

Crystal plasticity modeling characterizes the deformation behavior of individual grains within polycrystalline materials, and also allows the prediction of fatigue crack opening at the microstructural scale [111]. These simulations capture local stress concentrations, crystallographic slip systems, and grain boundary orientation, none of which can be obtained by conventional continuum models. They also reveal how phase distribution, texture, and grain size influence fatigue behavior. These tools are used to develop lightweight alloys and advanced high-strength steels capable of showing improved fatigue resistance [111].

### 5.8. Multi-Physics and Multi-Scale Modeling Frameworks

**Figure 10:**
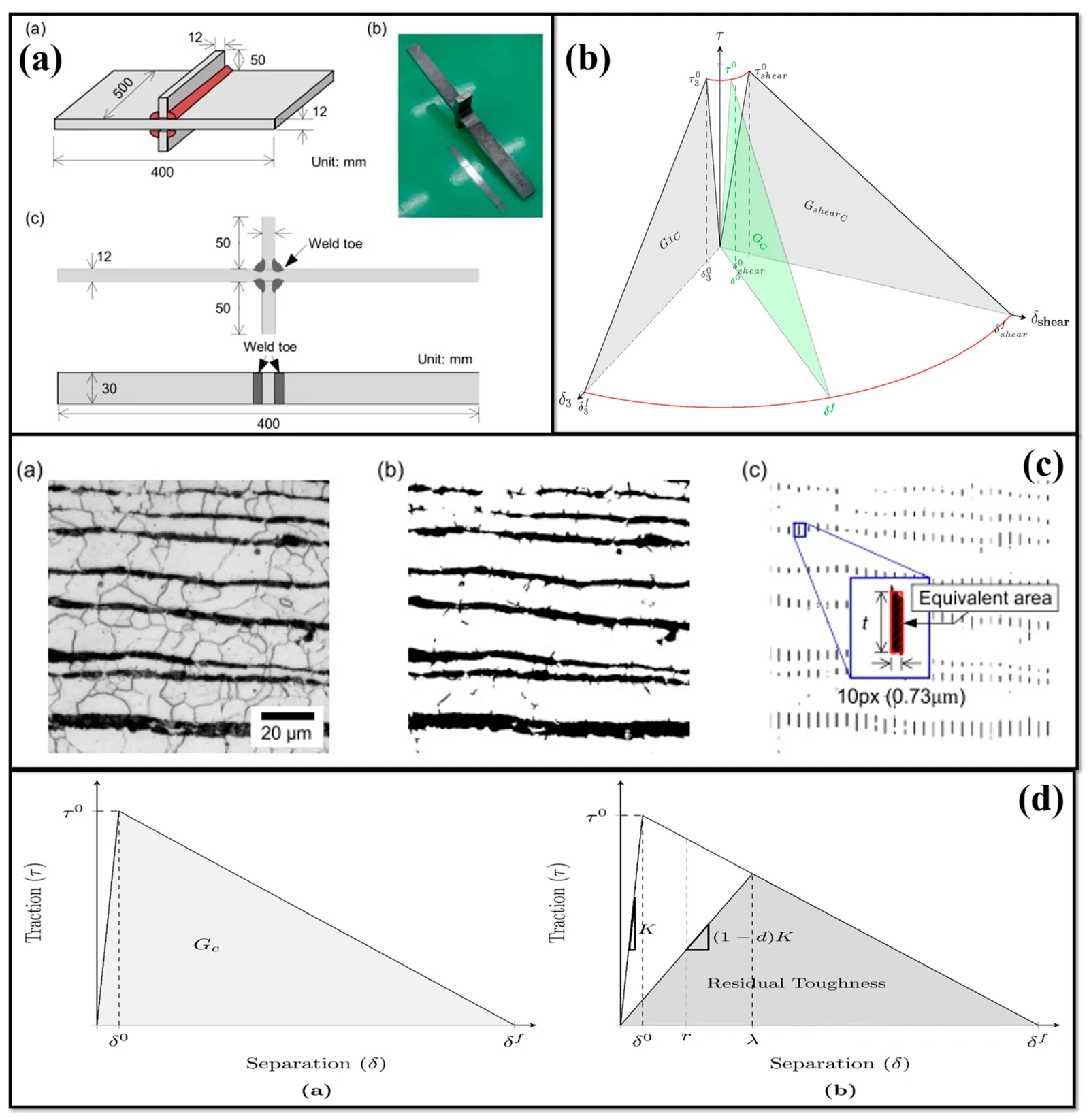
Cohesive-zone and multi-scale representation of fracture in welded joints, (**a**) cruciform welded specimen geometry and test coupon, with weld toe fracture locations indicated [113], (**b**) mixed-mode cohesive zone fracture envelope construction showing mode-I and mode-II (shear) traction–separation surfaces and the corresponding critical fracture energies (adapted from [12] under CC-BY), (**c**) micrographs of the banded weld/HAZ microstructure at the fracture process zone scale, together with the equivalent area idealization used to map microstructural bands onto cohesive elements [113], (**d**) bilinear traction–separation laws for an undamaged interface (**left**) and a degraded interface exhibiting reduced residual toughness after prior damage (**right**) (adapted from [12] under CC-BY).

Multi-physics and multi-scale modeling integrates different physical phenomena occurring at different length scales within a single computational framework. These models are especially designed to simulate the entire welding process and also to predict long-term structural performance by combining thermal, mechanical, and fatigue analyses [95]. Grain-level deformation is captured by micro-scale simulations, weld microstructure evolution is captured by meso-scale models, and structural stresses and fatigue life are evaluated by macro-scale models. The combination of finite element analysis, computational fluid dynamics (CFD), fracture mechanics, and materials science makes fatigue-life prediction for welded metal sheets far more accurate. These integrated frameworks underpin smart-manufacturing engineering systems and digital twin technologies [112]. Figure 10 grounds these multi-scale ideas in a concrete cohesive zone example; it shows the cruciform test geometry and fracture locations (Figure 10a) [113], the mixed-mode fracture envelope that couples the mode-I and mode-II cohesive energies (Figure 10b) [12], the banded weld/HAZ microstructure at the fracture process zone scale (Figure 10c) [114], and the resulting bilinear traction–separation laws for an undamaged interface versus one with reduced residual toughness after prior damage (Figure 10d) [12].

### 5.9. Machine Learning-Assisted Numerical and Surrogate Modeling

Because high-fidelity finite element, XFEM, or crystal-plasticity simulations of welded joints are computationally expensive, there is growing interest in coupling numerical simulation with machine learning to build fast surrogate models that reproduce simulation accuracy at a fraction of the computational cost [115]. Hybrid physics-informed and data-driven frameworks combine the interpretability and physical consistency of classical fatigue models with the pattern recognition capability of machine learning, using simulation or experimental data to correct systematic biases in simplified physical models rather than replacing the physics altogether [115]. Explainable machine learning models, including gradient-boosted trees and random forests trained with feature importance analysis, have been used to predict fatigue failure in small-scale butt-welded joints while retaining insight into which geometric and process variables drive the prediction, addressing a key criticism of black box neural network approaches [116]. Model reduction techniques that train a machine learning metamodel on a library of finite element and fatigue analysis results allow the geometric characteristics, loading conditions, and weld class of a joint to be mapped directly onto a predicted fatigue life, dramatically reducing the number of full simulations needed during design iteration [117]. Similar approaches have been applied to predict crack depth and remaining fatigue life in orthotropic steel-deck U-rib welded joints using support vector regression, Gaussian process regression, and ensemble methods [118], and to the fatigue-life estimation of welded joints in dissimilar alloys using bio-inspired optimization algorithms such as artificial bee colony intelligence [119], building on a broader body of work reviewing soft-computing methods for fatigue-life estimation. Collectively, these studies indicate that surrogate and hybrid models are best viewed as accelerators and refiners of established physical fatigue models rather than as replacements for them, particularly given the comparatively small, expensive-to-generate fatigue datasets that are typical of welded-joint research. The physics-informed neural network (PINN) is a newer approach that embeds established fatigue physics relationships into the network, enabling the model to make reasonable predictions even when only a small amount of data is available. In orthotropic steel decks where deck rib double side welded joints are used, a modified PINN has been developed for improved fatigue life prediction. This model uses an activation function that corresponds to a physical mode and also follows physical rules in the loss function, while considering the effect of deck thickness based on observed data. As a result, the predictive capability of this PINN has been improved compared with conventional artificial neural networks and standard PINN models [120]. Such physics-constrained architectures are particularly attractive for welded joint fatigue prediction, where high-quality experimental datasets are limited, and purely data-driven models risk extrapolating outside their training domain.

### 5.10. Comparative Assessment of Numerical and Multi-Scale Modeling Approaches

The numerical and multi-scale methods introduced in Section 5.5, Section 5.6, Section 5.7 and Section 5.8 differ substantially in the input data they require, their computational cost, and the length scale at which they are best applied, which in turn determines which method is appropriate for a given fatigue assessment task rather than simply which is most advanced. Table 7 consolidates this comparison for XFEM, cohesive zone modeling, crystal plasticity, and multi-physics/multi-scale frameworks, following the same input–cost–applicability–limitation structure used for the conventional methods in Table 8, together with the governing literature for each method in its References column. Read together with Table 8, the two tables position the numerical/multi-scale methods as complementary to, rather than a replacement for, the conventional S-N, strain life, and fracture mechanics approaches; the numerical methods trade higher computational cost and more demanding input data for the ability to resolve crack path detail, interfacial behavior, or microstructural responses that the conventional methods necessarily treat only empirically.

## 6. Conventional Fatigue Life Prediction Approaches

**Figure 11:**
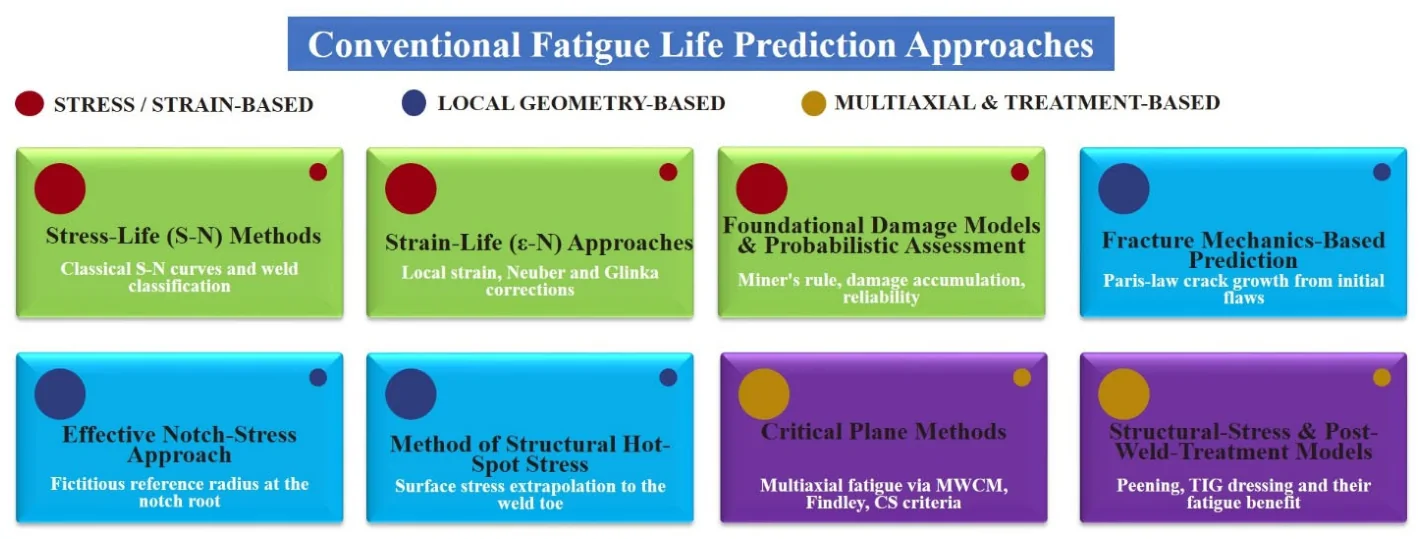
Overview of conventional fatigue life prediction approaches for welded joints, including stress/strain, local geometry-based methods, fracture mechanics-based prediction, multiaxial and post-weld treatment-based methods.

Having examined in Section 5 how numerical simulation captures the fatigue behavior of welded sheet structures, this section turns to the simplified, code- and standards-based prediction methods used in routine engineering design. Readers primarily interested in established design-code methods may nonetheless read Section 6 on its own, since it does not depend on the simulation content presented here. Figure 11 classifies the eight conventional approaches covered in Section 6.1, Section 6.2, Section 6.3, Section 6.4, Section 6.5, Section 6.6, Section 6.7, Section 6.8 and Section 6.9 into three families—stress/strain-based methods, local geometry-based methods, and multiaxial/treatment-based methods—providing a map of the method landscape before each approach is examined in detail.

### 6.1. Stress Life (S-N) Method

The stress life (S-N) approach, also referred to as the Wöhler method, is one of the oldest and most widely used techniques for predicting fatigue life. It establishes the relationship between the number of cycles to failure (N) and the applied cyclic stress amplitude (S) [121]. This method is particularly well suited to components subject to high cycle fatigue (HCF), where deformation is mainly elastic and the number of loading cycles typically exceeds 10^4^–10^5^. S-N curves are generated experimentally by testing multiple specimens under constant-amplitude cyclic loading until failure occurs. Fatigue life is significantly reduced as stress amplitude increases. Steels and other ferrous materials frequently show a fatigue (endurance) limit, below which failure does not occur regardless of the number of cycles applied, whereas aluminum alloys generally do not exhibit a true endurance limit, and therefore require life prediction at a specified number of cycles [8]. Equation (1) is termed Basquin’s equation.

(1) $$\sigma_{a}=\sigma_{f}^{\prime }(2N_{f}{)}^{b}$$

where σ_a_, σ′_f_, N_f_ and b are stress amplitude, fatigue strength coefficient, number of cycles to failure, and fatigue strength exponent, respectively. Equation (1) yields a straight line on log-log axes, and this linearity underlies the S–N curves used throughout the design codes, as discussed in Section 1.5. This approach is considered simple, requires minimal data, and is easy to implement. It is a well-established, reliable tool for high-cycle fatigue in the linear elastic regime, and is incorporated into international design standards. However, in the case of low-cycle fatigue, it performs poorly because it does not capture notch stress concentrations and is also sensitive to load spectra [122].

The discussion above centers on the conventional high-cycle fatigue (HCF) regime (below 10^7^ cycles); however, with technological advancements, components such as high-speed rail axles and aero-engine parts are increasingly required to survive beyond this limit, into the very-high-cycle fatigue (VHCF) regime (10^7^–10^9^ cycles and beyond), where the classical assumption of a fatigue limit no longer holds [40]. Ultrasonic fatigue testing at loading frequencies around 20 kHz has made the VHCF characterization of welded joints practically feasible; welded joints retain a substantially lower VHCF strength than the parent metal, welding defects strongly influence the scatter of VHCF life, and internal, sub-surface “fish-eye” crack initiation becomes more prevalent beyond the conventional HCF range [40]. A direct comparison of ultrasonic and conventional low-frequency testing on flux-cored arc-welded steel joints confirms that fracture can still occur well beyond 10^7^ cycles, and that VHCF life estimated from ultrasonic testing without a frequency correction can be unconservative relative to notch stress-based design predictions [123]. S-N-based design curves calibrated principally against HCF data should therefore be applied with caution when welded components are expected to accumulate service lives in the VHCF regime.

Critically, the S-N method’s core assumption that fatigue life can be characterized by a nominal or classified stress range independent of the actual local geometry is precisely what welded joints stress most severely. Weld toe and weld root stress concentrations, geometric imperfections such as misalignment, undercut, or an unfavorable reinforcement angle, and near-surface tensile residual stresses from welding are not represented explicitly in the nominal stress; instead, they are implicitly absorbed into the chosen joint classification S-N curve. The method’s accuracy therefore depends entirely on how closely the actual joint matches the idealized geometry and the residual stress state the classification curve was originally derived from, and a joint with atypical imperfections can fall outside the calibrated scatter band without the method itself providing any warning that this has occurred. Its principal appeal—minimal input data (a classified detail category and an applied stress range) and negligible computational cost—is therefore also its principal risk for welded structures, since it offers no mechanism to flag when these convenient simplifications no longer hold.

### 6.2. Strain Life (ε-N) Approach

The strain life approach, commonly known as the Coffin–Manson method, is mainly used for low-cycle fatigue (LCF), where considerable plastic deformation occurs during cyclic loading. Unlike S-N methods, which consider only stress, ε-N approaches evaluate both the elastic and plastic strain components. Because of severe stress concentrations, plastic strain tends to concentrate near the weld toe in welded sheet metals; as a result, local strain-based methods generally provide more accurate fatigue predictions than nominal stress methods under overloads or variable-amplitude loading [122]. This method is well suited to predicting crack initiation and accounts for cyclic plasticity, although the cost of experimental testing is relatively high and it is less practical for very large structures. The Coffin Manson relationship itself is given in Equation (2) [124].

(2) $$\frac{\Delta \varepsilon }{2}=\frac{\sigma_{f}^{\prime }}{E}(2N_{f}{)}^{b}+\varepsilon_{f}^{\prime }(2N_{f}{)}^{c}$$

As Equation (2) shows, the total strain amplitude (Δε/2) is expressed as the sum of an elastic term, governed by the fatigue strength coefficient σ′f, Young’s modulus E, the fatigue strength exponent b, and a plastic term, governed by the fatigue ductility coefficient ε′f and the fatigue ductility exponent c. At short lives, the plastic term dominates, while at long lives the elastic term controls the response, so that Equation (2) converges towards Basquin’s equation (Equation (1)) as the number of reversals increases [122].

A critical limitation specific to welded structures is that applying the ε-N approach requires a well-defined local strain history at the location of interest, typically obtained by converting an assumed elastic stress concentration factor into a local notch strain (e.g., via Neuber’s rule or the Glinka energy method). For welded joints, the effective notch geometry at the weld toe is itself variable due to weld-profile imperfections, and the conversion is further complicated by superimposed residual stresses that shift the local mean strain; this notch strain conversion step introduces additional uncertainty that Equation (2) does not itself quantify. The high experimental cost of strain-controlled testing compounds this problem in practice; because calibrating Equation (2) for a specific weld detail is rarely repeated for every joint geometry and residual stress condition actually encountered in service, the ε-N parameters used for welded joint assessment are often transferred from parent material or generic weld detail data, rather than measured for the joint being assessed, weakening the method’s claimed advantage in accuracy over nominal stress approaches.

### 6.3. Fracture Mechanics-Based Prediction

Fatigue prediction based on fracture mechanics assumes that a component already contains a minor flaw or crack. This approach focuses on predicting crack propagation to failure rather than crack initiation. The driving force for crack growth is the stress intensity factor range, defined in Equation (3) [47].

(3) $$\Delta K=Y\Delta \sigma \sqrt{\left(\pi a\right)}$$

where Y is a geometry correction factor, a is the crack length, and Δσ is the stress range. Once ΔK is known, the rate at which an existing crack grows per cycle is given by the Paris law of Equation (4) [47].

(4) $$da/dN=C(\Delta K{)}^{m}$$

where C and m are material-dependent crack growth constants determined experimentally from da/dN–ΔK test data, of the type presented earlier in Figure 4d,e. The exponent m typically lies between 2 and 4 for structural steels and governs how sharply the growth rate accelerates as ΔK increases. Statistical analyses of C and m across weld steel datasets show the two parameters to be strongly correlated and the resulting fatigue crack growth life to follow a Weibull distribution [125]. Integrating Equation (4) from an initial to a critical crack size gives the propagation life Np, and Equation (5) combines this with the initiation life Ni to give the total fatigue life [43].

(5) $$N=N_{i}+N_{P}$$

where Ni is the crack initiation life and Np is the crack propagation life. Because welding defects are typically already present, the initiation life of welded structures is generally short, so total fatigue life is dominated by crack propagation, meaning that Equation (4) governs most of the service life predicted by Equation (5) [43].

Beyond the initiation/propagation split assumed in Equation (5), applying fracture mechanics to welded joints is critically sensitive to two inputs that are difficult to specify with confidence. The first is the assumed initial crack size a_0_, typically taken from a generic fitness-for-service default rather than measured directly in a given joint, even though predicted life is highly sensitive to a_0_ near the initiation-dominated end of the integration. The second is the residual stress-modified effective stress intensity factor discussed in Section 5.3; Y and Δσ in Equation (3) do not by themselves capture the crack closure and redistribution effects that a superimposed weld residual stress field introduces, so a nominally identical ΔK can correspond to markedly different actual crack growth rates depending on the (usually unmeasured) residual stress state of the joint. Because current standards offer only generic, conservative default flaw sizes rather than joint-specific values, and this assumption-driven uncertainty is rarely propagated through to the reported fatigue life, fracture mechanics-based predictions for welded structures can appear more mechanistically rigorous than the empirically calibrated S-N approach, while in practice carrying comparable, simply less visible, uncertainty.

### 6.4. Structural Hotspot Stress Method

The structural hotspot stress method predicts fatigue by measuring the structural stress at the weld toe while excluding the local notch effect. The hotspot stress is calculated using finite-element-based surface extrapolation, given in Equation (6) [126].

(6) σHS=1.67σ0.4t−0.67σ1.0t

where the reference distances from the weld toe are expressed relative to the plate thickness (t). As Equation (6) indicates, the hotspot stress σHS is obtained by linearly extrapolating the surface stresses measured at 0.4 t and 1.0 t from the weld toe back to the toe itself, removing the highly localized notch stress peak while retaining the structural stress concentration due to the joint geometry. The calculated hotspot stress is compared with standardized S-N curves developed specifically for welded joints [127].

A critical assumption underlying Equation (6) is that the notch effect being excluded by extrapolation is genuinely confined to the weld toe and does not extend into the 0.4 t–1.0 t extrapolation window itself. For thin welded sheets with sharp geometric imperfections such as undercut or poor toe-profile blending, or for joints whose weld-toe angle deviates substantially from the profile assumed in the reference S-N curves, this assumption can break down; the extrapolation then removes only part of the true notch effect, and the method underestimates the effective local stress. The method also assumes that surface-extrapolated stress adequately represents the through-thickness stress state, which is questionable for thicker joints or where residual stress varies significantly through the thickness. More broadly, the reference S-N curves used with the hotspot stress implicitly embed a representative, rather than joint-specific, level of residual stress and geometric imperfection, so the method’s accuracy is bounded by how representative these built-in assumptions are of the actual joint being assessed—information the method itself provides no means of checking.

### 6.5. Effective Notch Stress Approach

The effective notch stress (ENS) method accounts for local stress concentration by introducing a reference notch radius of 1 mm. The maximum notch stress at the weld toe is determined using finite element analysis, with the resulting stress concentration factor defined in Equation (7).

(7) $$K_{t}=\sigma_{max}/\sigma_{nom}$$

Here, σmax is the peak notch stress obtained from the finite-element model at the fictitious reference radius and σnom is the nominal applied stress; Equation (7) therefore quantifies, in a single dimensionless factor, how strongly the local weld geometry amplifies the nominally applied load. Because it directly represents weld geometry, the ENS method provides a strong correlation with fatigue test results and high prediction accuracy, making it well suited to complex weld details. A key limitation is that it requires precise modeling of the weld geometry [128].

The 1 mm reference radius idealization at the core of Equation (7) is itself a critical simplification with mixed implications for welded structures. It was calibrated primarily against joints with weld toe radii larger than a few tenths of a millimeter, so it can be unconservative for very sharp, poorly profiled weld toes or for thin sheets where the actual notch radius approaches or falls below the reference value, while conversely it can be overly conservative where post-weld treatments (Section 6.8) have deliberately increased the local radius beyond what the fictitious 1 mm notch represents. The requirement for precise finite-element modeling of the weld geometry, noted above as the method’s key limitation, is not merely a modeling inconvenience, but is a genuine practical barrier for welded metal sheet structures: the as-built weld profile (toe angle, undercut, reinforcement height) varies joint to joint, and is rarely measured in enough detail to build a geometry-accurate model, so in practice the method is usually applied to an assumed rather than as-built weld profile, reintroducing much of the geometric uncertainty it was designed to eliminate.

### 6.6. Critical Plane Methods

**Figure 12:**
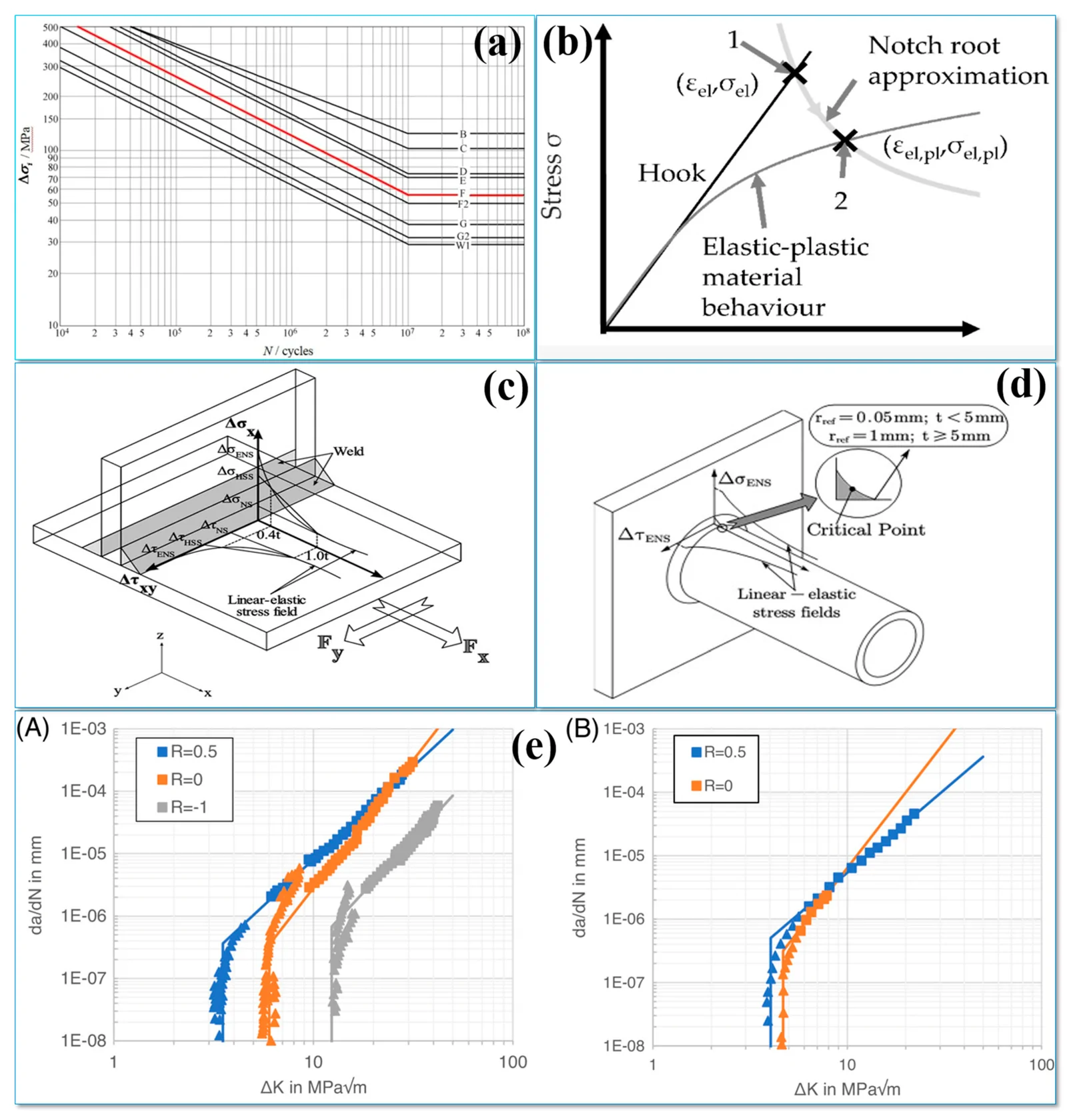
Comparative summary of conventional fatigue assessment methods for welded joints. (**a**) IIW fatigue class (FAT) design S-N curves for weld detail categories B through W1, plotted as nominal stress range against cycles to failure (adapted from [132] under CC-BY). (**b**) Notch root stress–strain approximation showing the linear elastic (Hooke) estimate and the corrected elastic–plastic material response used in strain life assessment (adapted from [133] under CC-BY). (**c**) structural hotspot stress definition, showing the extrapolation of the linear elastic surface stress field ahead of the weld toe [134]. (**d**) Effective notch stress method, showing the fictitious reference radius (r_ref_ = 1 mm for plate thickness t ≥ 5 mm; r_ref_ = 0.05 mm for t < 5 mm) applied at the critical point of the weld toe or root [129]. (**e**) Fatigue crack growth rate (da/dN) versus stress intensity factor range (ΔK) for two weld conditions at stress ratios R = −1, 0 and 0.5, illustrating the sensitivity of crack growth data to loading ratio [135].

Critical plane methods predict fatigue by identifying the material plane experiencing the greatest fatigue damage. Fatigue cracks typically initiate on planes where the combination of shear and normal stress is most damaging. This approach accounts for mean-stress effects and can predict crack orientation, but it involves complex implementation and requires multiaxial material testing [129]. The Modified Wöhler Curve Method (MWCM), one of the most established bi-parametric critical plane approaches for welded joints, was originally proposed by Susmel et al. to estimate the fatigue lifetime of steel and aluminum welded connections under variable-amplitude multiaxial fatigue loading [130]. A recent quantitative review has systematically compared stress-based critical plane criteria for welded joints under multiaxial fatigue loading—specifically Findley’s criterion, the Carpinteri–Spagnoli approach, and the Modified Wöhler Curve Method—evaluated in conjunction with the nominal stress, hotspot stress, effective notch stress, and Theory of Critical Distances point method stress analysis approaches, for both aluminum and steel welded joints under constant- and variable-amplitude multiaxial loading. The study found that all three criteria performed well for steel joints under constant-amplitude loading, with the Modified Wöhler Curve Method giving the most consistent overall accuracy across the joint types and loading conditions considered [129]. This quantitative benchmarking provides an up-to-date basis for selecting among critical plane criteria in the practical multiaxial fatigue assessment of welded sheet structures. Complementing these classical formulations, Beiler et al. combined analytical multiaxial fatigue models with machine learning regressors trained on multiaxial fatigue datasets for welded joints, and showed that the data-driven models matched or exceeded the predictive accuracy of the analytical critical plane criteria while requiring fewer simplifying assumptions about the governing damage mechanism, underscoring the growing complementarity between mechanistic and machine learning approaches to multiaxial fatigue assessment discussed further in Section 8.1 [131]. The five conventional prediction methods introduced in Section 6.1, Section 6.2, Section 6.3, Section 6.4 and Section 6.5 are drawn together visually in Figure 12, which juxtaposes the IIW design S-N curves, the notch root stress–strain approximation, the structural hotspot and effective notch stress definitions, and representative crack growth rate data, so that their relative conservatism and data requirements can be compared at a glance before the discussion in Section 6.7.

A critical practical barrier for welded structures specifically is that critical plane methods, strictly applied, require multiaxial fatigue data obtained from the weld toe or weld material microstructure itself, since welding measurably alters local hardness, residual stress, and microstructure in ways that can shift both the critical plane orientation and the associated damage parameter relative to the parent material. In practice, however, most published applications of MWCM and related criteria to welded joints substitute parent material or generic weld classification multiaxial data, because joint-specific multiaxial testing is prohibitively expensive for routine design use. The method’s theoretical rigor in predicting the crack initiation plane and life is therefore difficult to realize fully with the multiaxial data that are actually available for a given welded structure, and this gap between the method’s data requirements and what is typically supplied in practice is rarely made explicit when critical plane predictions are reported.

### 6.7. Comparative Assessment of Conventional Models

The assumptions, required inputs, computational complexity, and applicability of fatigue life prediction methods for welded sheet structures vary considerably.

The S-N method remains the most widely used method in engineering practice because it is simple, well established in design standards, and performs well for high-cycle fatigue under predominantly elastic loading, although it does not explicitly account for local stress concentration or crack formation. By including both elastic and plastic strain components, the strain life method extends fatigue analysis into the low-cycle region, making it more suitable for components undergoing cyclic plastic deformation [122]. Table 8 consolidates this comparison, summarizing the required input data, computational cost, applicable life regime, and key limitations of the six conventional fatigue life prediction methods introduced in Section 6.1, Section 6.2, Section 6.3, Section 6.4, Section 6.5 and Section 6.6, together with the governing literature for each method in its References column.

**Table 8:** Method–input–cost–applicability comparison of the conventional fatigue life prediction models.

Method	Required Input Data	Computational Cost	Applicable Life Regime	Key Limitation	References
Stress life (S-N/Wohler)	Constant-amplitude S-N curve, nominal stress amplitude	Low	High-cycle fatigue (HCF), predominantly elastic loading	Does not capture local notch stress concentration; sensitive to load spectrum; unconservative if extrapolated into VHCF without correction	[121,123,132]
Strain life (Coffin–Manson, e-N)	Cyclic stress–strain data; elastic and plastic strain components	Moderate (cyclic material testing)	Low-cycle fatigue (LCF), significant plastic deformation	Relatively costly experimental characterization; less practical for very large structures	[122,124]
Fracture mechanics-based (Paris law)	Initial/critical crack size, stress intensity factor range, C and m constants	Moderate-to-high (crack growth integration)	Propagation-dominated life; most of the service life of as-welded joints	Assumes a pre-existing flaw; initiation life is treated separately or neglected	[43,47]
Structural hotspot stress	FE surface stress extrapolation at 0.4 t and 1.0 t from the weld toe	Moderate (FE model required)	HCF; standardized welded-joint S-N curves	Extrapolation is mesh density-dependent; excludes the local notch effect	[126,127]
Effective notch stress (ENS)	FE model with fictitious reference radius (1 mm, or 0.05 mm for t < 5 mm) at the weld toe/root	High (fine mesh needed at the notch)	HCF; well suited to complex weld details	Requires precise geometry modeling; computationally demanding	[128,129]
Critical plane methods (e.g., MWCM)	Multiaxial stress/strain history; multiaxial material test data	High (complex implementation)	Multiaxial constant- and variable-amplitude loading	Complex to implement; requires dedicated multiaxial testing	[129,130,131]

Table 8 compares these methods by input, cost, and limitation, but it is equally important to connect them to how a practicing engineer actually selects among them in design, since the five approaches (excluding critical plane methods, which apply specifically to multiaxial loading rather than sitting in this uniaxial escalation path) function less as independent alternatives than as a tiered escalation path written directly into design standards. Nominal stress (S-N) assessment is the default starting point whenever a joint matches a standard classified detail in the tables provided by IIW, Eurocode 3, or AWS D1.1, and is applied at the earliest design stage, before any detailed finite element model of the joint exists. When a joint’s geometry falls outside these classified detail tables—a non-standard connection, an unusual load path, or a detail the applicable code does not tabulate—IIW recommendations direct the assessment to escalate to the structural hotspot stress method, which requires a shell or solid finite element model to already be in hand. The effective notch stress method is reserved for a further tier; complex or small-scale weld details where even hotspot extrapolation is ambiguous, or where a single, geometry-independent S-N curve applicable across many joint types is preferred, at the cost of the fine mesh resolution Table 8 identifies as its key practical burden. Local strain (ε-N) analysis is invoked not as a routine first choice, but specifically once a nominal or hotspot stress check indicates that local plastic strain is expected—that is, once the assessment moves from the high-cycle regime these stress-based methods assume into low-cycle territory. Fracture mechanics, by contrast, is used less at the initial design stage and more for in-service fitness-for-service assessment, inspection interval planning, and defect-acceptance evaluation once a flaw has actually been detected or must be conservatively assumed present, drawing on standards such as BS 7910 rather than the classified detail tables that govern the stress-based methods above. Read this way, the five methods in Table 8 trace a single practical design workflow—nominal stress by default, escalating to hotspot or notch stress for non-standard geometry, to local strain once plasticity is flagged, and to fracture mechanics once a defect is known or assumed—rather than five competing techniques a designer must choose among from scratch for every joint.

### 6.8. Structural Stress and Post-Weld Treatment-Based Models

Beyond the classical hotspot and notch stress approaches introduced in Section 6.4 and Section 6.5, mesh-insensitive structural stress formulations have been developed to remove the dependence of hotspot extrapolation on finite element mesh density. Dong’s structural stress method equilibrates the through-thickness stress distribution at a weld toe using shell or solid element nodal forces and moments, producing a structural stress parameter that collapses fatigue data from many different joint geometries and loading modes onto a single master S-N curve, which substantially reduces the scatter seen with conventional hotspot extrapolation [136,137]. This approach builds on, and is consistent with, the structural stress determination guidance originally proposed by Niemi for the IIW [127] and the local approach philosophy developed by Radaj, which treats fatigue assessment as a hierarchy of increasingly local stress measures, from nominal through structural to notch stress [29]. Because as-welded joints typically fail from the weld toe under a severe local stress concentration, post-weld improvement techniques are used to shift the S-N curve to longer life for a given stress range. Grinding-based methods reshape the weld toe to remove the sharpest notch and any embedded toe defects, and recent statistical assessments have quantified the achievable fatigue strength improvement from the flush grinding of butt welds across large experimental datasets [138]. High-frequency mechanical impact (HFMI) treatment, which uses a vibrating pin to plastically deform the weld toe, has emerged as the most extensively validated improvement technique; it simultaneously increases the local toe radius, work-hardens the surface layer, and introduces beneficial compressive residual stresses, and its fatigue benefit has been quantified through local notch stress and structural stress approaches across a wide range of steel grades and loading ratios [139]. These findings have been consolidated into structured fatigue assessment guidelines for HFMI-treated joints [140], culminating in dedicated IIW recommendations that specify treatment procedures, quality control criteria, and design S-N curves for HFMI-improved welds [141]. These recommendations have since been substantially updated; the second edition of the IIW HFMI guidelines extends applicability to steels with yield strengths from 960 to 1300 MPa and plate thicknesses of 5–50 mm, relaxes several pre- and post-treatment geometric requirements, and introduces numerical fatigue strength modification factors and a methodology for verifying HFMI device effectiveness—the most significant revision of the HFMI guidance since the original 2016 recommendations [142].

### 6.9. Foundational Damage Models and Probabilistic Fatigue Assessment

Many of the conventional methods described above rest on foundational fatigue concepts developed across the twentieth century. Miner’s linear cumulative damage rule, introduced in Section 2.5, remains the most widely applied damage summation law in practice [45], while the log-linear relationship between stress amplitude and cycles to failure that underlies the S-N method was first formalized by Basquin [121] and extended into the low-cycle, strain-controlled regime through Manson’s approximations of fatigue behavior in terms of ductility and strength parameters [143]. The fracture mechanics view of fatigue traces back to Griffith’s energy balance criterion for crack propagation in brittle solids [44] and Irwin’s subsequent formulation of the near-tip stress field in terms of the stress-intensity factor, which underpins Paris’ law and the crack growth calculations of Section 6.3 [22]; the practical implementation of these concepts for surface and embedded flaws in plates relies on empirical stress intensity factor solutions, such as those derived by Newman and Raju [144], while the treatment of mean stress effects in strain-based fatigue analysis draws on Morrow’s classical mean stress correction [145]. These classical laws are deterministic; their probabilistic counterparts, such as the systematic probabilistic S-N and fatigue limit models developed for welded steel joints [146], provide survival probabilities for a given service life, allowing inspection intervals to be set proactively. These classical damage summation approaches serve as a baseline, and more recent work has developed systematic probabilistic S-N models specifically for welded steel joints under constant-amplitude loading, building on this foundation. These models also include a probabilistic Fatigue Limit Model, which treats fatigue life and the fatigue limit as random variables rather than a single deterministic S-N curve [146]. This new formulation provides a more statistically rigorous basis than the classical deterministic approach.

### 6.10. Applicability of Predictive Methods Across Fatigue Stages

Fatigue crack growth is more accurately described by three mechanistically distinct stages—crack initiation, short crack propagation and long crack propagation—rather than the single, Paris law-governed “stable crack growth” phase of Table 4. Crack initiation is governed by cyclic slip, persistent slip band formation, and microstructural barriers such as grain boundaries and inclusions, and typically accounts for a large fraction of total life in the high-cycle regime under low nominal stress. Short-crack propagation covers roughly the first tens to a few hundred micrometers of growth from a nucleated crack; over this range, cracks frequently grow faster than the long crack da/dN–ΔK curve of Figure 4d would predict at the same nominal ΔK, and can even propagate below the long crack threshold ΔKth, because the crack closure mechanisms (plasticity-induced, oxide-induced, and roughness-induced closure) that reduce the effective driving force for long cracks have not yet fully developed. This “short crack anomaly” is classically represented on a Kitagawa–Takahashi diagram, which plots the transition from a microstructurally controlled threshold stress at very short crack lengths to the LEFM long crack threshold as the crack grows. Long crack propagation, by contrast, is the regime in which linear elastic fracture mechanics (LEFM) assumptions hold and Paris’ law directly applies, corresponding to Region II of the da/dN–ΔK curve. Because these three stages are governed by different mechanisms, no single method reviewed in Section 5 and Section 6 addresses all of them equally well; Table 9 makes this mapping explicit for the S-N, strain life, fracture mechanics, cohesive zone, XFEM, and monitoring-based approaches discussed throughout this review, together with the primary limitation each method faces outside its best-suited stage.

## 7. Experimental Characterization and Structural Health Monitoring

Welded sheet structures are highly prone to fatigue failure under cyclic loading, as stress concentrations at the weld toe and heat-affected zone accelerate crack initiation and propagation. This study reviews the experimental characterization and structural health-monitoring techniques that enable accurate fatigue life prediction and early detection of structural degradation in welded joints.

### 7.1. Fatigue Testing

Fatigue testing is used to evaluate the durability of welded metal sheets under cyclic loading. Standardized test methods ensure reliable and consistent results that can be compared across different studies. ASTM E466 (constant-amplitude fatigue testing) [147], ASTM E647 (characterizing fatigue crack growth) [148], and ISO 12107 (statistical analysis and processing of fatigue data) [149] are commonly used standards for such purposes. Typically, servo-hydraulic or electromechanical test equipment is used to test specimens under various loading conditions. In addition, accurate data acquisition and careful specimen preparation are essential for updating structural design and fatigue-life prediction models.

### 7.2. Digital Image Correlation (DIC)

Digital image correlation (DIC) is another non-contact optical technique used in mechanical testing to determine full-field displacement and strain distributions on a specimen’s surface. A random speckle pattern is applied to the surface and continuously photographed by high-resolution cameras throughout the test [150]; specialized software then tracks the deformation of the speckle pattern between images to accurately reconstruct the strain field. This technique is widely used in fatigue research to observe crack formation on joint surfaces, identify local strain hotspots, and detect crack initiation; owing to its high spatial resolution and non-invasive nature, it is also considered an essential tool for validating numerical simulations and fatigue models [92].

### 7.3. Acoustic Emission (AE) Monitoring

**Figure 13:**
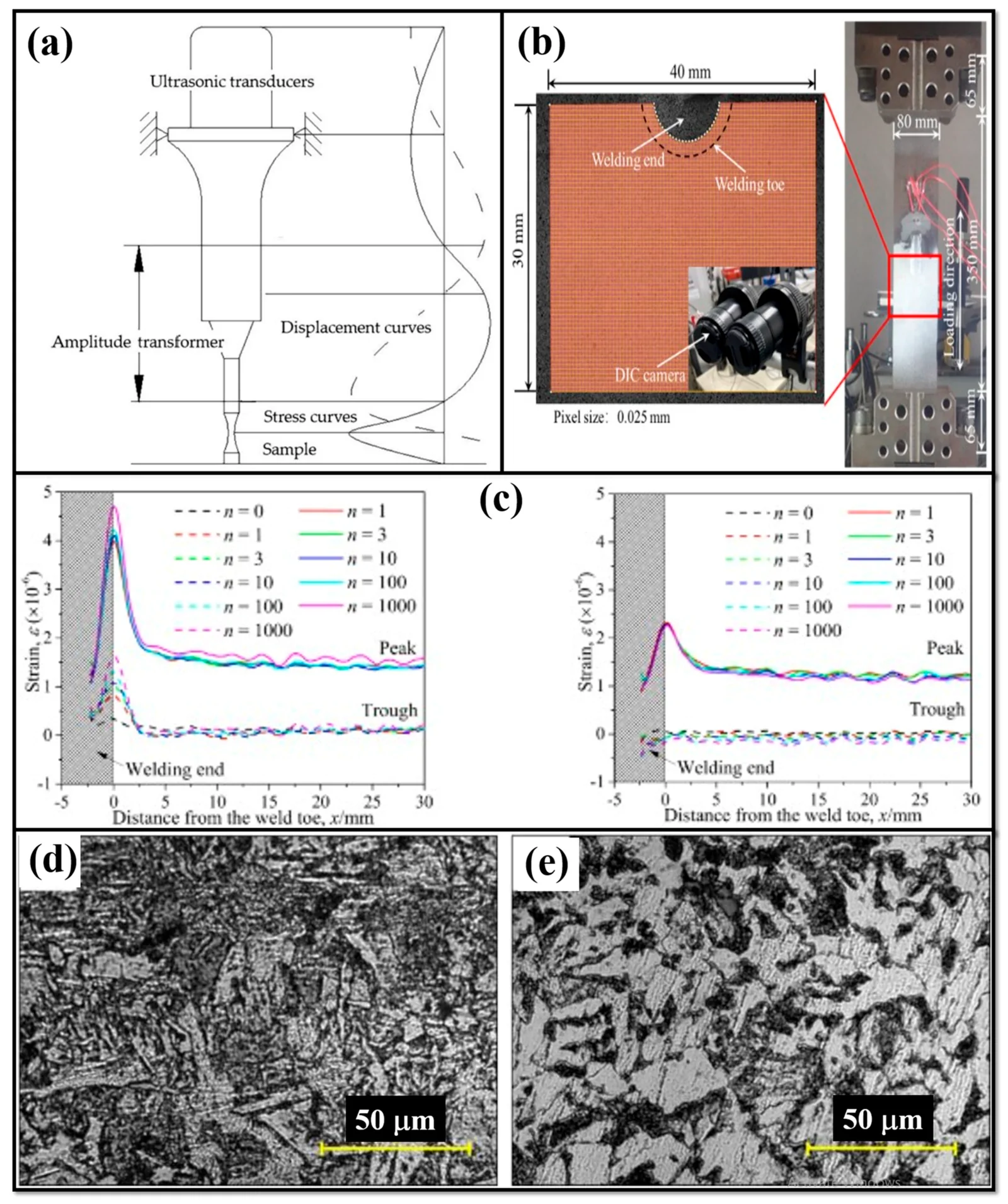
Ultrasonic fatigue testing and digital image correlation (DIC) strain monitoring of welded specimens. (**a**) Schematic of an ultrasonic fatigue-testing rig, showing the ultrasonic transducers, amplitude transformer, and specimen, together with the resulting displacement and stress distributions along the sample length (adapted from [152] under CC-BY). (**b**) DIC test setup on a welded coupon, showing the welding toe region, the speckle pattern used for strain tracking, and the DIC camera rig mounted on the loading frame (adapted from [154] under CC-BY). (**c**) DIC-measured strain versus distance from the weld toe at peak and trough loads for increasing numbers of cycles (*n* = 0 to 1000), showing strain concentration and redistribution at the weld toe as cycling progresses (adapted from [154] under CC-BY). (**d**,**e**) Associated microstructural evolution of the weld/HAZ grain structure (adapted from [153] under CC-BY).

Acoustic emission (AE) monitoring is a continuous, passive testing technique that detects elastic stress waves generated by processes such as crack formation, plastic deformation, crack growth, and other forms of material damage. Sensors mounted on the surface of a welded component continuously record acoustic signals as the component is subjected to cyclic loading. Energy, amplitude, and event count are key parameters that provide valuable insight into structural damage progression, and the technique is particularly effective for monitoring critical infrastructure, such as bridges, aircraft, pipelines, and pressure vessels, owing to its ability to detect fatigue early [151]. The complementary strengths of ultrasonic excitation and optical strain mapping are illustrated in Figure 13, which shows how an ultrasonic fatigue testing rig generates the very high cycle counts needed for gigacycle fatigue testing (Figure 13a) [152], while DIC captures the resulting strain concentration and redistribution at the weld toe as cycling progresses (Figure 13b,c), alongside the associated microstructural evolution of the weld/HAZ grain structure (Figure 13d,e) [153].

### 7.4. Infrared Thermography

Among non-contact inspection techniques, infrared thermography employs infrared cameras to measure surface temperature distributions, detecting the localized heating produced as plastic deformation and crack growth dissipate energy during cyclic loading. These thermal variations help identify fatigue damage before visible cracks appear, since infrared thermography allows rapid inspection without interruption, and is well suited to the large-scale monitoring of welded structures; it is widely used in industrial maintenance, civil engineering, and aerospace applications [155].

### 7.5. Ultrasonic Inspection

Ultrasonic inspection is a non-destructive evaluation technique that is widely used for detecting surface and internal defects in welded joints. It also provides information on the location and size of imperfections such as cracks, porosity, and inclusions by analyzing the reflections of high-frequency ultrasonic waves directed into the material. Advanced evaluation techniques such as time-of-flight diffraction (TOFD) and phased-array ultrasonic testing (PAUT) provide higher accuracy and improved defect characterization, which is why ultrasonic inspection is widely used for quality control, structural integrity assessment, and the routine maintenance of welded structures subjected to fatigue loading [156].

#### Eddy Current and Guided Wave Inspection

Beyond conventional ultrasonics, eddy current testing (ECT) and guided wave ultrasonic testing (GWUT) provide complementary coverage of welded structures. Trung et al. developed a flexible eddy current array sensor with bendable channels that detects surface and sub-surface cracks in steel weld zones in real time, including through non-conductive coatings, making it well suited to the in-service inspection of curved or coated welded components [157]. For long, continuous structures such as pipelines, Lyu et al. reviewed how guided waves propagate along the structure with low attenuation, allowing a single sensor position to screen extended lengths of weld for corrosion and fatigue-relevant defects, at the cost of lower spatial resolution than point-wise ultrasonic or eddy current scanning [158]. Guided waves can also be used actively rather than only in a passive screening role; an active structural health-monitoring system built around a permanently mounted piezoelectric sensor network excited fatigue cracks directly in the weld zone of a welded T-shaped bogie-frame specimen, and tracked crack growth as small as 2 mm through a damage index computed from the guided wave signals, indicating that permanently installed guided wave networks can complement the eddy current and pipeline-screening applications above for the continuous, in-service fatigue crack monitoring of welded joints [14].

### 7.6. X-Ray-Computed Tomography (XCT)

X-ray-computed tomography (XCT) is another non-destructive, advanced imaging technique that generates three-dimensional images of the internal structure of welded components. Using this technique, inclusions, pores, lack of fusion, cracks, and other manufacturing imperfections that can affect fatigue performance can be characterized in fine detail. Compared with conventional radiography, XCT provides volumetric data that enable the quantitative analysis of the shape, size, and distribution of defects. Because it allows accurate correlation between internal defects and fatigue crack initiation sites, XCT has become increasingly valuable in fatigue research [159].

### 7.7. Fiber-Optic Sensing Technologies

Fiber-optic sensing technology enables the continuous and highly detailed monitoring of temperature, pressure, and structural strain in welded components. Among these, fiber Bragg grating (FBG) sensors are the most widely used owing to their high sensitivity, small size, and resistance to electromagnetic interference. These sensors can be permanently embedded within structures to provide continuous health monitoring throughout their service life, and are commonly used in offshore platforms, bridges, wind turbines, and aerospace structures, where long-term structural integrity is essential [160,161].

FBG sensors have specifically been deployed on the fatigue-prone welded joints of in-service steel structures rather than only in laboratory coupons; on an orthotropic steel deck arch bridge, an array of 64 FBG sensors instrumented around the rib-to-deck and rib-to-diaphragm welded joints has been used to derive vehicle- and temperature-induced stress histories and the hotspot stress at the weld detail directly from long-term field data, illustrating how fiber-optic sensing can feed the structural hotspot stress method of Section 6.4 with in-service rather than laboratory-only stress spectra [161]. At the crack-monitoring scale, a complementary study combined FBG sensors with digital image correlation, extracting the spectral area of the FBG reflection spectrum as a damage feature and cross-validating the resulting crack location estimates against DIC-measured strain fields around a propagating fatigue crack, demonstrating the value of coupling fiber-optic and optical strain mapping techniques, rather than treating DIC (Section 7.2) and fiber-optic sensing as independent monitoring channels [162].

### 7.8. In-Situ Crack Monitoring

**Figure 14:**
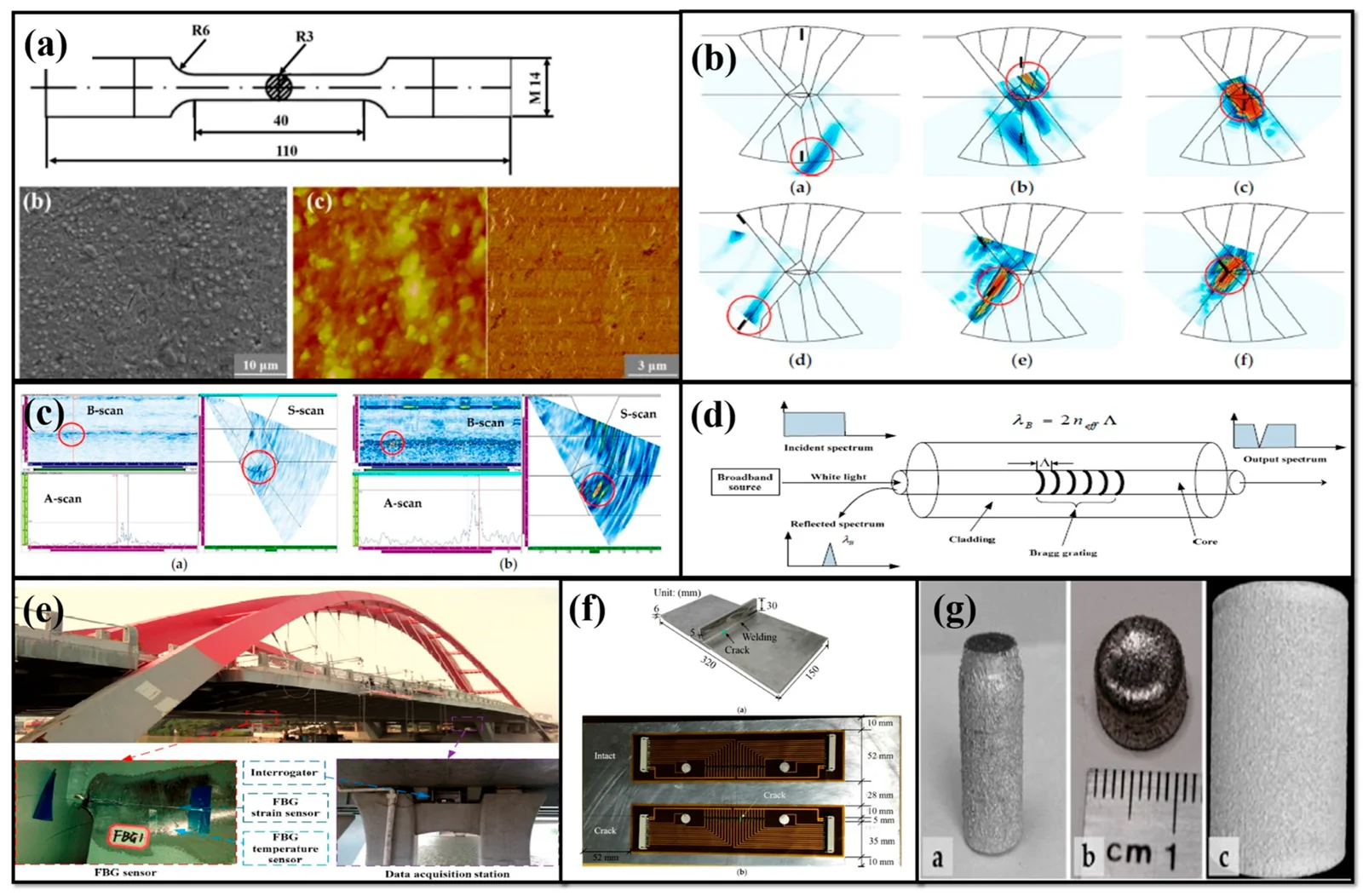
Multi-sensor in-situ crack monitoring techniques for welded and metallic fatigue specimens. (**a**) Fatigue test specimen with acoustic emission (AE) sensor mounted near the notch region (adapted from [164] under CC-BY). (**b**) S-scan results of flaw detection simulation using transverse waves at a frequency of 4 MHz (adapted from [156] under CC-BY). (**c**) experimental sectorial scan results for top crack with 7.5 MHz transverse wave and 4 MHz transverse wave (adapted from [156] under CC-BY). (**d**,**e**) Fiber Bragg grating (FBG) sensing principle, showing the broadband source, cladding/core grating structure, and reflected/output spectra, together with an in-service deployment on a bridge girder incorporating FBG strain and temperature sensors and a data acquisition interrogator (adapted from [161] under CC-BY). (**f**) Fiber-optic crack sensor patches applied to a cracked and an intact welded steel plate for comparison (adapted from [162] under CC-BY). (**g**) Shapes of the tested samples made by liquid metal jetting from aluminum by directed energy deposition from Inconel and by laser-based powder bed fusion from titanium alloy (adapted from [165] under CC-BY).

In-situ crack monitoring refers to the continuous observation of crack initiation and propagation when a welded structure is subjected to laboratory or in-service loading. The techniques discussed earlier, such as digital image correlation (DIC), acoustic emission (AE), ultrasonic inspection, electrical potential drop methods, and fiber-optic sensors, enable the real-time monitoring of damage without interrupting operation. In situ monitoring provides information on crack growth rate, remaining useful life, and maintenance planning, and it significantly improves structural safety by enabling the early detection of fatigue damage before catastrophic failure occurs [163]. Figure 14 illustrates how several of these techniques are combined in practice—acoustic emission source localization tracking a crack as it initiates and propagates through a specimen; phased-array ultrasonic B- and C-scans characterizing flaw position, orientation, and signal intensity in anisotropic welded joints; fiber Bragg grating sensors providing continuous strain and temperature monitoring, from laboratory demonstration through to field-deployed bridge girder installations; and post-test fracture surfaces confirming the failure mode across specimens produced by different manufacturing routes. Panel-by-panel detail is given in the figure caption.

### 7.9. Sensor Fusion for Fatigue Prognosis

Sensor fusion improves the accuracy and reliability of fatigue damage detection and remaining useful life estimation by combining data from multiple sensing systems. Advances in technology now allow modern techniques, including advanced algorithms and artificial intelligence-based machine learning methods, to integrate data from different sensing modalities, including acoustic emission, strain gauges, fiber-optic sensors, infrared thermography, digital image correlation, and vibration monitoring. This method provides a comprehensive structural health assessment by reducing measurement uncertainty and improving damage characterization, which is increasingly used in modern digital twin frameworks for the predictive monitoring and maintenance of welded structures under demanding fatigue conditions [117].

A distinction worth making explicit, echoing the technology-readiness discussion of digital twins in Section 8.1.2, is that most of the SHM and sensor fusion evidence reviewed in this section (Section 7.2, Section 7.3, Section 7.4, Section 7.5, Section 7.6, Section 7.7, Section 7.8 and Section 7.9) comes from laboratory specimens or short-term, single-structure field pilots, rather than from long-term, fleet-scale industrial deployment. Demonstrating that a sensing modality can detect or characterize a crack under controlled laboratory conditions, or on one instrumented bridge girder over a limited monitoring period, is a materially different claim from demonstrating that the same modality remains reliable across many nominally similar structures, multiple years of environmental exposure, sensor drift and occasional failure, and the operational variability of real service loading. Few of the studies discussed above report this kind of multi-structure, multi-year validation, and where field deployments are described, they typically remain individual case studies rather than a systematic industrial validation program with a defined false-positive/false-negative detection rate over time. This gap matters specifically for the sensor fusion and digital twin frameworks that depend on this evidence base; a fatigue-management system that fuses multiple sensing modalities and updates a remaining life estimate in real time is only as reliable as the individual sensing technologies it fuses, so the industrial-scale validation gap identified here for SHM directly compounds the digital twin maturity gap already discussed in Section 8.1.2, rather than being a separate, lower-priority concern.

## 8. Emerging Trends, Sustainability, and Industrial Applications

Fatigue failure remains one of the most critical challenges in the structural integrity of welded sheet components, accounting for a significant proportion of in-service failures across automotive, aerospace, and heavy industrial sectors. As lightweight design philosophies and high-strength materials become central to modern manufacturing, accurately predicting the fatigue life of welded joints has grown increasingly vital to ensuring both performance and safety. This review explores the emerging trends, sustainability considerations, and industrial applications shaping the future of fatigue life prediction in welded sheet metal structures.

**Figure 15:**
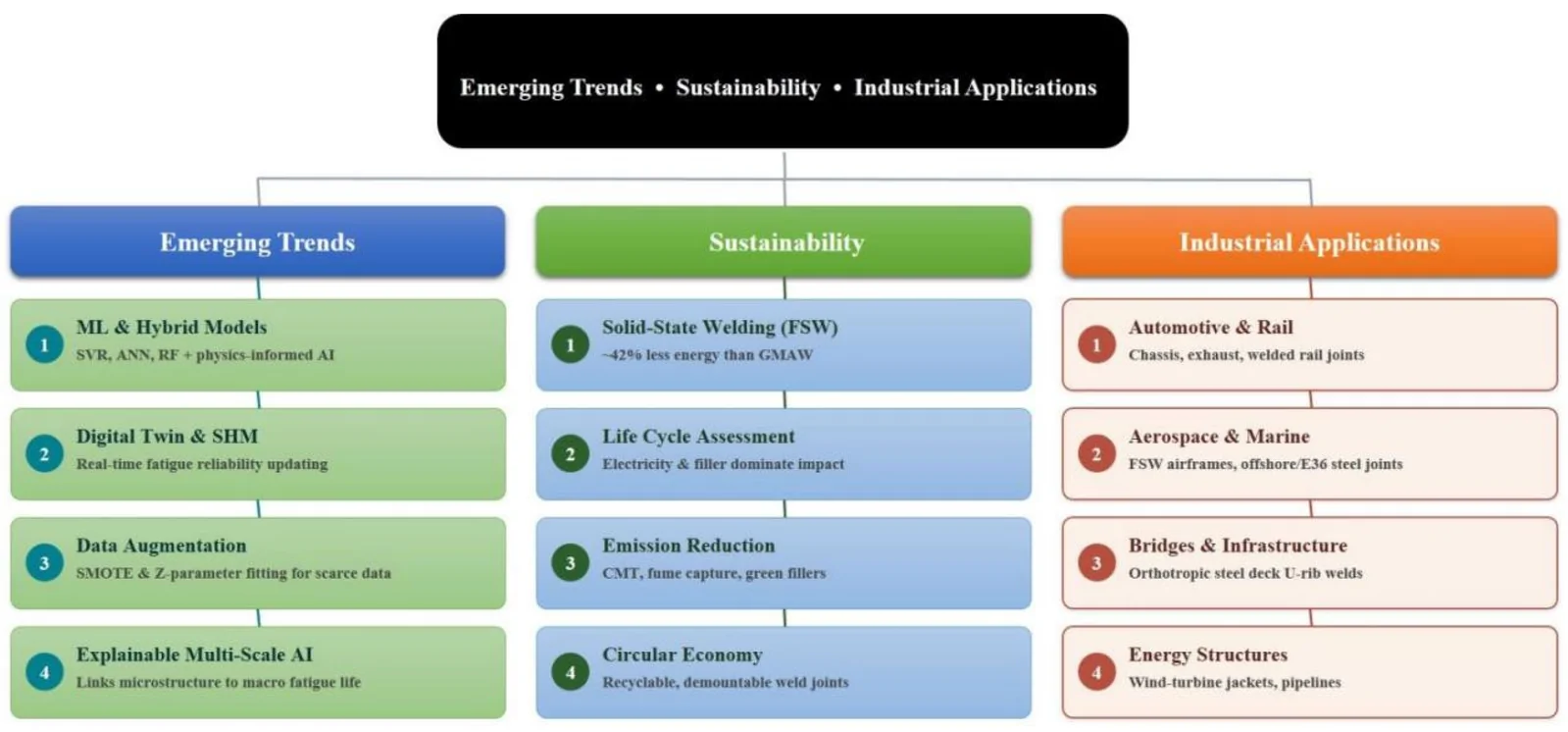
Emerging trends in fatigue life prediction integrate physics-informed AI, digital twins, SHM, data augmentation, and explainable multi-scale models to improve prediction accuracy and reliability. The framework also highlights sustainable welding practices, life-cycle assessment, emission reduction, circular design, and applications across automotive, aerospace, marine, infrastructure, and energy sectors.

The three branches of Figure 15 are best read as a single, coupled agenda rather than three independent topics. Section 8.1 addresses the predictive–modeling gap identified throughout Section 5, Section 6 and Section 7; conventional physics-based methods and even single-mechanism machine learning models struggle to generalize across the joint geometries, materials, and loading spectra encountered in practice, motivating hybrid, self-updating, and data-efficient prediction strategies. Section 8.2 addresses a constraint that is largely orthogonal to prediction accuracy but increasingly co-determines process selection in industry—the energy, material, and end-of-life burden of the welding process itself, which means that the “best” joining method for fatigue performance and the “best” method for sustainability are not always the same, and quantitative trade-off tools are needed to reconcile them. Section 8.3 then grounds both of these strands in practice, showing how the prediction methods and sustainability pressures reviewed above are actually being deployed, and with what degree of maturity, across four representative heavy industry sectors. Read together, these three branches indicate that the field is moving from validating individual prediction methods in isolation toward integrated frameworks that couple data-driven fatigue-life prediction, sustainability accounting, and sector-specific deployment constraints within a single design workflow.

### 8.1. Emerging Trends in Fatigue Life Prediction

Before surveying specific approaches, it is worth being explicit that “machine learning for fatigue” is not a single task. Damage detection asks whether, and where, damage is present, typically as a binary or multi-class classification learned from raw or lightly processed sensor signals. Damage classification goes a step further, categorizing detected damage by type or severity, again primarily a classification problem. Crack size estimation is a regression task mapping a sensor signal or image to a physical crack length, and requires paired ground truth measurements derived from the characterization techniques discussed in Section 7. Fatigue life prediction, the focus of most models discussed in Section 8.1.1 below, is a regression task mapping material, geometric, and loading variables to total cycles to failure, trained on S-N-type datasets. Remaining useful life (RUL) estimation is a related but distinct sequence-modeling task that updates a life estimate in real time from an evolving operational history, rather than predicting a fixed total life upfront, and is the natural output of the digital twin architectures discussed in Section 8.1.2. Maintenance decision support goes further still, combining an RUL or damage estimate with cost, risk, and logistical constraints in order to recommend an action, requiring optimization or decision-theoretic components beyond a purely predictive model. These six tasks require correspondingly different datasets (labeled signals or images for detection and classification; paired sensor–crack size measurements for crack sizing; S-N curves for life prediction; full operational histories for RUL), different model structures (classifiers, regressors, or sequence models such as recurrent networks and Bayesian filters), and different validation strategies (classification metrics such as precision and recall; regression metrics such as RMSE; prognostics-specific metrics such as the α-λ performance criterion; and decision quality metrics tied to cost or risk reduction). The subsections below remain organized primarily by method, consistent with the rest of this review, but each is flagged with the specific task or tasks it addresses to keep this distinction explicit; Section 8.1.6 additionally surveys uncertainty quantification and probabilistic fatigue assessment, which cut across several of these tasks rather than belonging to any single one.

Figure 15 illustrates that fatigue life prediction is shifting toward data-driven hybrid frameworks and away from purely physics-based approaches, because traditional methods such as nominal stress, structural hotspot stress, effective notch stress, and fracture mechanics approaches remain restricted by geometry, are computationally expensive, and are unable to self-improve [166]. This has motivated six emerging directions, as follows.

#### 8.1.1. ML and Hybrid Models

*[Primarily addressing fatigue-life prediction, per the task taxonomy above.]* Support vector regression (SVR), artificial neural networks (ANN), random forest (RF), and Gaussian process regression (GPR) are all regression-based algorithms now routinely used in place of classical empirical formulas because they consistently outperform classical methods in predictive accuracy [119,166]. Moreover, physics-based hybrid methods, which introduce damage tolerance into ML frameworks (e.g., SVR guided by physical crack growth laws), further improve accuracy by combining domain knowledge with data-driven learning [166]. A recent review focused specifically on welded joints similarly concluded that regression-based ML models are steadily displacing classical S-N and fracture-mechanics approaches as the preferred fatigue life prediction tool wherever sufficient training data exist, while noting that model interpretability and dataset scarcity remain the principal barriers to wider industrial adoption [167]. These general ML trends extend to multiaxial loading as well, as illustrated by the hybrid analytical–machine learning approach for welded joints discussed in Section 6.6 [131].

The predictive accuracy figures reported above, however, need to be read alongside a critical limitation that is often left implicit: a model’s reported accuracy is only demonstrated on the material, welding procedure, specimen geometry, and loading spectrum it was trained and tested on, and does not automatically transfer to a different welded structure. An SVR or ANN model trained on cruciform joints in one steel grade, for instance, is effectively interpolating within the feature space defined by that dataset; applied to a different plate thickness, joint geometry, welding process, or loading spectrum, it is extrapolating outside that space, where its accuracy is not guaranteed and is rarely characterized in the original study. This is not a hypothetical concern; because the training datasets available for welded joint fatigue remain small and geometry-specific, as discussed in Section 9.3, most published ML models have been validated on a narrow range of conditions relative to the diversity of welded structures encountered in practice, and few studies report how performance degrades outside the training distribution. The consequence is particularly important for industrial implementation, where a model would need to be applied, or at least revalidated, on structures the original training data did not cover; deploying a model without this revalidation risks a false sense of confidence, since the model provides a numerical prediction regardless of whether the input falls inside or outside its valid domain, with no inherent signal that it has been asked to extrapolate. Two of the strategies discussed later in this section respond directly to this problem rather than to accuracy alone—the transfer learning approaches in Section 8.1.5 are explicitly designed to adapt a model from a data-rich source domain to a data-scarce target domain, and the explainability methods in Section 8.1.4 can help reveal when a model is relying on features that are unlikely to generalize. Neither, however, removes the underlying requirement that any model intended for industrial use should be validated on data representative of its intended deployment conditions before that predictive capability is assumed rather than demonstrated.

#### 8.1.2. Digital Twin and Structural Health Monitoring

*[Spanning damage detection, remaining useful life estimation, and, in Section 8.4, maintenance decision support.]* A digital twin, in this context, is a virtual replica of a physical weld or welded structure that is continuously synchronized with real-world data, comprising three coupled elements: a physics-based or data-driven fatigue model, a live stream of sensor data from the physical asset (strain gauges, accelerometers, or the acoustic emission, DIC, and fiber-optic sensing methods discussed in Section 7), and an updating algorithm that reconciles the two. Wang et al. demonstrated this coupling directly, using a digital twin-driven framework in which model form uncertainty in the fatigue life prediction is quantified and continually narrowed as new monitoring data become available, rather than being fixed at the design stage [117]. Sensor-driven monitoring systems, paired with Monte Carlo simulation methods and Bayesian updating, allow fatigue reliability to be recalculated as a structure ages, in real time, rather than relying on a single design-stage estimate [117]. This approach has since been extended to welded infrastructure directly: Lao et al. built a digital twin framework that probabilistically predicts microcrack initiation and propagation in the weld zone of orthotropic steel bridge decks by continuously updating a fracture mechanics model with field-measured strain histories, giving asset owners a live estimate of remaining fatigue life rather than a static inspection interval schedule [168]. Peng et al. took a complementary approach, embedding the residual stress state of a welded structure, rather than treating it as a fixed input, directly into a digital twin fatigue life framework, since residual stress evolves with fabrication sequence and in-service loading, and is one of the largest sources of scatter in the model comparisons summarized in Section 6.7 and Section 6.8 [169]. Taken together, these case studies show digital twin fatigue assessment maturing from a conceptual framework into structure-specific implementations, though all three still depend on the sensing infrastructure discussed in Section 7 being permanently installed and maintained over the structure’s service life, which remains a practical barrier to widespread adoption.

**Figure 16:**
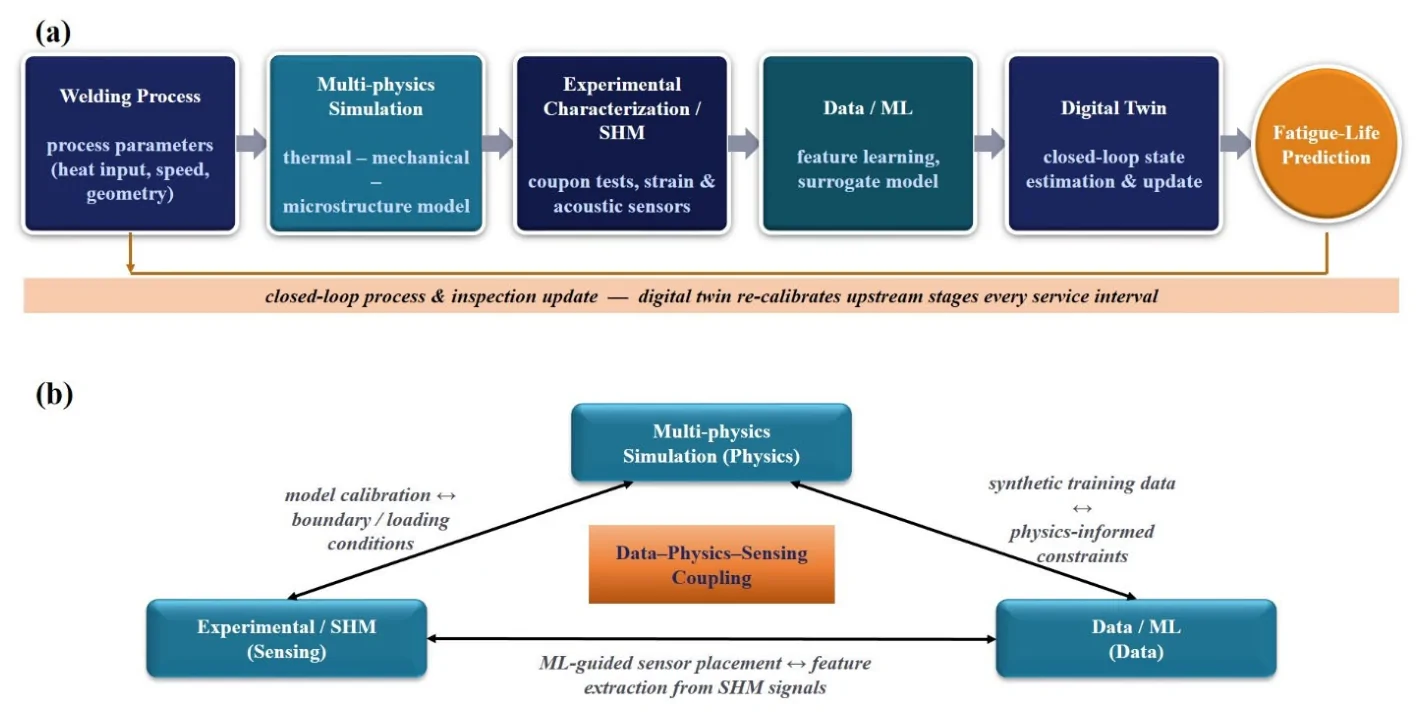
Proposed data–physics–sensing coupled digital twin framework for weld fatigue life prediction: (**a**) end-to-end pipeline from welding process to fatigue life prediction with closed-loop feedback, and (**b**) the explicit coupling mechanism linking physics, sensing, and data.

It is worth being precise about what “maturing” means here, since the distinction matters for how these results should be read. Each of the three studies above demonstrates one specific digital twin configuration—uncertainty-quantified model updating [117], fracture mechanics model updating from field strain data [168], or residual stress-aware state estimation [169]—validated on one specific structure, rather than a single, general-purpose fatigue digital twin shown to generalize across welded structures. In technology-readiness terms, these individual configurations sit at an early-to-mid research/demonstration level (validated in a specific case study or controlled setting) rather than at a deployment-ready level (routinely used across an asset fleet without bespoke setup). The more ambitious, fully integrated closed-loop architecture proposed in Section 8.4 (Figure 16), which couples simulation, sensing, and data in a single three-way feedback loop, goes further still—it is a synthesis motivated by combining these demonstrated component capabilities, not itself a configuration that has been implemented and validated end-to-end in the literature reviewed here—a distinction discussed further, together with the specific feasibility barriers of each block, alongside Figure 16.

A further gap in the discussion above is that it treats each digital twin prediction largely as a point estimate, when in practice any fatigue management digital twin is a chain of coupled uncertainty sources that should be propagated through to the remaining life estimate rather than left implicit. Measurement uncertainty enters through the strain, acoustic, or displacement sensors that feed the twin, and is compounded by sensor degradation or outright sensor failure over the years-to-decades service life the twin is meant to track, which can silently bias the state estimate if not explicitly monitored. Model form uncertainty arises because the physics-based component of the twin (e.g., the fracture-mechanics or finite-element model updated in the case studies above) is itself an approximation of the true structural response, and because the machine learning component, where present, is only as reliable as its training distribution allows. Scatter in fatigue parameters—the same batch-to-batch and process-to-process variability in S-N and crack growth data discussed in Section 6 and Section 9.3—propagates directly into the predicted life whenever the twin’s physics-based component relies on a representative rather than a joint-specific S-N curve. Incomplete loading histories are a related but distinct issue; sensor downtime, data transmission gaps, or simply a monitoring campaign that starts after the structure has already accumulated undocumented fatigue damage all leave gaps in the load spectrum the twin uses to update its state, introducing a source of error that is easy to overlook precisely because it leaves no obvious signature in the output. Where these sources are addressed at all in the case studies discussed above, it is typically only individually and only for the specific uncertainty type each study was designed to demonstrate (for example, Wang et al. propagate model form uncertainty [117], but the case studies here do not jointly propagate measurement, model form, or parameter scatter uncertainty through to a single calibrated confidence interval on remaining life); a digital twin intended for practical fatigue management would need to combine all of these sources, for instance through a Bayesian or ensemble framework that yields a defensible confidence interval on remaining life rather than a single deterministic number, together with explicit sensor health monitoring so that degraded or failed sensors are flagged rather than silently trusted. This more complete treatment of uncertainty propagation remains largely unaddressed in the welded metal sheet digital twin literature reviewed here, and is accordingly included among the research gaps identified in Section 9.3.

#### 8.1.3. Data Augmentation

*[A supporting technique for the fatigue life prediction and crack size estimation tasks above, rather than a task in itself.]* Experimentally, fatigue datasets are expensive to generate, so techniques such as SMOTE (Synthetic Minority Over-sampling Technique) and Z-parameter model fitting are extensively used to expand small datasets while preserving the physical correlation structure between variables demonstrated for E36 steel welded joints, where a 23-point dataset was expanded to 280 samples without losing fidelity to the original Pearson correlation patterns [119]. Because synthetic samples are generated by fitting a model to the existing data rather than by new physical testing, augmentation of this kind can only reproduce trends already present in the original dataset; it does not substitute for experimental validation, and over-reliance on augmented data risks reinforcing spurious correlations.

#### 8.1.4. Explainable and Multi-Scale AI

*[Primarily addressing fatigue life prediction and damage classification, with an emphasis on interpretability for certification.]* Explainability matters in this domain for a specific reason: a fatigue life prediction that determines an inspection interval or a component’s service life must be defensible to a certifying authority, and a black box regression model that cannot show why it predicted a given life is difficult to qualify for safety-critical use, however accurate it is on held-out test data. Feature importance and SHAP-based (SHapley Additive exPlanations) analyses address this by ranking which input variables drive a given prediction, turning the model into a tool that supports engineering root cause analysis rather than replacing it. Kraus and Bartsch demonstrated this directly for welded joints, using automated explainable machine learning combined with systematic feature engineering to discover closed-form fatigue strength models for welded transverse stiffeners, recovering models whose functional form and dominant variables could be inspected and checked against established fatigue mechanics rather than accepted as an opaque prediction [116]. Chen et al. applied a related interpretable surrogate approach to orthotropic steel deck U-rib welds, training a random forest surrogate model that rapidly predicts the stress intensity factor and resulting fatigue life while remaining transparent enough to attribute predictions to specific geometric and loading features, a capability particularly valuable for the bridge deck application discussed further in Section 8.3.3 [118]. Research is moving toward interpretable models that connect microstructural features such as inclusion size, grain structure, and defect distribution to macro-scale fatigue life, rather than treating ML as an opaque black box. Zhou et al. formalized this multi-scale coupling directly, proposing a multiscale modeling strategy that links microstructural damage evolution at the grain and defect scale to macro-scale fatigue-life prediction for welded joints, bridging the gap between the microstructural mechanisms discussed in Section 3 and Section 4 and the component-level life estimates produced by the prediction methods in Section 6 [113]. This traces back to decades of cumulative damage and S–N curve research that emerging AI methods now aim to make more automatable and self-correcting [166].

#### 8.1.5. Transfer Learning

*[Primarily addressing fatiguelife prediction under limited target domain data.]* A complementary strategy for the small, scattered datasets typical of welded joint fatigue testing is transfer learning, in which a model pretrained on a data-rich source domain is fine-tuned on a small target dataset rather than trained from scratch. Xiao et al. applied this approach to the fatigue life prediction of corroded bimetallic steel bars using small samples, showing that transferring learned features between related structural fatigue problems can substantially reduce the amount of target domain data needed for accurate prediction, a strategy that is directly transferable to welded metal sheet structures where large labeled fatigue datasets remain scarce [170]. More directly relevant to welded sheet structures, Schubnell et al. applied transfer learning to the fatigue life assessment of welded steel joints themselves, fine-tuning a model pretrained on non-welded base-material data across 22 welded joint fatigue datasets and demonstrating prediction accuracy comparable to joint-specific S-N curves while substantially reducing the amount of joint-specific testing required, providing direct evidence that the transfer learning strategy generalizes from the corroded bar case above to the welded joint fatigue problem central to this review [171]. Transfer learning is complementary to, rather than competing with, the physics-informed neural networks discussed in Section 5.9; PINNs embed known physical relationships into the model architecture, while transfer learning reuses statistical structure learned from a related dataset, and the two can in principle be combined so that a physics-constrained model pretrained on one joint geometry or material is adapted to another with minimal new fatigue testing.

Table 10 consolidates the machine learning-based strategies discussed across Section 8.1.1, Section 8.1.2, Section 8.1.3, Section 8.1.4 and Section 8.1.5, conventional and hybrid ML, digital twin/Bayesian updating, data augmentation, explainable and multi-scale AI, and transfer learning, summarizing for each the core idea, key strength, key limitation, typical data need, and governing literature.

#### 8.1.6. Uncertainty Quantification and Probabilistic Fatigue Assessment

*[Directly addressing the remaining useful life and maintenance decision support tasks defined at the start of Section 8.1.]* The classical probabilistic fatigue models introduced in Section 6.9—which treat fatigue life and the fatigue limit as random variables rather than fixing them to a single deterministic S-N curve—have a direct counterpart in machine learning-based fatigue prediction. Rather than producing a single point estimate of life, several recent approaches recast the prediction task probabilistically, so that the model’s own uncertainty becomes part of its output rather than an afterthought computed separately. Gaussian process regression, already noted in Section 8.1.1 as one of the standard regression algorithms used for fatigue life prediction, is a natural fit here because it returns a predictive distribution rather than a single value; Bayesian neural networks extend this idea to deeper architectures by placing a distribution over network weights instead of point estimates, at the cost of substantially higher training complexity. A simpler and increasingly common alternative is deep ensembles, in which several independently trained networks are combined, and the spread across their individual predictions is used as a practical proxy for epistemic (model-form) uncertainty without requiring a fully Bayesian formulation. Conformal prediction has also begun to appear in the broader structural fatigue literature as a distribution-free way of wrapping a calibrated confidence interval around the output of an otherwise-arbitrary point-prediction model, which is attractive precisely because it can be layered onto the SVR, ANN, and random forest models already discussed in Section 8.1.1 without retraining them from scratch. These uncertainty-quantification methods connect directly to two other parts of this review; they are the natural mechanism for supplying the calibrated confidence interval on remaining life that Section 8.1.2 identified as missing from current digital twin case studies, and they are a prerequisite for the maintenance decision support task defined at the start of Section 8.1, since a risk-based maintenance decision requires a probability distribution over remaining life rather than a single deterministic number. Despite this relevance, uncertainty-aware machine learning models remain markedly less common in the welded joint fatigue literature than their point prediction counterparts, and rigorous, out-of-distribution calibration checks—confirming that a model’s stated confidence interval actually contains the true life at the stated rate when applied outside its training distribution—are rarer still; this gap is consistent with, and reinforces, the generalization concerns raised in Section 8.1.1 and the joint uncertainty–propagation gap identified in Section 9.3.

### 8.2. Sustainability in Welding and Fatigue Design

The Sustainability branch of Figure 15 reflects the growing recognition that fatigue-durable welds must also be evaluated on energy, material, and environmental cost, not fatigue performance alone. This economic and environmental dimension is not a separate concern from fatigue life prediction, but is coupled to it; a joining process or joint geometry chosen purely to minimize fatigue risk may carry a heavier energy or material cost, so the prediction methods reviewed throughout this article increasingly need to report fatigue performance alongside the process metrics summarized below, rather than in isolation.

However, beyond this process selection trade-off, the sustainability case for accurate fatigue prediction runs in the opposite direction, and more directly; the prediction methods that are the central subject of this review—from the conventional S-N and fracture mechanics approaches of Section 6 through the digital twin and machine learning methods of Section 8.1—are themselves a sustainability lever, independent of which welding process is chosen. A structure whose remaining fatigue life is predicted with a narrower, better-calibrated uncertainty band, of the kind discussed in Section 8.1.6, can be operated closer to its true safe limit rather than being retired or reinforced against a wide conservative margin, directly translating into service life extension without necessarily changing the joint or process at all. The same improvement in prediction confidence supports a reduction in the material used for a given design life, since less conservative safety factors on fatigue-critical members translate into thinner sections or fewer reinforcing details, connecting directly to the lightweighting drivers discussed in Section 1.1. Condition-based monitoring and remaining useful life estimation, discussed in Section 7 and Section 8.1.2, allow inspection intervals to be set from a structure’s actual predicted condition rather than a fixed conservative schedule, reducing inspection frequency and the associated cost, downtime, and travel/energy burden of inspection campaigns, particularly for geographically distributed assets such as the bridges and energy structures discussed in Section 8.3. The same RUL and uncertainty estimates, combined with the maintenance decision support task defined in Section 8.1, enable optimized, risk-based maintenance scheduling in place of fixed-interval servicing, and, where a structure approaching its nominal design life is shown by an updated, more accurate assessment to retain adequate remaining capacity, can avoid an unnecessary replacement altogether. This last effect is the most consequential for life-cycle environmental impact; because manufacturing a replacement welded structure carries its own embodied carbon burden from raw material extraction, processing, and fabrication, of the kind quantified by the life-cycle assessment framework in Section 8.2.2, avoiding or deferring that replacement through more accurate fatigue-life prediction avoids the associated embodied-carbon emissions outright, rather than merely shifting them, in a way that a more sustainable welding process alone cannot achieve once a structure is already in service. Read together, service-life extension, material savings, reduced inspection frequency, optimized maintenance, and avoided replacement are the mechanisms by which improved fatigue life prediction reduces the life-cycle environmental impact of a welded structure, complementing rather than substituting for the process-level sustainability measures discussed in the remainder of this section.

#### 8.2.1. Friction Stir Welding (FSW) for Sustainable Manufacturing

Friction stir welding (FSW) is identified as a leading “green” technology, using approximately 42% less energy than conventional gas metal arc welding (GMAW) when used regularly, while also entirely eliminating filler material and shielding gas consumables [172]. This energy advantage follows directly from the solid-state joining mechanism described in Section 3.5; because the workpiece is plastically deformed rather than melted, FSW requires no arc maintenance power, no shielding gas flow, and no filler wire feeding, all of which are continuous energy and material draws in fusion processes such as GMAW. The absence of a melt pool also removes the fume- and spatter-generating mechanisms that drive the emission comparisons discussed in Section 8.2.3, and it avoids introducing a compositionally distinct filler metal into the joint, which simplifies the recycling of the welded structure at end of life since the joint region does not need to be separated from the parent material before remelting. These combined energy, emission, and end-of-life advantages are a central reason FSW is now positioned, alongside the industrial applications reviewed in Section 3.5 and Section 8.3, as the reference case against which the sustainability of fusion-based alternatives is benchmarked in Section 8.2.2 and Section 8.2.4.

#### 8.2.2. Life-Cycle Assessment (LCA)

Life-cycle assessment (LCA) provides the formal methodology behind these sustainability comparisons; it accounts for the environmental burden of a welding process across defined stages, typically from raw material and energy extraction through electricity generation, consumable manufacture, the welding operation itself, and in cradle-to-grave studies, end-of-life recycling or disposal, expressed against standardized impact categories such as global warming potential, cumulative energy demand, and resource depletion rather than a single cost figure. Applying this framework to welding processes shows that electricity consumption and filler materials dominate the environmental footprint of arc welding processes, and multi-dimensional assessment frameworks are now used to rank welding processes quantitatively, rather than by cost alone [172]. Parisi and Di Bella extended this LCA framing to the end-of-life stage specifically, reviewing how extended producer responsibility regulations and joining method choice jointly determine whether a welded metal structure can be economically disassembled, sorted, and recycled at end of life, an outcome strongly influenced by whether the joint is a dissimilar-filler fusion weld or a filler-free solid-state weld of the kind discussed in Section 8.2.1 [173]. Because LCA results depend heavily on the system boundary and functional unit chosen, comparisons across studies should be read as illustrating the relative ranking of processes, rather than providing universally transferable absolute figures.

#### 8.2.3. Emission Reduction

Cold metal transfer (CMT), a process variant, reduces fume generation relative to older shielded metal arc methods, and also shows improved energy efficiency, thereby directly lowering the health and environmental burden of welding operations [172]. The scale of this reduction is substantial in quantitative terms; fume generation rates fall from roughly 13 mg of fume per gram of electrode consumed for shielded metal arc welding to approximately 1 mg per gram for CMT, an order-of-magnitude reduction that is particularly relevant given that welding fume is classified as carcinogenic to humans, and manganese-bearing fume in particular is linked to neurological effects in exposed workers [172]. CMT achieves this by using a controlled, low-spatter dip-transfer arc cycle with markedly lower heat input than conventional spray-transfer GMAW, which simultaneously reduces the fume-forming vaporization of the electrode and filler wire and lowers the energy drawn per unit length of weld, so the emission and energy benefits are mechanistically linked rather than independent improvements. Because lower heat input also produces a narrower heat-affected zone and less distortion—the same heat-input-driven mechanism that governs weld quality throughout this review—emission reduction in this process family is generally achieved without the fatigue-performance penalty that can accompany other low-heat-input strategies, making CMT one of the few sustainability interventions reviewed here that does not require a trade-off against the fatigue life objectives central to this review.

#### 8.2.4. Circular Economy

Multi-criteria decision-making (MCDM) techniques, such as grey relational analysis (GRA), TOPSIS, and the entropy weighting method, are being used to formally compare FSW against self-piercing riveting and adhesive bonding across sustainability indicators including recyclability, toughness, and joint reusability, providing a quantitative basis for selecting the most sustainable joining method for a given application [174].

### 8.3. Industrial Applications of Fatigue Life Prediction

The industrial applications branch shows that fatigue-critical welded joints span nearly every heavy-industry sector, and prediction models are increasingly tailored to different industries’ needs. The four sectors reviewed below, automotive and rail, aerospace and marine, bridges and infrastructure, and energy structures, differ substantially in the loading spectra their welds experience (high-cycle vibration versus low-frequency, high-amplitude wave and wind loading), in the accessibility of critical welds for in-service inspection, and in the consequence of a fatigue failure, which together determine which of the prediction methods reviewed in Section 6 and Section 8.1 is most appropriate for a given sector. Automotive and rail structures generally favor fast, data-driven regression models suited to high-volume production and frequent design iteration; aerospace, marine, and energy structures, where failure consequences are severe and redesign cycles are long, rely more heavily on fracture mechanics-based and probabilistic reliability methods; and bridge infrastructure, where welds are numerous, largely inaccessible, and must remain in service for decades, is emerging as the leading application domain for the digital twin and structural health-monitoring approaches discussed in Section 8.1.2.

#### 8.3.1. Automotive and Rail

In automotive structural manufacturing, GMAW lap joints are the predominant configuration, and statistical regression models built from 87 experimentally derived S-N curves can now predict fatigue life, achieving coefficients of determination above 0.86. Continuous welded rail and bogie-frame components face similar treatment under high-speed, heavy-haul loading conditions [55]. Automotive fatigue design has additional drivers beyond the joint-level statistical models above; vehicle lightweighting targets push designers toward thinner sheet gauges and mixed-material joints, both of which raise local stress concentrations at the weld toe relative to conventional gauges. The shift toward electric vehicles has also introduced new fatigue-critical welded details, such as battery enclosure seams and structural joints carrying battery pack mass, that were not present in earlier internal combustion architectures and are still accumulating a dedicated fatigue-testing-related evidence base. In rail applications, bogie frames are a particularly demanding case because they combine high-cycle vibration from wheel–rail contact with lower-frequency, high-amplitude loading from braking and curve-negotiation, and because a fatigue crack in a bogie weld is safety-critical and difficult to detect visually once painted over. This has motivated in-service monitoring approaches such as the ultrasonic guided wave crack detection scheme developed by Yan et al. specifically for bogie frame weld zones, which allows an incipient fatigue crack to be identified while the vehicle remains in revenue service rather than only during scheduled overhaul [14].

#### 8.3.2. Aerospace and Marine

E36 steel, widely used in shipbuilding and offshore structures, has been the subject of dedicated ML-based fatigue frameworks addressing crack initiation at weld toes and heat-affected zones [119]. In aerospace and marine applications more broadly, fracture mechanics-based prediction remains central, particularly for FSW joints and butt-welded components subject to variable-amplitude loading [166]. Because ships and offshore platforms operate across a wide temperature range, low-temperature behavior is a further design consideration for these steels; Wang et al. tested EH36 base-metal and T-joint fillet-welded specimens at room temperature and −40 °C, and found that fatigue life at low temperatures was not shorter than at room temperature, though the design S-N curve calibrated at room temperature was conservative when applied to the low-temperature data [175].

#### 8.3.3. Bridges and Infrastructure

Orthotropic steel deck U-rib welds remain the most extensively studied industrial fatigue application, owing to their susceptibility to crack initiation under repeated traffic loading, with machine learning models applied to crack-depth and remaining life prediction for both single- and double-sided U-rib welded joints [18]. These welds are a particularly demanding case for the prediction methods reviewed throughout this article; traffic loading produces a very high number of small-amplitude stress cycles concentrated at the U-rib-to-deck weld root, the weld root itself is inaccessible for direct visual inspection once the deck is in service, and a growing crack can propagate through the deck plate before it is detected by conventional means. Together, these factors explain why bridge infrastructure has become a leading proving ground for the interpretable and digital twin-based methods introduced in Section 8.1. Chen et al.’s interpretable random forest surrogate, discussed in Section 8.1.4, was developed specifically for this detection problem; it predicts the stress intensity factor and resulting fatigue life of double-sided U-rib welds from routinely measured structural response data, offering inspection-relevant predictions at a fraction of the computational cost of a full finite-element crack growth simulation [118]. Lao et al.’s digital twin framework, introduced in Section 8.1.2, was in fact developed and demonstrated on exactly this class of orthotropic deck weld, giving bridge owners a live, continuously updated remaining life estimate for the U-rib weld zone, rather than a fixed inspection interval schedule [168]. Beyond orthotropic decks, the same monitoring and interpretable modeling philosophy is beginning to extend to other welded bridge details, such as rib-to-deck penetration welds where the achieved weld penetration rate during fabrication has itself been shown to materially affect the resulting fatigue life, and should be accounted for explicitly in life-prediction models rather than assumed to be uniform [18].

#### 8.3.4. Energy Structures

Offshore wind turbine jacket foundations and pipeline weldments are assessed using probabilistic fracture mechanics and Monte Carlo-based reliability models, reflecting the stochastic nature of the wind and wave loading these structures experience over multi-decade service lives [17]. Jacket and monopile foundations for offshore wind turbines present an unusually severe fatigue environment because they must resist combined, only partly correlated wind and wave loading, accumulate on the order of 10^7^–10^8^ stress cycles over a typical 20–25 year design life, and are welded tubular joints in a marine environment where corrosion progressively increases local stress concentration and accelerates crack growth relative to the same detail in air. Probabilistic reliability models are used specifically because deterministic design curves cannot economically capture this combination of load randomness, corrosion rate uncertainty, and joint-to-joint variability in weld quality. Because underwater and splash-zone welds are difficult and costly to inspect, target reliability levels are often maintained through a formal balance between conservative initial design and a planned in-service inspection schedule, informed by the same probability-of-detection reasoning discussed for non-destructive testing in Section 7, rather than by design margin alone. Pipeline weldments face a related but distinct loading pattern, dominated by cyclic internal pressure fluctuations acting on girth welds, where the fatigue life is highly sensitive to weld root geometry and any undercut or lack-of-fusion defects of the kind classified by the ISO 5817 quality levels discussed in Section 1.5, making non-destructive inspection at the fabrication stage as consequential for long-term fatigue reliability as the prediction model used afterward.

### 8.4. Digital Twin Case Studies for Welded Structures

Several recent case studies illustrate how digital twin concepts are moving from a general framework, introduced in Section 1.2, into demonstrated applications for welded structures. Wang et al. developed a digital twin-driven fatigue life prediction approach that explicitly propagates model uncertainty through a finite element-based digital twin, giving a probabilistic rather than single-point fatigue life estimate for structures subject to model form and parameter uncertainty [117]. In bridge engineering, digital twins built from finite element submodels have been used to reconstruct unmonitored structural responses from a limited number of physical sensors, and have been coupled with continuum damage mechanics-based fatigue models to evaluate the fatigue performance of orthotropic steel deck welds where residual stress, vehicle loading, and thermal stress interact near the weld toe [168]. These case studies demonstrate the practical value of combining a reduced-order or finite element structural model with sparse real-time sensor data: the digital twin can estimate stress and damage at locations that are physically inaccessible to sensors, propagate uncertainty in a transparent way, and update its own predictions as new monitoring data become available, which is precisely the capability needed to move the fatigue assessment of welded structures from scheduled inspection towards condition-based and predictive maintenance.

Figure 16a explains how the framework pipeline begins with welding process parameters (heat input, speed, geometry) feeding a multi-physics simulation of thermal, mechanical, and microstructural behavior. This is cross-validated against experimental characterization and SHM data (coupon tests, strain and acoustic sensors), which together train a data/ML surrogate embedded in a digital twin for closed-loop state estimation. The digital twin outputs the fatigue life prediction, which then feeds back (orange arrow) to recalibrate the welding process and simulation stages. Figure 16b demonstrates the details of the coupling that underlies the pipeline; physics and sensing exchange information as SHM data calibrate simulation boundary/loading conditions, while the simulation constrains where and how sensing is physically valid. Physics and data couple as the simulation generates physics-informed synthetic training data for the ML model. Sensing and data couple as ML guides optimal sensor placement while SHM signals supply the features the model learns from. These three bidirectional links, not the pipeline order alone, make the digital twin’s prediction falsifiable and actionable.

Figure 16 presents the proposed architecture as a coherent pipeline; it is important to be explicit that the framework as a whole is a proposed synthesis rather than a fully demonstrated system—individual blocks have been validated independently in the literature reviewed above (e.g., finite element-based digital twins with propagated model uncertainty [117], SHM-informed reconstruction of unmonitored structural response [168]). Figure 16a explains how the framework pipeline begins with welding process parameters (heat input, speed, geometry) feeding a multi-physics simulation of thermal, mechanical, and microstructural behavior. This is cross-validated against experimental characterization and SHM data (coupon tests, strain and acoustic sensors), which together train a data/ML surrogate embedded in a digital twin for closed-loop state estimation. The digital twin outputs the fatigue life prediction, which then feeds back (orange arrow) to recalibrate the welding process and simulation stages. The specific three-way coupling shown in Figure 16b, in which physics, sensing, and data mutually constrain one another in a closed loop, has not itself been demonstrated end-to-end for welded metal-sheet fatigue in a single published study. Each block also carries a distinct feasibility barrier that is not visible in the schematic itself. The Multi-Physics Simulation block requires the coupled thermal–mechanical–microstructural simulation of the welding process at a spatial and temporal resolution fine enough to resolve weld toe residual stress; for realistic sheet geometries, this is computationally expensive enough that it is rarely re-run in real time, so in practice the closed loop feedback (orange arrow) would need to rely on pre-computed simulation libraries or fast surrogate models rather than the full physics solver, at some cost to accuracy. The Data/ML block requires a training dataset large and diverse enough to span the relevant welding processes, joint geometries, and loading conditions; Section 9.3 identifies this data scarcity problem generically across machine learning-based fatigue models, and it applies with particular force here because this architecture additionally needs paired, time-synchronized simulation and sensing data to learn the cross-block couplings, not just input–output fatigue life data. The Sensing block depends on strain, acoustic, or equivalent SHM sensors being installed with sufficient density and reliability to constrain the digital twin, which raises the per-structure instrumentation cost and long-term sensor maintenance burden discussed generically in Section 9.3, and is a materially harder requirement for the distributed weld population of a typical sheet structure than for the single, high-value joints where digital twins have so far been demonstrated. Recognizing these barriers does not diminish the value of the proposed architecture as a target for future work, but it does mean that near-term implementation is more realistically framed as a staged adoption, beginning with offline calibration using historical inspection and simulation data, before progressing to the fully closed-loop, real-time configuration shown in Figure 16.

### 8.5. Cross-Study Comparison of Representative Fatigue Prediction Studies

Section 2, Section 3, Section 4, Section 5, Section 6, Section 7 and Section 8 have, to this point, largely introduced representative studies sequentially within each method family. To make the resulting body of evidence easier to compare directly, Table 11 draws together twelve representative studies discussed across the review, spanning critical plane, numerical/multi-scale, machine learning, digital twin, and transfer learning approaches and reports, outlining the material, welding process, joint geometry, loading conditions, prediction method, dataset size, validation strategy, prediction accuracy, and main limitations for each, as stated in the source study. Entries are marked “Not reported” where the cited study, as discussed in this review, does not state that item explicitly; this is itself informative, since the pattern of missing entries in the accuracy, dataset size, and validation strategy columns is concentrated almost entirely in the digital twin and critical plane rows, empirically illustrating the standardized reporting and validation protocol gap identified in Section 9.3, rather than a gap introduced by this summary. Two studies (Schubnell et al. [171] and the E36 steel augmentation study) report an explicit dataset size because both were selected specifically to demonstrate a data efficiency argument; this is the exception rather than the norm across the field, which reinforces the case made in Section 9.3 for standardized, openly reported fatigue datasets.

## 9. Future Research Directions

Building on the welding process, computational, experimental, and data-driven approaches reviewed in Section 2, Section 3, Section 4, Section 5, Section 6, Section 7 and Section 8, this section draws the review to a close in three steps: Section 9.1 synthesizes the critical findings of the literature, Section 9.2 compares the practical trade-offs between the predictive methodologies discussed, and Section 9.3 identifies the specific research gaps that still limit reliable fatigue life prediction for welded metal sheet structures.

### 9.1. Critical Findings from the Literature

This review confirms that fatigue failure remains one of the leading causes of structural damage in welded metal sheets because welding introduces many irregularities, including stress concentrations, microstructural changes, residual stresses, and manufacturing defects, that lead to crack initiation and subsequent propagation [41]. Classical fatigue prediction methods such as the stress life (S-N) method, fracture mechanics, the strain life approach, and cumulative damage models remain widely used because of their long track record and demonstrated real-world effectiveness, although they often assume simplified loading conditions that may not adequately capture the effects of complex joint geometries, environmental degradation, variable-amplitude loading, and multiaxial stresses [28]. Predictive numerical methods such as CZM, XFEM, and FEA provide more accurate predictions of local stress fields and typical fatigue crack formation in welded joints, while integrating digital twin technology, machine learning, and structural health monitoring has substantially improved fatigue prediction capability by enabling more reliable remaining life prediction. Despite all these advancements, accurately capturing residual stresses and weld irregularities, while preserving computational efficiency, remains an open challenge for researchers [169]; the specific gaps this creates for practical fatigue assessment, and the research directions needed to close them, are examined in Section 9.3.

### 9.2. Comparative Evaluation of Predictive Methodologies

Fatigue life prediction methods for welded structures differ considerably in their underlying assumptions, computational requirements, prediction accuracy, and practical applicability. As discussed, conventional methods are cost-effective and simple, and perform best for components with minimal plastic deformation under high-cycle fatigue. The strain life method improves accuracy for low-cycle fatigue by accounting for plastic deformation, but it requires specific material data. Paris’ law and other fracture mechanics techniques are less effective at predicting crack initiation, but are reasonably effective at estimating crack propagation [43]. Finite element-based methods provide realistic stress and strain fields near welds, and are useful for complex geometries and repeated stress analysis, but they demand considerable computational resources.

Current methods such as CZM and XFEM improve prediction accuracy and reduce the need for repeated remeshing during crack growth simulation, but they require extensive data validation and can be computationally expensive.

Taken together, these trade-offs indicate that no single approach is universally superior; rather, the most defensible choice depends on the specific engineering context, and the literature reviewed above supports a more conditional recommendation than a single preferred method. Physics-based conventional methods (S-N, strain life, fracture mechanics, hotspot and notch stress) remain the most appropriate default where the joint detail falls within the constant-amplitude, well-characterized regime covered by established design codes, where regulatory or certification requirements demand a fully auditable and interpretable basis for the predicted life, or where fatigue data specific to the joint are too limited to train a data-driven model. Numerical methods such as CZM, XFEM, and multi-physics/multi-scale simulation are best suited to cases requiring the resolution of local stress, crack path, or microstructural detail that empirical S-N curves cannot capture, provided the added computational cost is acceptable within the design timeline. Machine learning and hybrid data-driven approaches offer the greatest potential where large, reasonably standardized fatigue datasets already exist or can be generated, and where the governing behavior involves complex, nonlinear interactions among geometry, loading, and material variables that are difficult to capture analytically. However, as Section 9.3 discusses in detail, the small size and limited standardization of available welded joint fatigue datasets, unresolved reproducibility across welding processes and material batches, and the near-absence of industrial-scale validation studies mean that machine learning-based predictions currently warrant validation against, rather than the wholesale replacement of, physics-based methods, particularly for safety-critical components. A hybrid strategy, in which machine learning refines, accelerates, or augments a physics-based model rather than substituting for it entirely, therefore represents the more defensible near-term approach for welded metal sheet structures; the balance can reasonably shift further toward data-driven components as standardized datasets, reproducibility studies, and industrial-scale validation mature.

To consolidate the maturity-based synthesis just discussed into a single point of reference, Table 12 compares the six broad approach families reviewed in this work—conventional models, FEA-based structural methods, CZM/XFEM/multi-scale simulation, SHM/sensor fusion, machine learning, and digital twins—by maturity level, required data, computational demand, current limitations, and expected near-term developments, with pointers back to the specific sections and studies each entry summarizes.

### 9.3. Research Gaps and Outlook

Notwithstanding the substantial progress reviewed above, several gaps continue to limit the reliability of fatigue life prediction for welded metal sheet structures in practical use. First, most validated fatigue models remain calibrated against constant-amplitude laboratory data, while real structures experience variable-amplitude and corrosive service loading. Second, because the residual stress field is rarely measured directly in industry, most fatigue assessments still rely on assumptions, introducing an unquantified source of conservatism or non-conservatism, and this assumption is compounded by the fact that even a well-predicted as-welded residual stress field is rarely updated for the cyclic relaxation, crack-driven redistribution, and mean-stress interaction discussed in Section 5.3, so the residual stress input to most fatigue assessments is both unmeasured and effectively static. Third, machine learning and surrogate models are trained and validated on small amounts of available data, which raises concerns about reproducibility across welding processes, material batches, and geometries, since the field currently lacks large, standardized, and openly shared fatigue datasets. Fourth, digital twin technology and sensor fusion frameworks have proven effective in case studies, but their industrial deployment at larger scale is still constrained by sensor cost, data infrastructure requirements, and, most importantly, the absence of standardized validation protocols for digital-twin fatigue predictions. Fifth, the prediction frameworks reviewed above are calibrated and validated almost entirely against conventionally fusion- or solid state-welded joints; their extension to wire arc additively manufactured (WAAM) welded structures remains comparatively unexplored for welded metal sheet applications, even though Section 3.2 already shows that WAAM’s highly anisotropic, columnar-grained microstructure and process-dependent porosity give fatigue strength that is markedly lower and more scattered than that of conventionally welded or wrought material of the same alloy, a gap the review’s own findings on WAAM fatigue behavior directly motivate, rather than one introduced without prior grounding. Sixth, and related to the fourth gap above, digital twin fatigue frameworks generally do not yet propagate their multiple uncertainty sources—sensor measurement error and degradation, model form uncertainty in the underlying physics or ML component, batch-to-batch scatter in fatigue parameters, and gaps in the loading history used to update the twin—jointly through to a single, calibrated confidence interval on remaining life, as discussed in Section 8.1.2; without this, a twin’s point estimate life prediction cannot be assigned a defensible confidence level for safety-critical decision-making. These gaps are not going unaddressed; standards bodies such as the IIW continue to update their fatigue design and post-weld improvement recommendations as new evidence emerges, for example through periodic revisions of the HFMI treatment guidelines [142], but comparable community-wide validation protocols specifically for machine learning- and digital twin-based fatigue predictions have yet to be established. Addressing these gaps will require joint effort across experimental fatigue testing, residual stress characterization, open data-sharing practices, and the development of standardized validation benchmarks for data-driven and hybrid fatigue prediction methods.

## 10. Conclusions

Fatigue failure is among the leading causes of in-service degradation in welded metal sheets, significantly affecting the stability, longevity, and safety of structures across the automotive, railway, civil engineering, and other industries. This review has outlined fatigue life prediction for welded metal sheet structures across its full pipeline—the welding processes and the material, geometric, and environmental factors that govern fatigue performance (Section 2, Section 3 and Section 4); the computational and conventional prediction methods used to model it (Section 5 and Section 6); the experimental and structural health-monitoring techniques used to validate and track it in service (Section 7); and the emerging AI-enabled, sustainability-driven, and industrial applications that are reshaping the field (Section 8). The critical findings, comparative trade-offs, and research gaps synthesized in Section 9 confirm that welding-induced irregularities, particularly residual stresses, remain the principal factor limiting fatigue life, and that no single prediction method yet captures the full range of geometric, material, and service conditions encountered in practice, emphasizing the need for appropriate material selection, reliable mechanical design, and well-optimized welding parameters.

Rather than restate the specific research gaps and priority directions already detailed in Section 9.3, this concluding synthesis instead classifies the reviewed approaches by their readiness for engineering practice, consistent with the maturity, data, and computational demand comparison in Table 12. Conventional S-N, strain life, and fracture mechanics-based methods, together with the established design codes discussed in Section 1.5 (IIW, DNV-RP-C203, Eurocode 3, AWS D1.1), are sufficiently mature for direct engineering implementation, and remain the most appropriate default for routine fatigue design. Numerical approaches such as CZM, XFEM, crystal plasticity, and multi-physics/multi-scale simulation, together with structural health-monitoring sensor technologies, are technically validated but remain primarily research-oriented in their current form, requiring further gains in computational efficiency and field deployment cost before routine industrial adoption. Machine learning-, digital twin-, and sensor fusion-based fatigue-management frameworks, including the closed-loop architecture proposed in Section 8, are the least mature of the approaches reviewed; they show strong promise in case studies, but depend on the standardized datasets, validation protocols, and field-applicable residual stress measurement practices identified in Section 9.3 before they can be considered ready for wide industrial deployment. Advancing the fatigue management of welded metal sheet structures therefore depends less on any single technological breakthrough than on the systematic maturation of these data-driven and sensing-based approaches to the same level of standardization and trust already established for conventional methods.

## Figures and Tables

**Table 1:** Importance of fatigue life prediction across engineering industries.

Industry	Typical Welded Components	Major Fatigue Loading	Importance of Fatigue Prediction	Consequences of Failure	References
Automotive engineering	Frames, body sheets	Braking, road vibration	Vehicle safety, lightweight design	Structural failure, injury	[11]
Aerospace engineering	Fuselage, fuel tanks	Vibration, pressurization	Airframe service life	Catastrophic failure	[12,13]
Railway industry	Coaches, bogies	Cyclic loads	Reliability, passenger safety	Derailment risk	[14]
Offshore/marine	Ship hulls, platforms	Wave-induced loading	Operational reliability	Structural collapse	[15,16]
Renewable energy	Wind turbine towers	Wind-induced cyclic loads	Extended service life	Reduced energy output	[17]
Civil infrastructure	Bridges, pressure vessels	Traffic loading, thermal cycling	Public safety	Structural collapse	[18]

**Table 2:** Comparative analysis of the main fatigue failure mechanisms in welded sheet structures.

Failure Mechanism	Cause	Location	Effect on Fatigue Life	Mitigation Strategy	References
Crack initiation	Cyclic loading, stress concentration	Weld root and toe	Reduces fatigue life	Improved weld profile	[20]
Crack propagation	Tensile residual and cyclic stress	Along weld	Accumulation of damage	Fracture mechanics-based design, crack monitoring	[21]
Final fracture	Critical crack size under applied load	Remaining ligament	Unexpected catastrophic failure	Regular inspection	[22]
Residual stress effects	Welding thermal cycles	HAZ	Tensile stresses accelerate fatigue; compressive stresses delay it	Stress relief, post-weld heat treatment	[23]
Welding defects	Porosity, lack of fusion, inclusions	Weld root and metal	Early crack initiation	Quality control, optimized welding parameters	[24]
Environmental degradation	Weathering, temperature, hydrogen embrittlement	Exposed weld surfaces	Accelerates crack growth	Protective coatings, corrosion-resistant materials	[25]

**Table 3:** Positioning of the present review relative to five related reviews.

Dimension	[36] Kang and Luo (2020)	[37] Feng et al. (2023, AEM)	[38] Fuštar et al. (2018)	[39] Chen and Liu (2022, FFEMS)	[40] England et al. (2023, Metals)	This Review
Primary structural scope	General welded joints	General welded joints	General welded steel structures	General fatigue-critical parts (not weld-specific)	Welds in general (steel and non-ferrous alloys)	Thin welded metal sheets (dedicated focus)
Welding process selection and process-induced factors	–	–	~ (code context only)	–	~ (specimen fabrication only)	✓
Conventional (S-N/fracture mechanics/code-based) methods	✓ (principal focus)	~ (background only)	✓ (principal focus, most detailed)	–	~ (background only)	✓
Machine learning/data-driven prediction methods	–	✓ (principal focus, most detailed data-pipeline treatment)	–	✓ (principal focus, most detailed NN-architecture treatment)	–	✓
Experimental characterization and fractography	~	~	~	–	✓ (principal focus)	✓
Structural health monitoring/in-service sensing	–	–	–	–	–	✓
Very-high-cycle fatigue (VHCF) regime	–	–	–	–	✓ (principal focus, most detailed)	~
Closed loop: simulation + sensing + ML integration	–	–	–	–	–	✓
Sustainability and intelligent fatigue management practices	–	–	–	–	–	✓

Legend: ✓ = principal focus, covered in depth; ~ = covered briefly or as background; – = not covered. None of the five works above combines a thin-sheet-specific scope, a full welding-to-sustainability process pipeline, and an explicit simulation–sensing–machine learning closed loop within a single framework; each covers at most one or two of these three dimensions in depth. The novelty of the present review lies specifically in this combination, not in any one dimension considered in isolation.

**Table 4:** Fatigue crack propagation behavior.

Phase of Fatigue Crack Propagation	Description	Characteristics	Influence on Structural Integrity	References
Phase A: Crack initiation	Micro cracks initiate at metallurgical defect sites such as the weld toe, weld root, or welding defects under repeated cyclic loading	Formation of persistent slip bands (PSBs), micro cracks, localized plastic deformation	No observable damage, but fatigue life begins to decline	[20]
Phase B: Stable crack growth	The crack grows progressively with each loading cycle in a direction roughly normal to the maximum tensile stress	Growth follows Paris’ law and depends on the stress intensity factor range	Crack becomes detectable by ultrasonic inspection or dye-penetrant testing	[43]
Phase C: Accelerated crack growth	As crack length increases, the stress intensity factor rises, leading to a rapid increase in crack growth rate	Crack grows increasingly fast; load-carrying capacity of the section decreases significantly	Component is at risk of catastrophic failure if not repaired or replaced	[41]
Phase D: Fracture	The remaining uncracked area cannot sustain the applied load	Catastrophic failure with minimal plastic deformation (brittle materials) or necking (ductile materials)	Total breakdown, requiring complete replacement of the failed component	[44]

**Table 5:** Comparative fatigue performance of the welding processes, ranked qualitatively via the process-level discussion, alongside a representative quantitative IIW/Eurocode FAT class for a typical joint of each process type. Thickness ranges reflect typical industrial application windows for each process, rather than a hard process limit.

Welding Process	Relative Fatigue Strength	Typical FAT Class *	Dominant Defect/Failure Mode	Typical Crack Initiation Site	Typical Applicable Sheet Thickness *	References
Resistance spot welding (RSW)	Lowest of the processes compared	FAT 36 (steel; often assessed via load-range vs. life rather than nominal stress)	Notch effect at the nugget periphery; discrete-spot stress concentration	Nugget edge/faying-surface periphery	Thin sheet, approx. 0.5–3 mm	[49,50,51]
Gas metal arc welding (GMAW/MIG)	Low-to-moderate	FAT 71 (steel, load-carrying fillet, as-welded)	Weld toe undercut and a relatively wide heat-affected zone	Weld toe (geometric discontinuity)	Thin-to-medium sheet/plate, approx. 1–12 mm	[11,55,56]
Tungsten inert gas welding (TIG)	Moderate; better than MIG owing to lower defect levels	FAT 90 (steel, full-penetration butt weld, as-welded)	Occasional porosity/penetration defects, but generally cleaner welds	Weld toe	Thin sheet, approx. 0.5–6 mm	[58,65]
Hybrid laser–arc welding (HLAW)	High	FAT 100 (steel, full-penetration butt weld, high quality)	Defects from poor laser–arc coordination if process is not optimized	Weld toe/fusion zone boundary	Medium-to-thick plate, approx. 3–20 mm	[61,62,63]
Laser beam welding (LBW)	High	FAT 100 (steel, narrow full-penetration butt weld)	Incomplete penetration, misalignment, porosity	Fusion zone boundary/occasional sub-surface porosity	Thin-to-medium sheet, approx. 0.5–10 mm	[52,53]
Friction stir welding (FSW)	Highest of the processes compared	FAT 71 (aluminum, solid-state butt weld, per Eurocode 9)	Solid-state process; largely free of porosity and solidification cracking	Thermo-mechanically affected zone (TMAZ)	Sheet-to-plate, approx. 1–50 mm (mainly Al alloys)	[59,60]

* FAT class = characteristic fatigue strength (MPa) at 2 × 10^6^ cycles, 95% survival probability, for a representative, good-quality joint of the type typically produced by each process, per IIW recommendations (steel) or Eurocode 9 (aluminum, for FSW). Actual classification depends on the specific joint detail, plate thickness, and quality level; steel and aluminum FAT values are given on different material-specific scales and are not directly comparable in absolute terms.

**Table 6:** Relative influence of various factors on the fatigue performance of welded metal sheets.

Factor	Relative Impact	Key Effect	References
Weld bead geometry	Very high	Stress concentration at the weld toe	[75]
Tensile residual stress	Very high	Accelerates crack propagation	[23]
Surface/weld defects	Very high	Early onset of cracking	[82]
Loading spectrum	Very high	Governs the rate of fatigue damage accumulation	[85]
Heat-affected zone	High	Microstructural deterioration	[88]
Material properties	High	Governs toughness and fatigue strength	[70]
Geometric discontinuities	High	Produces stress concentrations	[73]
Environmental exposure	Moderate to high	Oxidation and corrosion fatigue	[84]
Sheet thickness	Moderate	Modifies stress distribution	[72]

**Table 7:** Method–input–cost–applicability comparison of the numerical and multi-scale modeling approaches.

Method	Required Input Data	Computational Cost	Applicable Scope	Key Limitation	References
Extended finite element method (XFEM)	Uncracked FE mesh with tip-enrichment functions; fracture toughness or Paris law constants; assumed/detected initial crack location and orientation	Moderate (avoids remeshing, but enrichment adds degrees of freedom around each crack)	Arbitrary, mesh-independent crack path propagation through complex or heterogeneous weld geometries	Requires a pre-existing or externally detected initial crack; enrichment function selection and convergence become non-trivial in 3-D weld geometries; addresses propagation rather than initiation	[108,109]
Cohesive zone modeling (CZM)	Traction–separation law parameters (cohesive strength, fracture energy, mixed-mode ratio); cohesive elements placed along a pre-defined interface	Moderate (dedicated cohesive elements along the anticipated crack path)	Interfacial failure and delamination at the weld toe/root; composite and dissimilar material joints	Crack path must be pre-defined along the cohesive interface, limiting arbitrary crack trajectories; traction–separation parameters are difficult to measure directly and are often back-calibrated to match experimental results	[5,110]
Crystal plasticity/microstructure-based simulation	Crystallographic orientation/texture data (e.g., EBSD maps), slip-system parameters, grain boundary properties	High (grain-resolved mesh with nonlinear crystal plasticity constitutive law)	Microstructural-scale crack initiation prediction; alloy and microstructure design for improved fatigue resistance	Computational cost is prohibitive for full-scale structural components; analyses are typically restricted to representative volume elements (RVEs) rather than whole welded joints	[111]
Multi-physics/multi-scale modeling frameworks	Welding process parameters (heat input, speed, geometry); coupled thermal–mechanical–microstructural models spanning micro/meso/macro scales	Very high (multiple coupled solvers across scales, often combining FEA, CFD, and fracture mechanics)	Full welding-to-service-life pipeline; digital twin-oriented frameworks integrating process, microstructure, and structural-scale fatigue prediction	Framework complexity and computational cost limit real-time or routine design use; simultaneous validation across all scales within a single study is rare, so scale-to-scale error propagation is often not directly quantified	[95,112]

**Table 9:** Applicability of S-N, strain life, fracture mechanics, cohesive zone, XFEM, and monitoring-based approaches to crack initiation, short crack propagation, and long crack propagation.

Method	Crack Initiation	Short Crack Propagation	Long Crack Propagation	Primary Limitation Across Stages
Stress life (S-N) method	Implicitly included (total life approach; dominates fatigue life in the HCF regime)	Not resolved explicitly (lumped into total life)	Not resolved explicitly (lumped into total life)	Cannot separate the stages; gives no information on crack size or remaining life once cracking is detected
Strain life (ε-N) method	Primary regime addressed (LCF, plasticity-dominated initiation)	Partially, via life to an assumed “engineering” crack size (often 1–3 mm)	Not addressed; must transition to a fracture mechanics method	Blurs the initiation/short crack boundary into a single empirical “failure” crack size rather than separating them mechanistically
Fracture mechanics—LEFM/Paris’ law	Not addressed (assumes a pre-existing initial flaw)	Not directly valid: LEFM underestimates growth rate and overestimates the threshold ΔK for short cracks (Kitagawa–Takahashi anomaly)	Primary regime addressed (Region II Paris law growth)	Requires an assumed initial crack size; invalid once cracks are physically or microstructurally short
Short crack/elastic–plastic fracture mechanics extensions	Bridges from initiation (models micro crack growth from a nucleated defect)	Primary regime addressed (crack closure-corrected and Kitagawa–Takahashi-based threshold models)	Transitions into standard LEFM once crack closure fully develops	Less standardized than LEFM; requires closure and microstructural barrier parameters that are harder to obtain than bulk material properties
Cohesive zone modeling (CZM)	Can represent (traction–separation law captures gradual degradation from an undamaged interface)	Can represent (no LEFM singularity assumption, valid at small crack lengths)	Can represent (cohesive elements continue along the pre-defined interface)	Crack path must be pre-defined along the cohesive interface; harder to apply where the initiation site is not known a priori
Extended finite element method (XFEM)	Not typically addressed (requires enrichment around an existing/assumed crack)	Feasible with fine tip enrichment, but less commonly demonstrated than for long cracks	Primary regime addressed (mesh-independent long crack path propagation)	Needs an initial crack to enrich around; enrichment/convergence becomes harder at very short crack lengths
SHM/NDT monitoring approaches	Detection threshold generally above initiation for conventional NDT; some advanced methods (e.g., acoustic emission) may detect near initiation	Detection capability strongly method-dependent; most conventional NDT cannot reliably resolve cracks at this size	Primary regime addressed (most SHM/NDT methods are designed and validated for reliably detectable crack sizes)	Provides detection, not prediction; must be paired with a fracture mechanics- or S-N-based method to convert a detected crack into a remaining-life estimate

**Table 10:** Comparison of machine learning-based fatigue life prediction strategies.

Method	Core Idea	Key Strength	Key Limitation	Typical Data Need	References
Conventional ML/hybrid	Regression or classification models (RF, SVR, ANN) fitted to fatigue features	Fast to train; interpretable feature importance	Accuracy limited outside the training data range	Hundreds of labeled S-N points	[166]
Digital twin/Bayesian updating	Physics-based fatigue model, updated with in-service sensor data	Live remaining-life estimate under uncertainty	Requires permanently installed sensing infrastructure	Monitoring time series plus a validated model	[117,165,166]
Data augmentation	Synthetic samples generated from a fitted distribution (e.g., SMOTE, Z-parameter model)	Expands small datasets without new physical testing	Cannot introduce information beyond what the original sample contains	Small original dataset (tens of points)	[119]
Explainable/multi-scale AI	Models linking microstructural features to macro-scale fatigue life with interpretable outputs	Builds engineering trust; supports root cause analysis	Added architectural and training complexity	Moderate; needs microstructural feature data	[113,116]
Physics-informed neural network	Governing fatigue–physics relationships embedded in network architecture or loss function	Physically consistent predictions; extrapolates better than pure ML	Requires an explicit physical model of the failure mechanism	Small to moderate; physics reduces data need	[120]
Transfer learning	Model pretrained on a data-rich source domain, fine-tuned on the target joint or material	Cuts data requirement for new joint types or materials	Prediction quality depends on similarity between source and target domains	Very small target dataset plus one related source dataset	[170]

**Table 11:** Cross-study comparison of twelve representative fatigue prediction studies discussed in Section 6, Section 7 and Section 8, summarizing material, welding process, joint geometry, loading conditions, prediction method, dataset size, validation strategy, prediction accuracy, and main limitations as reported in each source.

Study	Material	Welding Process	Joint Geometry	Loading Conditions	Prediction Method	Dataset Size	Validation Strategy	Prediction Accuracy	Main Limitations
Susmel et al. [130]	Steel and aluminum	Not reported	Welded connections (general)	Variable-amplitude multiaxial fatigue	Critical plane (Modified Wöhler Curve Method, MWCM)	Not reported	Not reported	Not quantified numerically; introduced as a benchmark criterion	Complex to implement; requires dedicated multiaxial material testing
Quantitative Critical plane review by Ng et al. [129]	Steel and aluminum	Not reported	Welded joints (general)	Constant- and variable-amplitude multiaxial	Findley, Carpinteri–Spagnoli, and MWCM criteria, combined with nominal/hotspot/notch stress and TCD stress analyses	Multiple welded joint datasets (number not reported)	Comparative benchmarking against existing multiaxial fatigue test data	MWCM reported as most consistent overall; no single numeric accuracy given	Findings specific to the criteria/joint types benchmarked; extension to other geometries not established
Beiler et al. [131]	Not reported	Not reported	Welded joints (general)	Multiaxial loading	Hybrid analytical Critical plane model + ML regressors	Not reported	Not reported	Matched or exceeded analytical Critical plane accuracy (qualitative)	Requires multiaxial fatigue training data; fewer assumptions but still data-dependent
Kraus and Bartsch [116]	Not reported	Not reported	Welded transverse stiffeners	Not reported	Explainable ML with automated feature engineering, recovering closed-form fatigue strength models	Not reported	Recovered model form checked against established fatigue mechanics	Not quantified numerically in review	Requires sufficient labeled data to recover a reliable closed-form expression
Chen et al. [118]	Not reported (orthotropic steel deck)	Not reported	Orthotropic steel deck U-rib welds	Not reported (bridge in-service loading implied)	Random forest surrogate model predicting stress intensity factor and fatigue life	Not reported	Not reported	Described as rapid and transparent; no numeric accuracy given	Surrogate accuracy bounded by the finite element training data used to build it
Zhou et al. [113]	Not reported	Not reported	Welded joints (general)	Not reported	Multiscale framework linking grain/defect-scale damage to macro-scale fatigue life	N/A (modeling framework)	Not reported	Not quantified in review	Conceptual coupling across scales; cross-scale validation not detailed
Wang et al. [117]	Not reported	Not reported	General welded structure (demonstration)	In-service loading, continuously monitored	Digital twin with propagated model-form uncertainty and Bayesian updating	Monitoring time series (size not reported)	Uncertainty narrowed continually against new monitoring data	Not quantified numerically	Requires continuous, validated sensor input; uncertainty framework adds complexity
Lao et al. [168]	Steel (bridge deck)	Not reported	Orthotropic steel bridge deck weld zone	Field-measured in-service strain histories	Digital twin continuously updating a fracture mechanics model	Field strain history data (size not reported)	Continuous updating against field-measured data (case-study validation)	Not quantified numerically	Depends on permanently installed, maintained sensing infrastructure
Peng et al. [169]	Not reported	Not reported	General welded structure	Fabrication sequence + in-service loading	Digital twin embedding residual stress as an evolving state variable	Not reported	Not reported	Not quantified numerically	Adds modeling complexity; still depends on sensing infrastructure
Xiao et al. [170]	Corroded bimetallic steel bars	N/A (not a welded joint study)	N/A	Not reported	Transfer learning: source domain pretraining, fine-tuned on small target dataset	Small target sample (size not reported)	Not reported	Reported as substantially improved over training from scratch (qualitative)	Prediction quality depends on similarity between source and target domains
Schubnell et al. [171]	Steel	Not reported	Welded steel joints (general)	Not reported	Transfer learning: pretrained on non-welded base material data, fine-tuned on weld data	22 welded-joint fatigue datasets	Fine-tuned model compared against joint-specific S-N curves	Comparable to joint-specific S-N curves (qualitative)	Still requires some joint-specific fine-tuning data; domain-similarity dependent
Zhu et al. [119]	E36 steel	Not reported	Welded joints (general)	Not reported	Data augmentation (SMOTE, Z-parameter model fitting) prior to ML/analysis	23 original points expanded to 280 synthetic samples	Checked against original Pearson correlation structure for fidelity	Not a prediction accuracy metric; correlation fidelity preserved (qualitative)	Cannot introduce information beyond the original sample; not a substitute for physical testing

**Table 12:** Technological maturity, data and computational requirements, current limitations, and expected near-term developments for the six broad fatigue assessment approach families reviewed in this work.

Approach Family	Maturity Level	Required Data	Computational Demand	Current Limitations	Expected Near-Term Developments	References
Conventional (S-N, strain life, fracture mechanics)	Mature—codified in design standards, routine industrial use	Classified detail category or material S-N/fracture data	Low	Does not explicitly resolve local geometry, imperfections, or residual stress (Section 6.1, Section 6.2 and Section 6.3)	Incremental refinement of design code fatigue classes; better residual stress-aware corrections	[43,121,122]
FEA-based structural methods (hotspot, notch stress)	Mature, but accuracy is geometry-sensitive	As-built weld geometry and a validated finite element mesh	Moderate–high	Accuracy bounded by how closely the FE model matches the as-built profile (Section 6.4 and Section 6.5)	Automated mesh generation from 3-D scans of as-built welds	[126,127,128]
CZM/XFEM/multi-scale simulation	Research to early deployment	Cohesive/fracture parameters, fine mesh, microstructural data	High–very high	Requires a pre-defined or assumed crack path or location (Section 5.10, Table 7)	Faster solvers and reduced-order surrogates to lower computational cost	[108,109,110]
SHM/sensor fusion	Lab- and pilot-validated; not yet industrial scale	Sensor time series plus paired ground truth crack/damage data	Low–moderate (per sensor)	No multi-year, fleet-scale validation with defined detection-rate statistics (Section 7.9)	Standardized long-term, multi-structure validation protocols	[117,151,163]
Machine learning (regression, hybrid, explainable)	Demonstrated per study; generalization largely unproven	Labeled S-N or sensor data, often scarce and geometry-specific	Low–moderate (inference); high (training)	Limited out-of-distribution generalization across geometry, material, and process (Section 8.1.1)	Transfer learning and physics-informed constraints to reduce data dependence	[116,166,170]
Digital twins	Conceptual/early research	Paired simulation, sensing, and operational history data	Very high	No fully integrated, end-to-end validated system yet demonstrated (Section 8.1.2, Figure 16)	Staged adoption; joint propagation of measurement, model form, and parameter uncertainty	[117,168,169]

## Data Availability

No new data were created or analyzed in this study. Data sharing is not applicable to this article.

## Abbreviations

For reference, the abbreviations used throughout this review are listed alphabetically below.

**Abbreviation**	**Full Term**
ae	Acoustic Emission
AI	Artificial Intelligence
ANN	Artificial Neural Network
ASME	American Society of Mechanical Engineers
ASTM	American Society for Testing and Materials
AWS	American Welding Society
BM	Base Metal
CMT	Cold Metal Transfer
CZM	Cohesive Zone Model(ing)
DIC	Digital Image Correlation
EBW	Electron Beam Welding
ECT	Eddy Current Testing
ENS	Effective Notch stress (method)
FAT	Fatigue (detail) class
FBG	Fiber Bragg Grating
FE	Finite Element
FEA	Finite Element Analysis
FEM	Finite Element Method
FSW	Friction Stir Welding
GMAW	Gas Metal Arc Welding
GPR	Gaussian Process Regression
GWUT	Guided Wave Ultrasonic Testing
HAZ	Heat-Affected Zone
HCF	High-Cycle Fatigue
HFMI	High-Frequency Mechanical Impact
HLAW	Hybrid Laser–Arc Welding
IIW	International Institute of Welding
ISO	International Organization for Standardization
LBW	Laser Beam Welding
LCA	Life-Cycle Assessment
LCF	Low-Cycle Fatigue
MCDM	Multi-Criteria Decision-Making
MIG	Metal Inert Gas (welding)
ML	Machine Learning
MWCM	Modified Wöhler Curve Method
PAUT	Phased-Array Ultrasonic Testing
PINN	Physics-Informed Neural Network
RF	Random Forest
RSW	Resistance Spot Welding
SHM	Structural Health Monitoring
SMOTE	Synthetic Minority Over-sampling Technique
SPR	Self-Piercing Riveting
SVR	Support Vector Regression
TIG	Tungsten Inert Gas (welding)
TMAZ	Thermo-Mechanically Affected Zone
TOFD	Time-of-Flight Diffraction
VHCF	Very High-Cycle Fatigue
WAAM	Wire-Arc Additive Manufacturing
WM	Weld Metal
XCT	X-Ray-Computed Tomography
XFEM	Extended Finite Element Method
XRD	X-Ray Diffraction
